# Deciphering functional roles of protein succinylation and glutarylation using genetic code expansion

**DOI:** 10.1038/s41557-024-01500-5

**Published:** 2024-03-26

**Authors:** Maria Weyh, Marie-Lena Jokisch, Tuan-Anh Nguyen, Maximilian Fottner, Kathrin Lang

**Affiliations:** 1 Laboratory for Organic Chemistry, Department of Chemistry and Applied Biosciences, ETH Zurich, Zurich, Switzerland; 2 Department of Chemistry, Laboratory for Synthetic Biochemistry, Technical University of Munich Institute for Advanced Study, Garching, Germany; 3 Present Address: CeMM Research Center for Molecular Medicine, Austrian Academy of Sciences, Vienna, Austria

**Keywords:** Post-translational modifications, Proteins, Chemical modification

## Abstract

Post-translational modifications (PTMs) dynamically regulate cellular processes. Lysine undergoes a range of acylations, including malonylation, succinylation (SucK) and glutarylation (GluK). These PTMs increase the size of the lysine side chain and reverse its charge from +1 to −1 under physiological conditions, probably impacting protein structure and function. To understand the functional roles of these PTMs, homogeneously modified proteins are required for biochemical studies. While the site-specific encoding of PTMs and their mimics via genetic code expansion has facilitated the characterization of the functional roles of many PTMs, negatively charged lysine acylations have defied this approach. Here we describe site-specific incorporation of SucK and GluK into proteins via temporarily masking their negative charge through thioester derivatives. We prepare succinylated and glutarylated bacterial and mammalian target proteins, including non-refoldable multidomain proteins. This allows us to study how succinylation and glutarylation impact enzymatic activity of metabolic enzymes and regulate protein–DNA and protein–protein interactions in biological processes from replication to ubiquitin signalling.

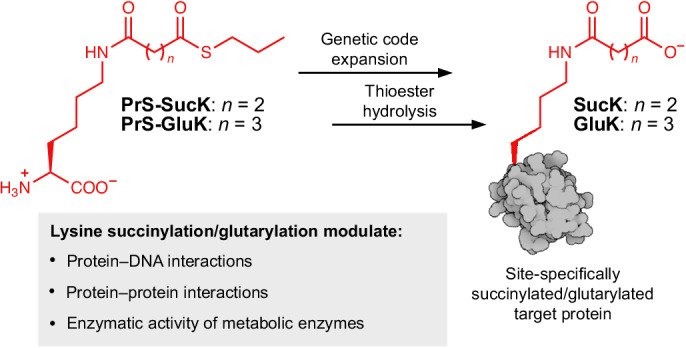

## Abstract (web summary)

Negatively charged lysine acylations—malonylation, succinylation and glutarylation—impact protein structure and function, which can affect cellular processes. Now temporarily masked thioester derivatives of succinylation and glutarylation can be used for site-specific modification of diverse bacterial and mammalian proteins, which can facilitate the study of how these lysine modifications impact enzymatic activity and control protein–protein and protein–DNA interactions.

## Main

**Fig. 1:**
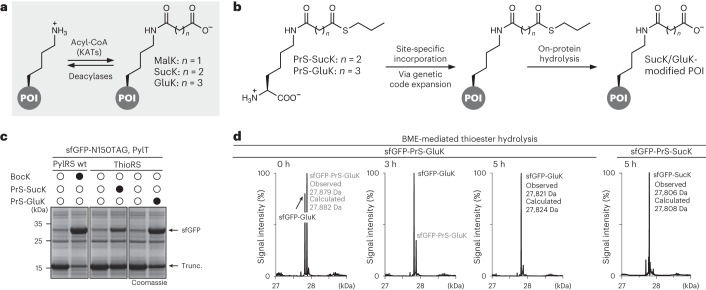
Site-specific installation of acidic lysine acylations via genetic code expansion. **a**, Acidic lysine acylations (MalK, SucK and GluK) are introduced into POIs using acyl-CoA either via lysine acetyl transferases (KATs) or non-enzymatically and can be reverted by deacylases. **b**, PrS-SucK and PrS-GluK are site-specifically incorporated into POIs via genetic code expansion and on-protein thioester hydrolysis affords the succinylated or glutarylated POI. **c**, SDS–PAGE analysis of the expression of sfGFP-N150PrS-GluK and sfGFP-N150PrS-SucK (Trunc. denotes truncated protein). **d**, Left: LC–MS analysis of BME-mediated thioester hydrolysis at physiological pH on sfGFP-N150GluK with samples taken at denoted time points. Right: LC–MS analysis of sfGFP-N150SucK after BME-mediated thioester hydrolysis. The full gels can be found in Supplementary Fig. 13, and non-deconvoluted *m*/*z* spectra can be found in Supplementary Fig. 16. Consistent results were obtained over three distinct replicate experiments. Source data

As the only amino acid with a side-chain primary amine, lysine undergoes a myriad of post-translational acylations, including acetylation, 2-hydroxyisobutyrylation, benzoylation, propionylation, butyrylation and crotonylation, that have been revealed in both histones and non-histone proteins^1–3^. Three of these modifications, namely malonylation (MalK), succinylation (SucK) and glutarylation (GluK) (Fig. 1a), do not only increase the size of the lysine side chain considerably but also reverse its charge from +1 to −1 under physiological conditions. Accordingly, this more dramatical structural change is likely to lead to substantial changes in protein structure and function. Recent advances in mass spectrometry have identified and comprehensively validated MalK, SucK and GluK within thousands of substrate proteins in both prokaryotic and eukaryotic cells^4–8^. MalK, SucK and GluK modifications are abundant among metabolic enzymes and DNA-interacting proteins, such as histones, and are regarded as very dynamic modifications. This suggests that these acidic lysine acylations are probably key players in the regulation of cellular function from altering enzyme activities of metabolic enzymes to controlling protein–protein or protein–nucleic acid interactions in replication and in transcription and as such are associated with various diseases.

Similar to acetylation, acidic lysine acylations are derived from the corresponding short-chain acyl coenzyme A (CoA) metabolites (malonyl-CoA, succinyl-CoA and glutaryl-CoA). Specific writer and eraser enzymes for installation and removal of MalK, SucK or GluK have been identified for just a few target proteins and modification sites, and the functional roles of these three post-translational modifications (PTMs) remain in many cases enigmatic^4,5,9^. One impediment to studying the effects of site-specific MalK, SucK and GluK modifications consists in the difficulty of obtaining homogeneously modified proteins in sufficient yields, as the enzymes responsible for these modifications are often elusive. Furthermore, malonylation, succinylation and glutarylation can also occur via non-enzymatic mechanisms, suggesting that intracellular concentrations of malonyl-CoA, succinyl-CoA and glutaryl-CoA may govern the rates of lysine modification in a pH-dependent manner^6,10,11^. Regardless of the route of addition, these PTMs are believed to heavily modulate protein structure and function and must be dynamically regulated through enzymatic removal via lysine deacylases, such as sirtuins^5,12,13^.

Studies on the effects of succinylation and glutarylation on protein function have so far primarily focused on elucidating their regulatory influence on mitochondrial proteins in living cells^14,15^ as well as on analysing their structural and functional impact on histones and assembled nucleosomes in vitro^16,17^. Histones can be easily refolded, and site-specifically modified variants can therefore be accessed by chemical synthesis coupled to peptide/protein ligation followed by in vitro nucleosome reconstitution^16–18^. An alternative approach used thiol–ene chemistry to modify a histone bearing a single cysteine mutation with a correspondingly modified and protected succinamic acid derivative, affording succinyl-4-thialysine as a SucK analogue (Supplementary Fig. 1)^19,20^. These studies showed that succinylation and/or glutarylation of histones impact nucleosome stability and thereby shape chromatin dynamics. While these approaches are proven tools for studying histone modifications and nucleosome dynamics in in vitro assays, they typically depend on harsh deprotection and/or desulfurization protocols and are therefore not applicable to study the effects of succinylation and glutarylation on complex non-refoldable multimeric proteins.

Many lysine acylations, including acetylation, propionylation, butyrylation, crotonylation, 2-hydroxyisobutyrylation, benzoylation, β-hydroxybutyrylation, lactylation, biotinylation and lipoylation^21–23^, have been site-specifically incorporated into proteins via genetic code expansion approaches in the form of non-canonical amino acids (ncAAs) using orthogonal pyrrolysyl-transfer RNA synthetase (PylRS)–tRNA pairs. Attempts to engineer a specific PylRS variant for the acidic lysine acylations MalK, SucK and GluK have however not been successful so far. This may be attributed to the overall hydrophobicity of the amino acid binding pocket of PylRS and its evolved variants that prefer ncAAs with hydrophobic side chains. Furthermore, cellular uptake of these negatively charged amino acids may be more restricted compared with ncAAs with hydrophobic side chains. To access SucK-modified proteins, a two-step approach has previously been developed that relies on site-specific incorporation of azidonorleucine, followed by a traceless Staudinger ligation with a succinyl-modified and photocaged phosphinothioester. This method enabled generation of succinylated proteins, but in only 30–40% yield and with wild-type lysine-bearing protein as the main byproduct, making it impossible to obtain pure SucK-modified proteins as needed for functional biochemical studies (Supplementary Fig. 1)^24^.

In this Article, we describe an approach for the genetic encoding of temporarily masked SucK and GluK derivatives that allows the generation of homogeneously and site-specifically modified SucK- and GluK-bearing target proteins. We have synthesized thioester derivatives of SucK and GluK that mask the negatively charged side chain carboxylate and show that these ncAAs (PrS-SucK and PrS-GluK; Fig. 1b) can be incorporated into proteins of interest (POIs) in a site-specific manner using an evolved PylRS variant and are quantitatively converted to SucK- and GluK-bearing POIs through thioester hydrolysis on folded proteins. We show incorporation of SucK and GluK into reported positions in various POIs, showcasing the regulatory potential of these PTMs for controlling enzymatic activity and influencing protein–protein and protein–DNA interactions in biological processes ranging from metabolism to replication.

## Results and discussion

### Generating SucK- and GluK-modified recombinant proteins

At the outset of our studies, we decided to concentrate on site-specific incorporation of SucK and GluK via genetic code expansion, as MalK has been reported to be thermally labile due to decarboxylation^25,26^, and we expected the SucK and GluK derivatives bearing longer alkyl chains to be preferred substrates for PylRS and its evolved variants. To direct the site-specific incorporation of SucK and GluK via genetic code expansion, we synthesized a panel of SucK and GluK derivatives that mask the negative charge of the side chain carboxylate, thereby potentially improving cellular bio-availability and forming more favourable substrates for PylRS and its evolved variants. We focused on ester derivatives that would be stable under physiological conditions but cleavable post-translationally on protein by either enzymatic or mild chemical treatment. Different tested oxoester-masked SucK/GluK derivatives were either not substrates for tested PylRS variants or were resistant to hydrolysis after incorporation into POIs requiring very harsh acidic or alkaline treatment, neither of which is suitable for a wide variety of proteins. We therefore turned our attention to thioester-masked SucK/GluK derivatives.

We synthesized S-propyl thioesters of SucK/GluK by coupling succinic or glutaric anhydride to an appropriately protected lysine loaded on a solid support. Steglich esterification with propanethiol, followed by cleavage and deprotection, afforded multigram quantities of PrS-SucK and PrS-GluK (Supplementary Fig. 1b). Subjecting these ncAAs to a screening approach by co-expressing one of the approximately 200 distinct PylRS variants available in the laboratory, together with superfolder green fluorescent protein (sfGFP) bearing a premature amber (TAG) codon, identified a PylRS variant from *Methanosarcina barkeri* (*Mb*) that was able to charge PrS-SucK/PrS-GluK onto its respective tRNA (PylT). This *Mb*PylRS variant bears mutations Y271A and C313V in its active site, as well as the previously reported IPYE mutations in its N-terminus^27^ and was dubbed ThioRS. We showed that ThioRS is efficient in site-specifically incorporating PrS-SucK/PrS-GluK into sfGFP bearing an amber codon at position 150 (Fig. 1c). Transferring the mutations from ThioRS to the PylRS variant from *Methanomethylophilus alvus*^28,29^ (*Ma*) and introducing further mutations that have been found beneficial for incorporation of ncAAs in *Escherichia coli*^30^ (*Ma*ThioRS with mutations Y126A, H227I and Y228P) showed that also this *Ma*PylRS variant is efficient in encoding PrS-SucK/PrS-GluK (Supplementary Fig. 2).

Interestingly, analysis by mass spectrometry (MS) of purified sfGFP that was expressed in the presence of PrS-GluK revealed an approximately 1:1 mixture of GluK-modified sfGFP and the corresponding sfGFP variant with intact S-propyl thioester modification, indicating that the thioester was partially hydrolysed during protein expression and purification. This ratio shifted in favour of GluK-bearing sfGFP upon longer incubation in aqueous buffer at pH 7, and overnight incubation at elevated pH (pH 9) led to quantitative hydrolysis to yield pure sfGFP-N150GluK (Supplementary Fig. 3a–c). To accelerate hydrolysis and inspired by literature on hydrolysing peptide-α-thioesters, we incubated purified sfGFP at pH 7 with 10–100 mM β-mercaptoethanol (BME)^31^. We hypothesized that also on folded proteins, BME may transthioesterify the PrS-GluK thioester, followed by an (S,O)-acyl shift yielding the corresponding 2-mercaptoethanol oxoester of GluK, which should spontaneously decompose through intramolecular displacement of ethylene sulfide to give GluK-modified sfGFP (Supplementary Fig. 3d). Indeed, incubation with 100 mM BME at pH 7 led to quantitatively hydrolysed GluK/SucK-bearing sfGFP within 3–5 h (Fig. 1d and Supplementary Fig. 3e). In the presence of 5 mM tris(2-carboxyethyl)phosphine (TCEP), concentrations as low as 10 mM BME at pH 7 were effective for quantitative on-protein hydrolysis within a few hours (Supplementary Fig. 3f)^32^.

### Recombinantly installed SucK/GluK are deacylase substrates

**Fig. 2:**
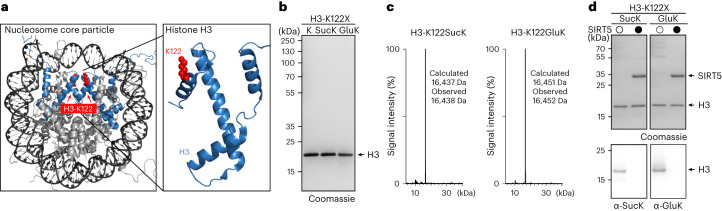
Generation of site-specifically succinylated and glutarylated histone H3. **a**, Structure of the nucleosome core particle, with histone H3 highlighted in blue (Protein Data Bank, 1kx5)^67^. The modified lysine (K122, red) is located at the dyad axis of the histone–DNA interaction. **b**, SDS–PAGE analysis of purified histone H3 variants (WT H3, H3-K122SucK and H3-K122GluK). **c**, LC–MS analysis confirmed the integrity of purified histone variants H3-K122SucK and H3-K122GluK. Comprehensive LC–MS analysis can be found in Supplementary Figs. 4 and 16. **d**, SDS–PAGE and western blot analysis of deacylation assays with H3-K122SucK and H3-K122GluK using SIRT5. The full gels can be found in Supplementary Fig. 13. Consistent results were obtained over three distinct replicate experiments. Source data

Studies on histones have revealed lysine succinylation and glutarylation in both the linker histone H1 and the four core histones H2A, H2B, H3 and H4^2,33^. Site-specifically modified histone proteins are typically accessed by a combination of solid-phase peptide synthesis and native chemical ligation and have elucidated that glutarylation of K91 in H4, as well as succinylation of K77 in H4 and K122 in H3, lead to destabilization of nucleosomes^34–36^. The methods to access site-specifically succinylated or glutarylated histones and nucleosomes remain, however, elaborate and require expert chemistry techniques and are often inefficient. We therefore tested if we could leverage our approach to express H3 in *E. coli* with site-specifically encoded SucK and/or GluK at K122. This residue is situated in the so-called lateral surface region at the dyad axis of the nucleosome, where its side chain is in close contact to the negatively charged DNA wrapped around the nucleosome core particle (Fig. 2a and Supplementary Fig. 4a). Reversing the charge of this lysine residue from +1 to −1 by succinylation destabilizes nucleosomes, as shown by fluorescence resonance energy transfer experiments, utilizing reconstituted nucleosomes with chemically synthesized H3-K122SucK^34^.

We recombinantly expressed H3 bearing PrS-SucK/PrS-GluK at K122 and confirmed specific incorporation and quantitative BME-induced hydrolysis to H3-K122SucK/GluK via MS (Fig. 2b,c and Supplementary Fig. 4b,c). To prove that genetically encoded SucK is indistinguishable from its endogenously installed counterpart, we incubated H3-K122SucK with SIRT5, a deacylase that has previously been shown to desuccinylate H3-K122SucK peptides. Both western blot analysis using a SucK-specific antibody and liquid chromatography (LC)–MS analysis confirmed that fully folded H3-K122SucK is a substrate for SIRT5 (Fig. 2d and Supplementary Fig. 4d)^12,34^. By incubation of glutarylated histone H3 with SIRT5, we furthermore identified SIRT5 as efficient deglutarylase for H3-K122GluK (Fig. 2d and Supplementary Fig. S4d).

SIRT5 has been reported as a promiscuous desuccinylase^12^. The enzyme has an atypical acyl-binding domain that prefers negatively charged residues, such as SucK, over *N*^ε^-acetyl-l-lysine (AcK)^8,12^. To test whether SIRT5 is able to desuccinylate also bacterial target proteins, we produced the bacterial oxidoreductase AzoR (flavin mononucleotide-dependent NADH:quinone oxidoreductase) bearing SucK at position 133 (ref. ^6^) and incubated it with SIRT5 (Supplementary Fig. 5a–c), showing that, in our in vitro assay, SIRT5 acts on bacterial succinylated proteins. The only identified deacylase in *E. coli*, the NAD^+^-dependent class III sirtuin, CobB shares sequence similarity with SIRT5 in its acyl-binding domain^12^, suggesting that CobB may be able to catalyse lysine desuccinylation^8^. Proteomic quantification of succinylation sites in wild type (WT) *E. coli* cells and CobB knockout cells indicated, however, that succinylation was not globally altered by loss of CobB^6^. Interestingly, in our in vitro assay, CobB was able to partially hydrolyse succinyl-lysine within AzoR-K133SucK (Supplementary Fig. 5d).

### Glutarylation modulates the activity of metabolic enzymes

Having established a general tool to site-specifically succinylate and glutarylate various target proteins, we next set out to study the effects of these PTMs on enzyme activity and protein–protein interactions. We screened proteomic databases^37^ and chose interesting target proteins with reported succinylation/glutarylation sites either close to their respective active sites or in interactions sites where we assumed introduction of negative charges may have an impact on enzymatic activity^4–7^.

**Fig. 3:**
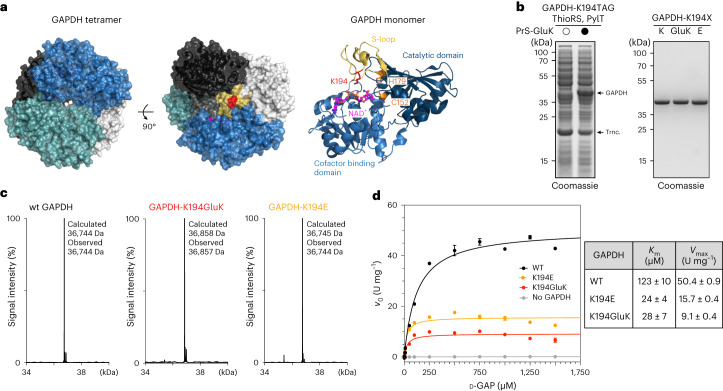
Glutarylation of GAPDH at K194 regulates its enzymatic activity. **a**, Left: Structure of the GAPDH tetramer with the four monomers shown in different colours (blue, black, teal and grey) in two different orientations. Interacting S-loop regions of two adjacent monomers (blue and black) are coloured in yellow and dark grey, respectively. K194 is shown in red, and NAD^+^ in pink (Protein Data Bank, 1znq)^39^. Right: Structure of the GAPDH monomer highlighting the cofactor-binding domain, the catalytic domain and S-loop. The modified residue (K194, red) is located in the S-loop (residues 181–209, yellow) in proximity to the NAD^+^-binding site. The catalytic residues H179 and C152 are shown in orange. **b**, SDS–PAGE analysis of the site-specific incorporation of PrS-GluK into GAPDH at position 194 (left, trnc. denotes truncated GAPDH) and of purified GAPDH variants (right). Consistent results were obtained over three distinct replicate experiments. The full gels can be found in Supplementary Fig. 14. **c**, LC–MS analysis confirmed the integrity of the purified GAPDH variants. The comprehensive LC–MS analysis can be found in Supplementary Figs. 6 and 16. **d**, Analysis of the enzymatic activity and kinetics of WT GAPDH and GAPDH variants according to Michaelis–Menten. Glutarylation of GAPDH at K194 or the GAPDH-K194E mutant as a charge-mimic reduced both *K*_m_ and *V*_max_. The initial reaction velocity (*v*_0_) was plotted against d-GAP concentration and fitted with a Michaelis–Menten model to determine *K*_m_ and *V*_max_ values. Average values and errors (±s.e.m.) were calculated from three biologically independent experiments (*n* = 3). All data processing was performed using GraphPad Prism 10 (GraphPad software). Source data

We first concentrated on the glycolytic enzyme glyceraldehyde 3-phosphate dehydrogenase (GAPDH), a ubiquitous and essential enzyme that catalyses the conversion from d-glyceraldehyde-3-phosphate (d-GAP) to 1,3-bisphosphoglycerate in the presence of inorganic phosphate and the cofactor NAD^+^ (Supplementary Fig. 6a). In addition to this metabolic function, GAPDH has been implicated in several unrelated non-metabolic processes, such as control of gene expression and apoptosis^38^. There is evidence that this functional versatility may be regulated, at least in part, by PTMs that alter its catalytic activity and influence the subcellular localization of the enzyme^38^. Among phosphorylation, acetylation, ubiquitylation and redox-PTMs, GAPDH has been reported to be succinylated at various surface lysines^6^ and glutarylated at K194^5^, a residue close to the active site. In the cytosol, GAPDH forms a homotetramer that is stabilized by several hydrogen bonds between the individual subunits (Fig. 3a)^39^. Each monomer consists of a C-terminal cofactor-binding domain and an N-terminal catalytic domain harbouring the catalytic residues C152 and H179. An important structural feature of the catalytic domain is a long ordered loop called the S-loop (amino acids 181–209) that sits on top of the cofactor and is important for closing of the NAD^+^-binding site (Fig. 3a). This loop extends towards the adjacent monomer stabilizing interactions between subunits positioned across from each other in the tetramer. The amino acids in the S-loop are highly conserved, and we assumed that glutarylation of K194 situated in the middle of this loop might be important for GAPDH activity, although there are no direct interactions with cofactor or active site residues.

We expressed and purified human WT GAPDH and a GAPDH mutant bearing either glutamate (GAPDH-K194E) or GluK at position 194 (GAPDH-K194GluK) heterologously in *E. coli*. (Fig. 3b and Supplementary Fig. 6b,c). In addition, we also prepared a GAPDH variant bearing AcK at position 194 (GAPDH-K194AcK)^40^, as K194 was also found to be acetylated (Supplementary Fig. 6b,c)^6,37^. To determine enzymatic activity of the diverse GAPDH variants, we set up an assay to follow consumption of d-GAP by monitoring spectrophotometrically the fluorescence of NADH at 450 nm upon incubation of GAPDH with NAD^+^, arsenate and increasing concentrations of d-GAP to calculate maximal velocity (*V*_max_) and the Michaelis–Menten constant (*K*_m_) (Fig. 3d and Supplementary Figs. 6a,d, 7 and 8)^41^.

Importantly, thioester hydrolysis (incubation with BME or at pH 9) did not impact the enzyme kinetics of WT GAPDH (Supplementary Fig. 6d). Interestingly, the GAPDH-K194GluK variant showed a four- to five-fold reduction in both *V*_max_ and *K*_m_, indicating that, indeed, glutarylation of this lysine residue impacts enzymatic activity. The glutamate mutant as a mimic for the negatively charged GluK showed similar results (Fig. 3d and Supplementary Fig. 6d). For the less bulky and uncharged AcK-GAPDH variant, on the contrary, we observed only 30–50% lower *K*_m_ and *V*_max_ values compared with WT GAPDH (Supplementary Fig. 6d). Introduction of a bulky group with negative charge into the highly conserved S-loop could either occlude access to the active site by hindering NAD^+^ binding or also impair interactions between the subunits thereby weakening the enzymatically active tetrameric structure of GAPDH.

### Succinylation controls protein–protein interactions

We expected that acidic lysine acylations may be especially important for regulating protein–protein interactions, as they represent quick and reversible means to revert the lysine side chain charge from +1 to −1, apart from introducing also steric bulk. Given recent evidence that PTMs such as phosphorylation and acetylation on ubiquitin (Ub) enhance the complexity of the Ub code^42,43^, we wondered whether the lysine residues in Ub are also targets for succinylation/glutarylation. With the potential exception of K29, all seven lysine residues of Ub can be acetylated^43^, and it has been shown that acetylation at individual Ub lysines impedes E2/E3-mediated assembly of polyUb chains, as neutralization of the positive charges seems to affect non-covalent interactions of Ub with E2/E3-enzymes^44^. From system-wide proteomic approaches it is known that there is an extensive overlap of acetylation with succinylation on multiple prokaryotic and eukaryotic proteins^6^, and indeed, screening PTM databases revealed that Ub lysine residues are also modified by succinylation^37,45,46^. SucK has been detected on K6, K11, K27, K33, K48 and K63. We were especially interested in how these site-specific modifications might govern interaction with specific deubiquitylases (DUBs), as it has been shown that Ub phosphorylations affect DUB activity and specificity^47^.

**Fig. 4:**
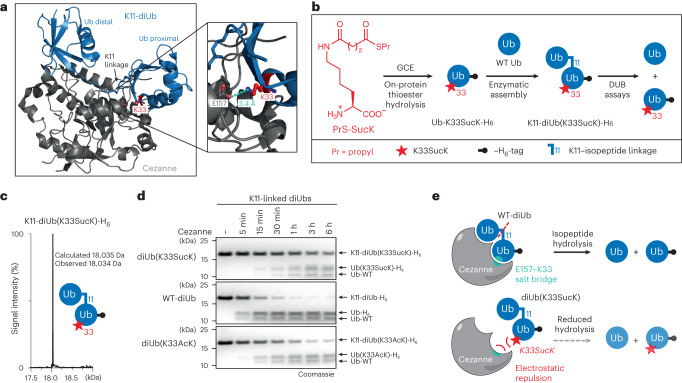
Succinylation of K33 in Ub modulates its interaction with the DUB Cezanne. **a**, Structural insights into the interaction between Cezanne and a K11-linked diUb (Protein Data Bank, 5lrv)^48^. A salt bridge between K33 (red) of the proximal Ub and E157 of Cezanne stabilizes the interaction. **b**, A schematic representation of the generation of K11-diUb(K33SucK)-H_6_. **c**, LC–MS analysis confirms the integrity of the generated succinylated diUb. LC–MS analysis of WT and acetylated diUb can be found in Supplementary Fig. 9, and further comprehensive LC–MS analysis can be found in Supplementary Fig. 17. **d**, SDS–PAGE analysis of a DUB assay time course. K11-diUb(K33SucK)-H_6_, WT K11-diUb-H_6_ and K11-diUb(K33AcK)-H_6_ (all 5 µM) were individually incubated for the denoted time points with Cezanne (7.5 nM). Cezanne-mediated diUb cleavage is most severely impaired by succinylation of K33 in the proximal Ub. The full gels and quantification of bands can be found in Supplementary Figs. 10 and 15. Consistent results were obtained over three biologically independent replicate experiments. **e**, Model of Cezanne K11-diUb interaction displaying the E157-K33 salt bridge that may be modulated by post-translational modification of K33. Source data

Examining structures of linkage-specific DUBs with their respective diUb substrates revealed that an unusual surface of the proximal Ub around K33 makes close contacts with the S1′ site in the K11-specific DUB Cezanne (Fig. 4a)^48^. The direct interaction between residue E157 in Cezanne and K33 in the proximal Ub of K11-linked diUb impacts catalytic turnover of the enzyme and a K11-linked diUb with a K33E mutation in the proximal Ub showed reduced hydrolysis rates compared with WT K11-diUb^48^. As K33 has been reported to be succinylated as well as acetylated^37,45,46^, we expressed and purified Ub-K33SucK and Ub-K33AcK and assembled K11-linked diUbs with WT Ub in the presence of the respective E1 and E2 enzymes (UBE1 and UBE2S), as described previously^49^ (Fig. 4b and Supplementary Fig. 9). We prepared and purified WT K11-diUb (WT K11-diUb-H_6_), as well as a K11-linked diUb bearing SucK or AcK at K33 of the proximal Ub (K11-diUb(K33SucK)-H_6_ or K11-diUb(K33AcK)-H_6_, Fig. 4c and Supplementary Fig. 9). Incubation of SucK-modified K11-linked diUb with Cezanne led indeed to substantially reduced isopeptide bond hydrolysis compared with unmodified K11-diUb (Fig. 4d and Supplementary Fig. 10). Similarly, the K11-linked diUb variant bearing an acetylated lysine residue at position 33 showed impaired Cezanne-induced hydrolysis rates, although less pronounced than the succinylated variant. These data confirm that the direct salt-bridge interaction between E157 in Cezanne and K33 in proximal Ub is important for enzymatic activity of Cezanne and can be modulated by PTMs. (Fig. 4d,e and Supplementary Fig. 10). This reveals a regulatory potential of succinylation in repressing or potentially also increasing DUB activity, as has been shown for phosphorylation^47^.

### Succinylation regulates DNA–protein interactions

Arguably, acidic lysine acylations may exert the biggest effect on nucleic acid–protein interactions, as a charge reversion in a DNA/RNA-interacting protein from +1 to −1 may severely destabilize the corresponding complexes, as has been shown for chemically prepared SucK/GluK-modified histones and their destabilizing effect on nucleosomes^16,17^. Our approach of genetically encoding SucK/GluK has the advantage of being applicable to complex and non-refoldable proteins, including multidomain proteins. We were therefore interested in examining the effect of the reported succinylation sites within the DNA clamp proliferating cell nuclear antigen (PCNA) on DNA loading and replication.

**Fig. 5:**
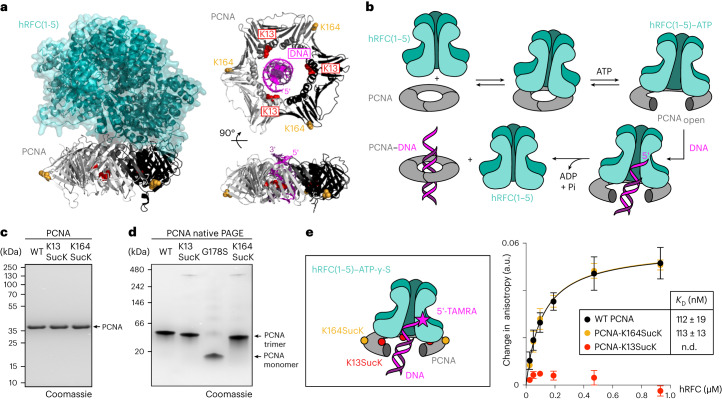
Succinylation regulates hRFC-mediated clamp loading onto DNA. **a**, Left: Structural insights into the PCNA:hRFC interaction. Right: Top and side view of the PCNA:DNA complex. Known succinylation sites (K13 (red) and K164 (yellow)) are highlighted (Protein Data Bank, 1sxj ref. ^51^ and 6gis ref. ^68^). **b**, Schematic representation of the hRFC-mediated charging of PCNA onto DNA. hRFC binds on top of the PCNA ring, and ATP binding triggers opening of PCNA, allowing loading of primer-templated DNA. Upon ATP hydrolysis, PCNA is released bound to DNA. **c**, SDS–PAGE analysis of purified PCNA variants. Consistent results were obtained over three distinct replicate experiments. **d**, Native PAGE analysis of WT PCNA and PCNA variants indicates intact trimerization for the PCNA-SucK variants. G178S is a previously reported PCNA mutant that is deficient in forming stable trimers and was used as control. Consistent results were obtained over three distinct replicate experiments. The full gels can be found in Supplementary Fig. 11. LC–MS analysis of the different PCNA variants can be found in Supplementary Figs. 11 and 18. **e**, Left: Schematic representation of stalled ternary PCNA:hRFC:DNA complex in the presence of non-hydrolysable ATP-γ-S. Right: Fluorescence anisotropy measurements were conducted to determine the *K*_D_ of hRFC and PCNA variants for fluorescently labelled DNA. The change in anisotropy was plotted against hRFC concentration and fitted with a single-site binding model to determine *K*_D_ values. The average values and errors (±s.e.m.) were calculated from three biologically independent experiments (*n* = 3). All data processing was performed using GraphPad Prism 10 (GraphPad software). n.d., not determined; a.u., arbitrary units. Source data

PCNA is a ring-shaped, homotrimeric protein, essential to all living organisms, that plays a crucial role in DNA replication and in maintaining genome integrity^50^. As a molecular sliding clamp, it encircles DNA and assists in coordinating the interactions and activities of various factors involved in DNA replication and repair. PCNA typically exists as a closed ring and does not load onto DNA readily. Clamp opening and its loading onto DNA is facilitated by the clamp loader complex, replication factor C (RFC), a hetero-pentameric AAA^+^ ATPase complex^51^. The RFC complex binds in a slightly tilted way on top of a closed PCNA ring (Fig. 5a) and ATP binding by the RFC subunits promotes disruption of one of the PCNA subunit interfaces and thereby opening of the PCNA ring. The PCNA:RFC:ATP complex binds specifically to primed DNA and recognition of the double-stranded/single-stranded junction stimulates ATP hydrolysis coupled to conformational changes in the RFC subunits. These changes, in turn, decrease the binding affinity of the RFC subunits for the outer surface of PCNA and lead to expulsion of the clamp loader and eventual reclosing of the clamp around DNA (Fig. 5b). The inner surface of the sliding clamp is lined with positively charged α-helices that mediate contacts with the negatively charged DNA phosphate backbone. Recent findings have shown that these positively charged residues are important for proper RFC binding in yeast systems. *Saccharomyces* *cerevisiae* PCNA variants with lysine/arginine residues mutated to alanine within this inner surface showed reduced RFC ATPase activity in the presence of DNA^52^. PCNA’s function and interactions are heavily regulated and fine tuned by PTMs, such as ubiquitylation, SUMOylation, methylation and acetylation^53^. Interestingly, proteomics studies have also found two succinylation sites within PCNA: one on the outer surface of the ring (K164), a position that is ubiquitylated upon DNA-damage and triggers recruitment of specialized translesion synthesis polymerases, and one position (K13) within the inner ring surface that is packed with positively charged residues and may be important for proper RFC-mediated DNA loading^6,37,46^.

To study the effects of these site-specific succinylations, we prepared PCNA-K13SucK and PCNA-K164SucK via genetic encoding of PrS-SucK and on-protein thioester hydrolysis in the presence of BME (Fig. 5c and Supplementary Fig. 11a). To validate that BME treatment and SucK incorporation did not impact PCNA folding and trimerization, we first examined the oligomeric state of the different PCNA variants in the presence and absence of BME via native polyacrylamide gel electrophoresis. BME treatment did not affect trimer formation and both PCNA-K13SucK and PCNA-K164SucK formed stable trimers in solution similar to WT PCNA, unlike a previously reported mutant (G178S)^54^ that was deficient in forming stable trimers and was used as control (Fig. 5d and Supplementary Fig. 11b).

To study the influence of succinylation on clamp loading, we adapted a previously reported fluorescence anisotropy assay to measure the affinity of RFC and PCNA variants for fluorescently labelled DNA. We expressed and purified hetero-pentameric human RFC (hRFC; Supplementary Fig. 12) and titrated it into a mixture of primer-template DNA with a 5′-unpaired overhang labelled with a tetramethyl-rhodamine fluorophore (TAMRA) and the different PCNA variants in the presence of the non-hydrolysable ATP analogue ATP-γ-S. For WT PCNA and the PCNA variant carrying the SucK modification at position 164 at the outer surface of the ring, we measured dissociation constants (*K*_D_) around 100 nM, in good agreement with previously determined values for WT PCNA^52^. Likewise, a PCNA variant bearing AcK at position 13 within the inner surface of the PCNA ring, a modification that was also found in proteomics data^6,37,46^, showed efficient binding to hRFC in our anisotropy assay (Supplementary Fig. 11). Strikingly, PCNA-K13SucK failed in showing any anisotropy difference upon incubation with TAMRA-labelled primer template DNA and titration of hRFC at different concentrations, indicating that succinylation within the inner surface of the PCNA ring severely impacts hRFC-mediated charging of PCNA onto DNA.

Succinylation could, thereby, play an important role in regulating the functions of PCNA in various cellular processes, including DNA replication, DNA repair and cell cycle control, as already shown for other PTMs^53^. Further studies on the succinylation state of K13 in PCNA in different cell cycle stages and upon DNA damage-causing cellular cues will be necessary to elucidate the functional role of PCNA-succinylation and possible impacts on genomic stability and potential involvement in disease states.

## Discussion

Despite the fact that thousands of succinylation and glutarylation sites have been mapped in bacteria, yeast and human cells^4–8^, functional and structural consequences of these PTMs remain heavily understudied, as tools to access homogeneously modified proteins in quantities required for biochemical and structural studies have been elusive. Herein, we report an approach for genetically encoding the acidic lysine acylations SucK and GluK, thereby providing a general and robust tool to study their impact on protein structure and function. To overcome problems inherent to negatively charged ncAAs, such as poor cellular uptake and low compatibility with the hydrophobic active site of PylRS, we synthesized neutral, thioester-masked SucK/GluK derivatives and identified the *Mb*PylRS variant—ThioRS—as specific synthetase for their incorporation into proteins. LC–MS analysis of purified proteins indicated that, depending on POI and position, the thioester functionalities were partially stable during protein expression and purification but could be hydrolysed quantitatively on-protein via mild chemical treatment, either at slightly elevated pH (pH 8.5–9) or at physiological pH in the presence of BME. Importantly, unlike many other ncAAs, the thioester-modified SucK and GluK derivatives can be accessed via easy and straightforward chemical transformations on solid support in multigram scale and, therefore, do not require advanced chemistry expertise, making our tool easily implementable in standard biochemistry laboratories.

We use our approach to site-specifically incorporate SucK or GluK into different bacterial and mammalian target proteins, including metabolic enzymes, signalling proteins and DNA-interacting proteins. We show how succinylation or glutarylation can modulate enzymatic activity of the glycolytic enzyme GAPDH, play a role in regulating protein–protein interactions in Ub-signalling events and influence DNA clamp loading and replication. We hereby expand the knowledge on the potential roles that succinylation or glutarylation may play in regulating and modulating biological processes. So far, the functional and structural impact of these PTMs on a molecular level focused on studying chromatin dynamics and nucleosome stability, as SucK/GluK-modified histone variants can be accessed chemically and nucleosomes can be reconstituted in vitro. Furthermore, cells and organisms with abolished or diminished activity of desuccinylases (for example, SIRT5) provided evidence of the regulatory importance of succinylation in metabolic processes^14,15^.

Proteomics data have shown substantial overlap between acetylation and succinylation, and many target proteins can be acetylated and succinylated at the same residues^6^. We now have the tools to study how these different PTMs targeting the same amino acids distinctly influence a protein’s fate. A recent study has, for example, shown that individually acetylated Ub variants show slight differences in their interactome, thereby linking the distinctly acetylated variants to specific cellular pathways^55^. Likewise, preparation of site-specific SucK-modified Ub and Ub-like proteins (Ubls) using our approach will allow systematic profiling of their impact on Ub/Ubl interacting proteins within the Ub/Ubl conjugation/deconjugation machinery and help in further dissecting the additional regulatory layer encoded in small-molecule PTMs attached to Ub/Ubls^42,43^. As proteins are prone to undergo a wide array of diverse modifications simultaneously at multiple sites, PTMs rarely exist in isolation and it is known that PTMs influence each other (PTM crosstalk)^56,57^. Importantly, SucK or GluK derivatives introduced here can also be incorporated using the *Ma*-derived variant *Ma*ThioRS, which has been shown to be orthogonal to *Mb*PylRS/tRNA variants^28,29^. This will, for example, enable the incorporation of SucK/GluK together with other PTMs such as AcK or phosphorylated amino acids by either suppressing two stop codons^58,59^ or making use of an *E. coli* strain with a compressed genetic code that allows sense codon reassignment^60^. Furthermore, also photocrosslinker or chemical crosslinker bearing ncAAs could be incorporated together with SucK/GluK to identify new protein interactors of SucK/GluK-modified POIs in cell lysates or to chemically stabilize transient POI–reader complexes^61–64^.

Importantly we show that genetically installed SucK/GluK modifications can be recognized by endogenous deacylases, such as SIRT5 and CobB, and our approach will enable the profiling of activities and specificities of various deacylases in the context of folded target proteins with site-specific SucK/GluK modifications. This could aid endeavours in developing selective inhibitors for sirtuins that are specific for desuccinylation or deglutarylation^65^.

In its current form, our approach allows incorporation of SucK/GluK at any position in any protein heterologously expressed in *E. coli*, as long as the corresponding site can be amber suppressed, but to effectively hydrolyse the thioester linkage, the target proteins have to withstand incubation at slightly elevated pH or incubation in the presence of BME/TCEP. These conditions were compatible with all tested proteins, including multimeric proteins bearing multiple cysteines, such as PCNA and GAPDH, making our approach widely applicable. For more sensitive proteins, thioester hydrolysis conditions might have to be optimized on a case-by-case basis.

To study effects of succinylation and glutarylation in living cells, future work will concentrate on the development and site-specific incorporation of photocaged SucK/GluK derivatives, allowing instalment of these acidic lysine modifications with temporal/spatial resolution. Ideally, such approaches should be combined with SucK/GluK mimics displaying amide bond or carboxylic acid isosteres, such as thioamides or tetrazoles^25,65,66^, which are refractory to hydrolysis by sirtuins, enabling the investigation of stable succinylation/glutarylation in physiological settings.

With this genetic code expansion technology, we demonstrate the site-specific on-demand modification of various complex target proteins with SucK and GluK, which allows us to mechanistically address how these PTMs that reverse the charge of an amino acid from +1 to −1 impact fundamental biological processes and pathways.

## Methods

### Expression and purification of SucK/GluK/AcK-bearing H6-tagged Ub/sfGFP/PCNA/AzoR/GAPDH

Chemically competent *E. coli* K12 was transformed with plasmids containing *Mb* or *Ma*ThioRS, corresponding tRNA (*Mb* or *Ma*PylT) and the H6-tagged POI bearing a TAG codon (see Supplementary Table 3). After recovery with 1 ml of SOC medium for 1 h at 37 °C, the cells were cultured overnight in 50 ml of 2× YT medium containing the corresponding antibiotics using the following concentrations: tetracycline 17.5 µg ml^−1^, ampicillin 100 µg ml^−1^ and chloramphenicol 50 µg ml^−1^, at 37 °C and 200 rpm. The overnight culture was diluted to an optical density at 600 nm (OD_600_) of 0.05 in 200 ml of fresh 2× YT medium supplemented with the corresponding antibiotics (tetracycline 8.75 µg ml^−1^, ampicillin 50 µg ml^−1^ and chloramphenicol 25 µg ml^−1^) and PrS-GluK (1 mM), PrS-SucK (4 mM) or AcK (10 mM) and cultured at 37 °C, 200 rpm until OD_600_ of 0.8–1.0 was reached. Arabinose was added to a final concentration of 0.02 % (w/v), and protein expression was induced for 16 h at 37 °C. The cells were collected by centrifugation (4,000*g* for 20 min at 4 °C), and Ni-NTA affinity purification was performed as for the WT proteins (see Supplementary Information). After purification via Ni-NTA slurry, the fractions containing the protein were pooled, concentrated and rebuffered (50 mM Tris buffer pH 7.5 at 4 °C, 150 mM NaCl) using Amicon centrifugal filter units with a 3 kDa/10 kDa/30 kDa molecular weight cutoff (MWCO; Millipore).

To hydrolyse the thioester on the ncAA (non-canonical amino acid), the protein was rebuffered to 50 mM Tris pH 7.5 at 4 °C, 150 mM NaCl and 100 mM BME and incubated at 37 °C for 4–16 h or using slightly different, optimized conditions. The thioester hydrolysis progress was monitored via LC–MS. As soon as the thioester was hydrolysed completely, the protein was rebuffered (50 mM Tris pH 7.5 at 4 °C and 150 mM NaCl) using Amicon centrifugal filter units with a 3 kDa/10 kDa/30 kDa MWCO (Millipore) and further purified via size-exclusion chromatography (SEC) using a Superdex Increase 75 10/300 (GE Healthcare) with a buffer containing 25 mM Tris pH 7.5 at 4 °C and 150 mM NaCl. The fractions containing the pure proteins were pooled together and concentrated using Amicon centrifugal filters. The purified proteins were analysed by 15% sodium dodecyl sulfate–polyacrylamide gel electrophoresis (SDS–PAGE) and MS. The protein concentration was calculated from the measured A280 absorption, and the extinction coefficients were calculated with ProtParam^69^. In case of Ub, determination of the protein concentration using the absorption at 280 nm is inaccurate (owing to the low extinction coefficient, *ε*), and therefore bicinchoninic acid (BCA; Thermo Scientific) and Bradford (Sigma-Aldrich) assays were used for accurate protein concentration determination, or the concentration of purified Ub was adjusted densitometrically. The purified proteins were analysed by 15% SDS–PAGE and MS, flash frozen using liquid nitrogen and stored at −80 °C until further use.

The yields were determined via measuring absorption at 280 nm, or in the case of Ub and Ub variants via BCA (Thermo Scientific) and Bradford (Sigma-Aldrich) assays. For determination of the concentrations and yields of sfGFP and sfGFP variants, the GFP-fluorescence (excitation 485 nm and emission 535 nm) was measured, and a calibration curve was used to determine the protein concentration (Supplementary Table 4 and Supplementary Fig. 2).

### Thioester hydrolysis conditions for different POIs

For sfGFP, the conditions included lysis buffer consisting of 100 mM 4-(2-hydroxyethyl)-1-piperazineethanesulfonic acid (HEPES) pH 8.0, 150 mM NaCl, 30 mM imidazole, 0.175 mg ml^−1^ phenylmethylsulfonyl fluoride (PMSF), 0.1 mg ml^−1^ DNase I and one cOmplete protease inhibitor tablet (Roche) and wash buffer consisting of 100 mM HEPES pH 8.0, 150 mM NaCl and 30 mM imidazole. The thioester hydrolysis was performed in 100 mM HEPES pH 8.0, 150 mM NaCl and 100 mM BME at 37 °C for 4 h.

The conditions for AzoR included lysis buffer consisting of 50 mM Tris pH 7.5, 150 mM NaCl, 30 mM imidazole, 0.175 mg ml^−1^ PMSF, 0.1 mg ml^−1^ DNase I and one cOmplete protease inhibitor tablet (Roche) and wash buffer consisting of 50 mM Tris pH 7.5, 150 mM NaCl and 30 mM imidazole. The thioester hydrolysis was performed in 50 mM Tris pH 7.5, 150 mM NaCl and 100 mM BME at 37 °C for 8 h.

The conditions for GAPDH inlcuded lysis buffer consisting of 50 mM Tris pH 7.5, 150 mM NaCl, 30 mM imidazole, 0.175 mg ml^−1^ PMSF, 0.1 mg mL^−1^ DNase I and one cOmplete protease inhibitor tablet (Roche) and wash buffer consisting of 50 mM Tris pH 7.5, 150 mM NaCl and 30 mM imidazole. The thioester hydrolysis was performed in 25 mM Tris pH 7.5 at RT, 150 mM NaCl and 100 BME at 37 °C for 16 h or pH 9.0 and 100 mM *N*-cyclohexyl-2-aminoethanesulfonic acid (CHES) pH 9.0, 150 mM NaCl at r.t. for 48 h.

The conditions for Ub included lysis buffer consisting of 30 mM MES pH 6.0, 150 mM NaCl, 30 mM imidazole, 0.175 mg ml^−1^ PMSF, 0.1 mg ml^−1^ DNase I and one cOmplete protease inhibitor tablet (Roche) and wash buffer consisting of 30 mM MES pH 6.0, 150 mM NaCl and 30 mM imidazole. The thioester hydrolysis was performed in 30 mM MES pH 6.0, 150 mM NaCl and 300 mM BME at 37 °C for 3–4 h.

The conditions for PCNA inlcuded lysis buffer consisting of 50 mM Tris pH 7.5, 150 mM NaCl, 30 mM imidazole, 0.175 mg ml^−1^ PMSF, 0.1 mg ml^−1^ DNase I and one cOmplete protease inhibitor tablet (Roche) and wash buffer consisting of 50 mM Tris pH 7.5, 150 mM NaCl and 30 mM imidazole. The thioester hydrolysis was performed in 50 mM Tris pH 7.5, 150 mM NaCl and 100 mM BME at 37 °C for 16 h.

### Expression and purification of SucK/GluK-bearing histone H3 variants

Chemically competent *E. coli* DH10β was transformed with H6-tagged histone H3 with a TAG codon at K122 in a pBAD plasmid and the ThioRS and PylT in a pEVOL plasmid (Supplementary Table 3). After recovery with 1 ml of SOC medium for 1 h at 37 °C, the cells were cultured overnight in 50 ml of 2× YT medium containing chloramphenicol (50 µg ml^−1^) and ampicillin (100 µg ml^−1^) at 37 °C and 200 rpm. The overnight culture was diluted to an OD_600_ of 0.05 in 200 ml fresh 2× YT medium supplemented with chloramphenicol (25 µg ml^−1^) and ampicillin (50 µg ml^−1^), 20 mM NAM and the ncAAs PrS-GluK (1 mM) or PrS-SucK (4 mM) and cultured at 37 °C at 200 rpm, until the OD_600_ reached 0.8–1.0. Arabinose was added to a final concentration of 0.2% (w/v), and the protein expression was induced for 16 h at 37 °C. The cells were collected by centrifugation (4,000*g* for 20 min at 4 °C), and Ni-NTA affinity purification was performed as for the WT histone H3. After purification via Ni-NTA slurry, the histones bearing PrS-GluK or PrS-SucK were rebuffered to 50 mM HEPES pH 7.0, 500 mM NaCl and 6 M urea supplemented with 100 mM BME and incubated at 37 °C for 5–7 h. The thioester hydrolysis progress was monitored via LC–MS. As soon as the thioester was hydrolysed completely, the histone variants were rebuffered using Amicon centrifugal filter units (Millipore) with a 3 kDa MWCO to 50 mM HEPES pH 7.0, 100 mM NaCl, 6 M urea and 1 mM dithiothreitol (DTT) and subjected to cation exchange chromatography using a Resource S column (Cytiva) with a gradient from 5% to 40% B (buffer A: 50 mM HEPES pH 7.0, 100 mM NaCl, 6 M urea and 1 mM DTT; buffer B: 50 mM HEPES pH 7.0, 1 M NaCl, 6 M urea and 1 mM DTT). Fractions containing H3 (identified via SDS–PAGE) were pooled and refolded by stepwise dialysis (from 6 M urea to 0 M urea in six steps) using Pur-A-Lyzer (Sigma-Aldrich) dialysis chambers with a 3.5 kDa cutoff at 4 °C into histone storage buffer (50 mM Tris pH 7.5, 150 mM NaCl and 0.5 mM TCEP). Finally, the histones were concentrated using Amicon centrifugal filter units (Millipore) with a 3 kDa MWCO. The protein concentration was calculated from the measured A280 absorption (extinction coefficients were calculated with ProtParam^69^). Histone H3 was flash frozen in liquid nitrogen and stored at −80 °C until further use.

### Deacylation assays

SucK/GluK-bearing histone H3 variants were diluted to 7.5 µM in deacylation assay buffer (50 mM Tris pH 8.0, 100 mM NaCl, 5 mM MgCl_2_, 1 mM DTT and 5 mM NAD^+^), SIRT5 was added at 5 µM and the mixture was incubated at 37 °C. The samples after 0, 3 and 6 h were diluted 1:1 in 1% formic acid in water for electrospray ionization quadrupole time-of-flight MS analysis or analysed via denaturing SDS–PAGE and western blot using the following antibodies: anti-SucK (mouse IgG, PTM Biolabs and PTM-419; dilution 1:1,000), anti-GluK (mouse IgG, PTM Biolabs and PTM-1152; dilution 1:1,000) and anti-mouse IgG peroxidase-coupled (Sigma-Aldrich, A4416; dilution 1:10,000).

SucK/GluK-bearing AzoR variants were diluted to 7.5 µM in deacylation assay buffer (50 mM Tris pH 8.0, 100 mM NaCl, 5 mM MgCl_2_, 1 mM DTT and 5 mM NAD^+^), CobB or SIRT5 was added at 5 µM and the mixture was incubated at 37 °C. Samples after 0, 3 and 6 h were diluted 1:1 in 1% formic acid in water for electrospray ionization quadrupole time-of-flight MS analysis or analysed via denaturing SDS–PAGE and western blot using the following antibodies: anti-SucK (mouse IgG, PTM Biolabs and PTM-419; dilution 1:1,000) and anti-mouse IgG peroxidase-coupled (Sigma-Aldrich, A4416; dilution 1:10,000).

### GAPDH assays

GAPDH variants were diluted to 2 nM in GAPDH assay buffer (20 mM Tris pH 8.0, 50 mM NaCl, 2.5 mM EDTA and 2 mM NAD^+^) and increasing concentrations of d-GAP (0–1.5 mM). The reaction was started by addition of 15 mM sodium arsenate, and the GAPDH activity was monitored spectrophotometrically by measuring the NADH fluorescence at 450 nm over 15 min (340 nm for excitation) on a Tecan SPARK microplate reader (Tecan) at 25 °C in a total volume of 200 µl. Each condition was measured in triplicates from distinct experiments.

To determine the enzyme kinetics, the fluorescence intensity was plotted against the reaction time. The slope of the initial linear part of the reaction (*v*_0_) was determined for the first 100 s of the reaction. The signal intensity of the NADH fluorescence given in relative fluorescence units by the instrument was referenced to the NADH concentration by measuring a calibration curve with set NADH concentrations.

*v*_0_ in units per mg of enzyme (U mg^−1^ (was plotted against the d-GAP concentration [S], and *V*_max_ and *K*_m_ were calculated with a Michaelis–Menten fit (GraphPad Prism version 10) corresponding to the following equation:

$$v=\frac{{v}_{\max }* \left[{\mathrm{S}}\right]}{{K}_{\mathrm{m}}+\left[{\mathrm{S}}\right]}.$$

### Preparation of natively linked K11-diUb variants

Assembly of K11-linked diUbs was carried out as previously described with slight adjustments^49,70^. In short, the assembly reactions contained 100 nM H6-UBE1 (Boston Biochem, cat. no. E-304-050), 5 μM UBE2S, 150 µM tagless Ub WT and 150 µM Ub WT-H6 or H6-tagged Ub variants bearing SucK or AcK at K33. Reactions were incubated at 37 °C for typically 16 h in diUb reaction buffer (50 mM Tris pH 7.5, 10 mM MgCl_2_, 0.6 mM DTT and 10 mM ATP). After incubation, the samples were diluted in wash buffer to reduce the overall DTT concentration, and 250 µl (per ml reaction volume) of Ni-NTA slurry (Jena Bioscience, equilibrated with wash buffer (50 mM Tris pH 7.5, 150 mM NaCl and 30 mM imidazole)) was added to the reaction, and the mixture was incubated agitating for 1 h at 4 °C. After incubation, the mixture was transferred to an empty plastic column and washed with 40 column volumes of wash buffer to remove UBE2S and unreacted Ub WT. H6-tagged proteins were eluted in 0.2 ml fractions with wash buffer supplemented with 300 mM imidazole pH 8.0. The fractions containing the mixture of desired K11-linked diUb and remaining H6-tagged monoUbs were pooled and concentrated using Amicon centrifugal filter units with a 10 kDa MWCO (Millipore). To remove remaining H6-tagged monoUbs, SEC was performed using a Superdex Increase 75 10/300 (GE Healthcare) with SEC buffer (50 mM Tris pH 7.5 and 150 mM NaCl). Fractions containing the desired K11-linked diUbs were pooled and concentrated using Amicon centrifugal filter units with a 3 kDa MWCO (Millipore). The protein concentration was determined using BCA (Thermo Scientific) and Bradford (Sigma-Aldrich) assays. Purified DiUbs were flash frozen using liquid nitrogen and stored at −80 °C until further use.

### DUB assays

DiUbs were diluted into DUB reaction buffer (50 mM HEPES pH 7.4, 150 mM NaCl, 2 mM DTT and 2 mM TCEP) to 5 µM, Cezanne was added at 7.5 nM, and the mixture was incubated at 4 °C. At denoted time points, 3.5 μl samples were taken and quenched by the addition of 4× SDS loading buffer. After boiling at 95 °C for 10 min, the samples were loaded on SDS–PAGE and visualized by Coomassie staining.

### *K*_D_ determination via fluorescence anisotropy

DNA oligos 1 and 2 (Supplementary Table 2) were annealed by heating the oligonucleotides to 95 °C and gradually cooling down to r.t.^71^. The fluorescence anisotropy experiments were conducted on a Jasco FP-8500 fluorescence spectrometer equipped with polarizers (Jasco). Excitation and emission monochromators were set to 546 nm and 576 nm, and measurements were performed at r.t. with a bandwidth of 5 nm. The fluorescence anisotropy assay was adapted from literature^52^. For fluorescence anisotropy measurements, labelled DNA and PCNA were mixed in the assay buffer (30 mM Tris pH 7.5, 200 mM NaCl. 10 mM MgCl_2_, 2 mM DTT, 1 mM ATP-γ-S and 5% glycerol). The concentration of 5-carboxytetramethylrhodamin (5′-TAMRA)-labelled DNA was held constant at 100 nM, and the concentration of PCNA at 1 µM for all measurements. To enable the loading of PCNA onto DNA, hRFC was added in different concentrations to 5′-TAMRA-labelled, primed DNA and PCNA, and the anisotropy was measured for each concentration in triplicates from distinct samples. All data processing was performed using GraphPad Prism 10 (GraphPad software). The *K*_D_ was determined using a single-binding-site model, and the average values and error bars (standard deviation) were calculated from three different experiments (*n* = 3).

### Native PAGE

Native gel electrophoresis was performed for PCNA WT (untreated and BME treated) as well as PCNA-G178S and PCNA-K13SucK/PCNA-K164SucK as described previously^54^. PCNA WT and PCNA variants were diluted to 0.05 mg ml^−1^ and 0.5 mg ml^−1^ and incubated in 10 mM Tris, pH 7.6, 150 mM NaCl, 2.5% Ficoll-400, 10 mM EDTA, 0.03% xylene cyanol and 0.03% Orange G for 10 min at 4 °C. The samples were run on a precast 4–20% gradient, non-denaturing Tris-glycine gel (BioRad) with 150 V (10–25 mA) at 4 °C and Novex Tris-glycine native running buffer (1×, Thermo Fischer Scientific). The proteins were visualized by Coomassie staining.

### Reporting summary

Further information on research design is available in the Nature Portfolio Reporting Summary linked to this article.

## Online content

Any methods, additional references, Nature Portfolio reporting summaries, source data, extended data, supplementary information, acknowledgements, peer review information; details of author contributions and competing interests; and statements of data and code availability are available at 10.1038/s41557-024-01500-5.

### Supplementary information

Supplementary Information Supplementary methods, information, figures, tables and sequences.

Reporting Summary

### Source data

Source Data Fig. 1 Full gel in Fig. 1c (unprocessed and fully uncropped) and LC–MS data in Fig. 1d.

Source Data Fig. 2 Full gels in Figs. 2b,d (unprocessed and fully uncropped) and LC–MS data in Fig. 2c.

Source Data Fig. 3 Full gels in Fig. 3b (unprocessed and fully uncropped), LC–MS data in Fig. 3c and raw data GAPDH assay in Fig. 3d.

Source Data Fig. 4 LC–MS data in Fig. 4c and full gels in Fig. 4d (unprocessed and fully uncropped).

Source Data Fig. 5 Full gels in Figs. 5c,d (unprocessed and fully uncropped) and raw data fluorescence anisotropy assay in Fig. 5e.

## Data Availability

The data generated or analysed during this study are included in this article or its supplementary information files. The protein structures and models used for the figures are available under accession codes 1kx5, 1znq, 5lrv, 1sxj and 6gis. Source data are provided with this paper.
