# Supplementary material for: Deciphering functional roles of protein succinylation and glutarylation using genetic code expansion
Source: Nat Chem. 2024 Mar 26;16(6):913–21. doi: 10.1038/s41557-024-01500-5 (PMC11164685; doi:10.1038/s41557-024-01500-5)
Supplement: Supplementary file 1 — Supplementary methods, information, figures, tables and sequences. [file 41557_2024_1500_MOESM1_ESM.pdf]

nature chemistry

Article https://doi.org/10.1038/s41557-024-01500-5

Deciphering functional roles of protein succinylation and glutarylation using genetic code expansion

In the format provided by the authors and unedited

Table of Contents:

Supplementary Figures S1-S18 2
Experimental procedures 20
1. General methods: Plasmids and reagents 20
1.1 Primers 20
1.2 Oligonucleotides for fluorescence anisotropy 22
1.3 Plasmids 22
1.4 Amino acid sequences of proteins 24
2. Chemical synthesis 27
3. 96-well based PylRS screen 29
4. Protein expression and purification 29
4.1 Expression and purification of tagless Ub 29
4.2 Expression and purification of H6-tagged wt Ub/sfGFP/PCNA/AzoR/GAPDH and respective mutants . 30
4.3 Expression and purification of wt Histone H3 30
4.4 Expression and purification of hRFC 31
4.5 Expression and purification of UBE2S 32
4.6 Expression and purification of Cezanne 33
4.7 Expression and purification of CobB 33
4.8 Expression and purification of SIRT5 34
4.9 Protein purification yields 34
5. LC-MS and NMR 36
Supplementary References 37
Fully uncropped and unprocessed gels 38

Supplementary Figures S1-S18

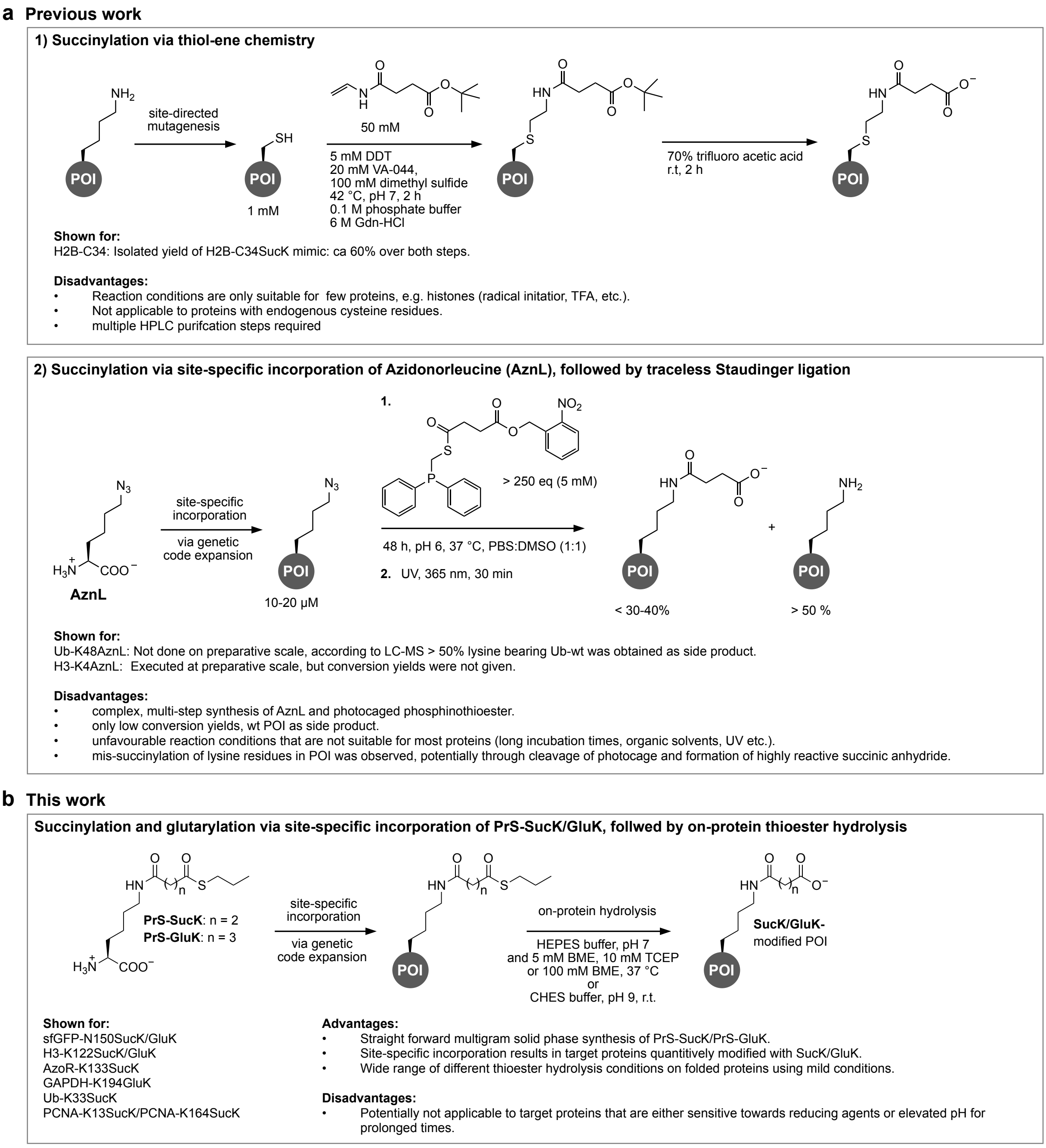

Supplementary Figure S1: Methods for site-specific installation of succinyl lysine (SucK) and glutaryl lysine (GluK). a) Previous methods for SucK installation. Site directed mutagenesis followed by thiol-ene click and subsequent deprotection (top)[1]. Site-specific incorporation of azidonorleucine followed by Staudinger ligation and photodeprotection (bottom)[2]. b) Technology developed in this manuscript. Site-specific installation of SucK and GluK via masked thioester derivatives followed by on-protein thioester hydrolysis.

a

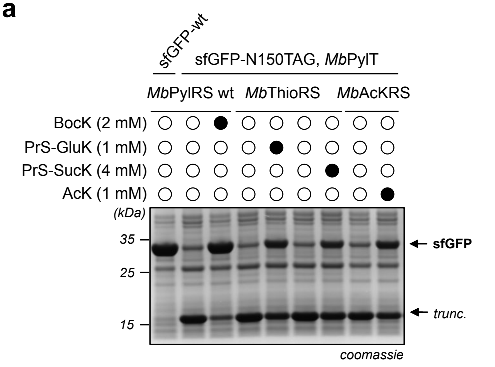

b

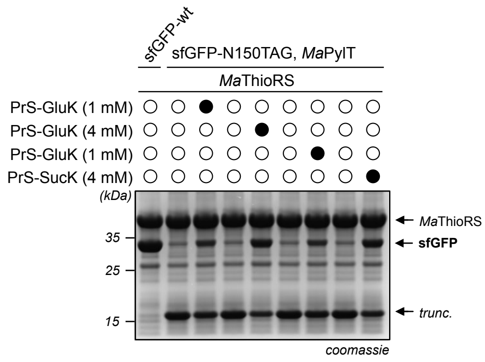

c

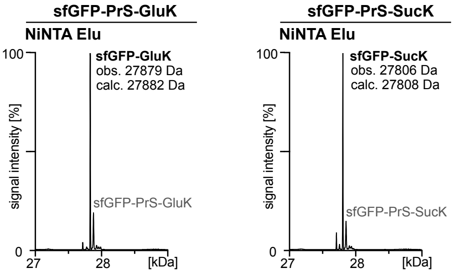

d

sfGFP variant	PylRS	ncAA added	ncAA conc. added	yield (mg/L)
wt	*Mb*	-	-	139 ± 7
N150TAG	*Mb*	BocK	2 mM	101 ± 9
N150TAG	*Mb*	PrS-GluK	1 mM	37 ± 3
N150TAG	*Mb*	PrS-SucK	4 mM	26 ± 2
N150TAG	*Mb*	AcK	1 mM	33 ± 1
N150TAG	*Ma*	PrS-GluK	4 mM	51 ± 8
N150TAG	*Ma*	PrS-SucK	4 mM	64 ± 4

Supplementary Figure S2: Incorporation of PrS-GluK and PrS-SucK into sfGFP via genetic code expansion and comparison between *Mb* and *Ma* PylRS/tRNA pairs. a) SDS-PAGE analysis of the expression of sfGFP-N150PrS-GluK/SucK in comparison to sfGFP-wt as well as sfGFP-N150AcK. b) Incorporation of PrS-GluK and PrS-SucK via the *Ma* PylRS/tRNA pair. SDS-PAGE analysis of the expression of sfGFP-N150PrS-GluK/SucK in comparison to sfGFP-wt. c) LC-MS analysis of sfGFP-N150PrS-GluK/SucK after Ni-NTA-affinity purification. sfGFP was expressed using the *Ma*ThioRS/tRNA pair using 4 mM of the respective ncAAs. d) Quantification of sfGFP yields. Yields were determined by measuring in-lysate GFP fluorescence. Consistent results were obtained over three biologically independent replicate experiments. Full gels can be found in Supplementary Figure S14.

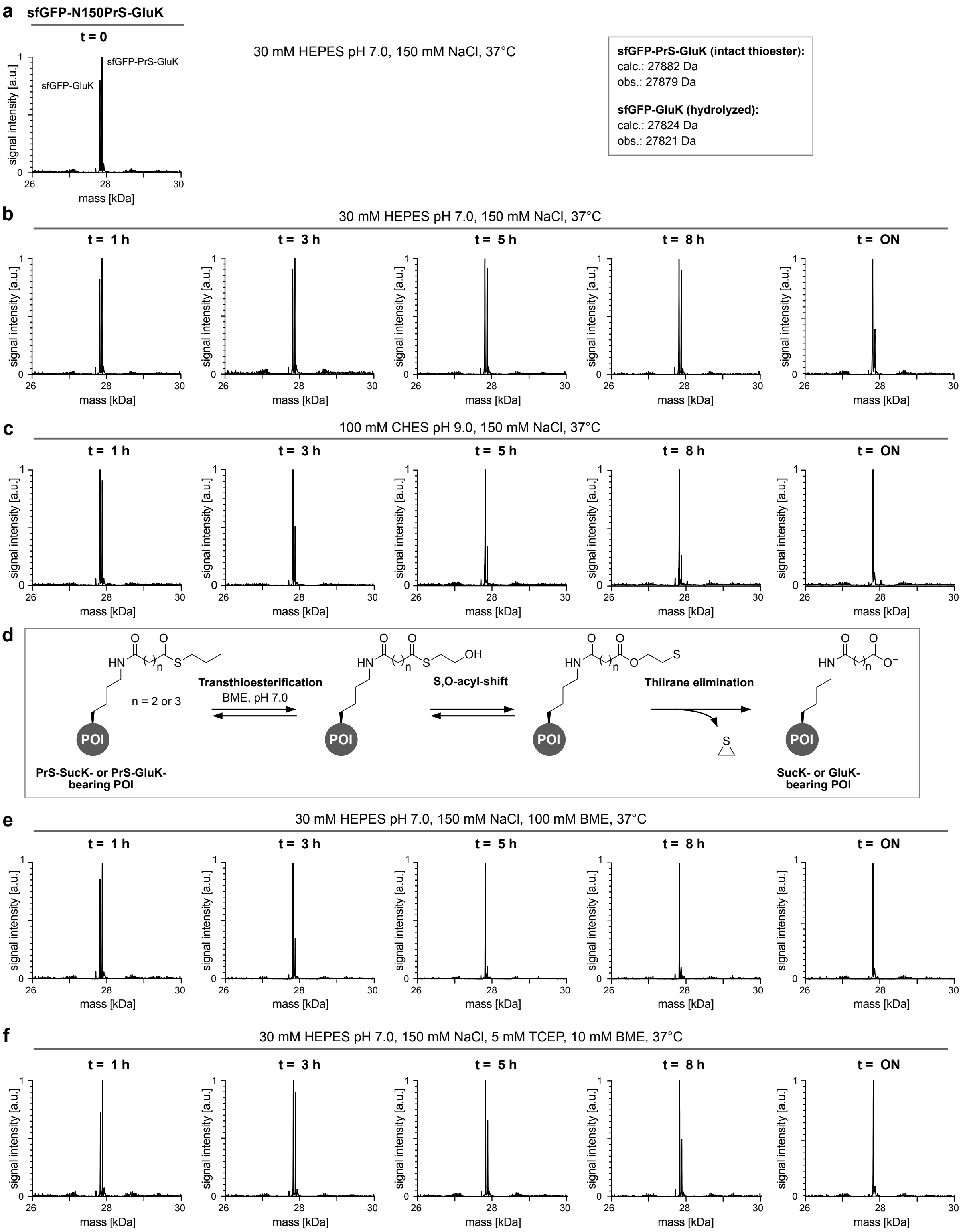

Supplementary Figure S3: Screening of on-protein thioester hydrolysis conditions. a) LC-MS analysis of sfGFP-N150PrS-GluK after Ni-NTA purification. b) LC-MS analysis of spontaneous thioester hydrolysis in 30 mM HEPES pH 7.0, 150 mM NaCl, 37 °C (samples were taken after denoted time points). c) LC-MS analysis of thioester hydrolysis under alkaline conditions: in 100 mM CHES pH 9.0, 150 mM NaCl, 37 °C. d) Schematic representation of BME-mediated thioester hydrolysis mechanism. e) LC-MS analysis of BME-mediated thioester hydrolysis in 30 mM HEPES pH 7.0, 100 mM BME,150 mM NaCl, 37 °C. f) LC-MS analysis of thioester hydrolysis in the presence of BME and TCEP in 30 mM HEPES pH 7.0, 5 mM TCEP, 10 mM BME, 150 mM NaCl, 37 °C. Consistent results were obtained over three biologically independent replicate experiments.

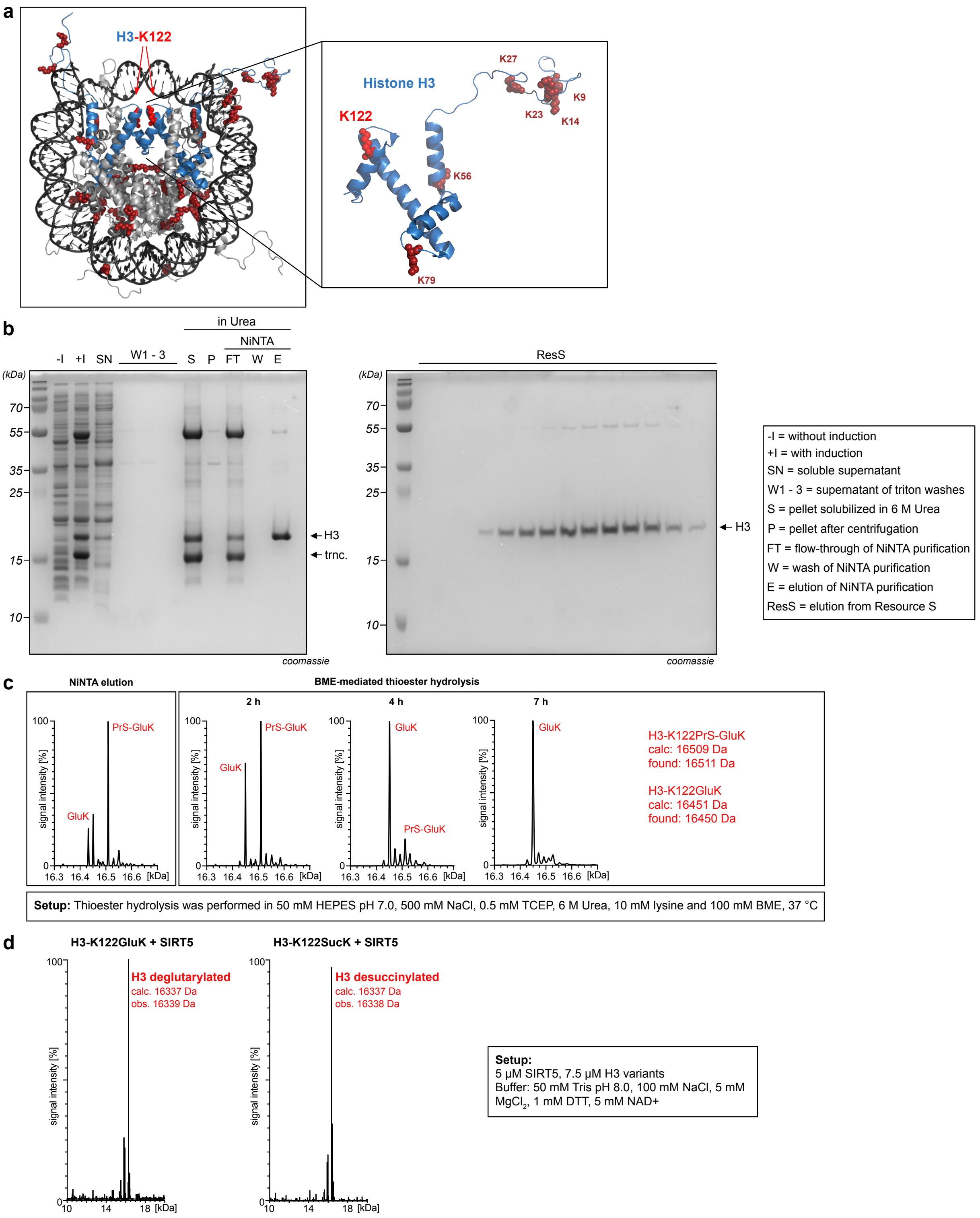

Supplementary Figure S4: Generation of glutarylated and succinylated histone H3 and deacylation via SIRT5. a) Structure of the nucleosome core particle and histone H3 (blue) (PDB: 1kx5)[3]. Lysine residues that are known to be succinylated or glutarylated are shown in red. b) SDS-PAGE analysis of Ni-NTA purification and size exclusion column chromatography (Superdex 75 10/300) of H3-K122GluK. c) LC-MS analysis of H3-K122PrS-GluK after Ni-NTA purification and a time course of BME-mediated on-protein thioester hydrolysis. d) LC-MS analysis of H3-K122GluK and H3-K122SucK after treatment with SIRT5. Succinylation and glutarylation could be reverted quantitatively. Consistent results were obtained over three biologically independent replicate experiments. Full gels can be found in Supplementary Figure S13; non-deconvoluted m/z spectra can be found in Supplementary Figure S16.

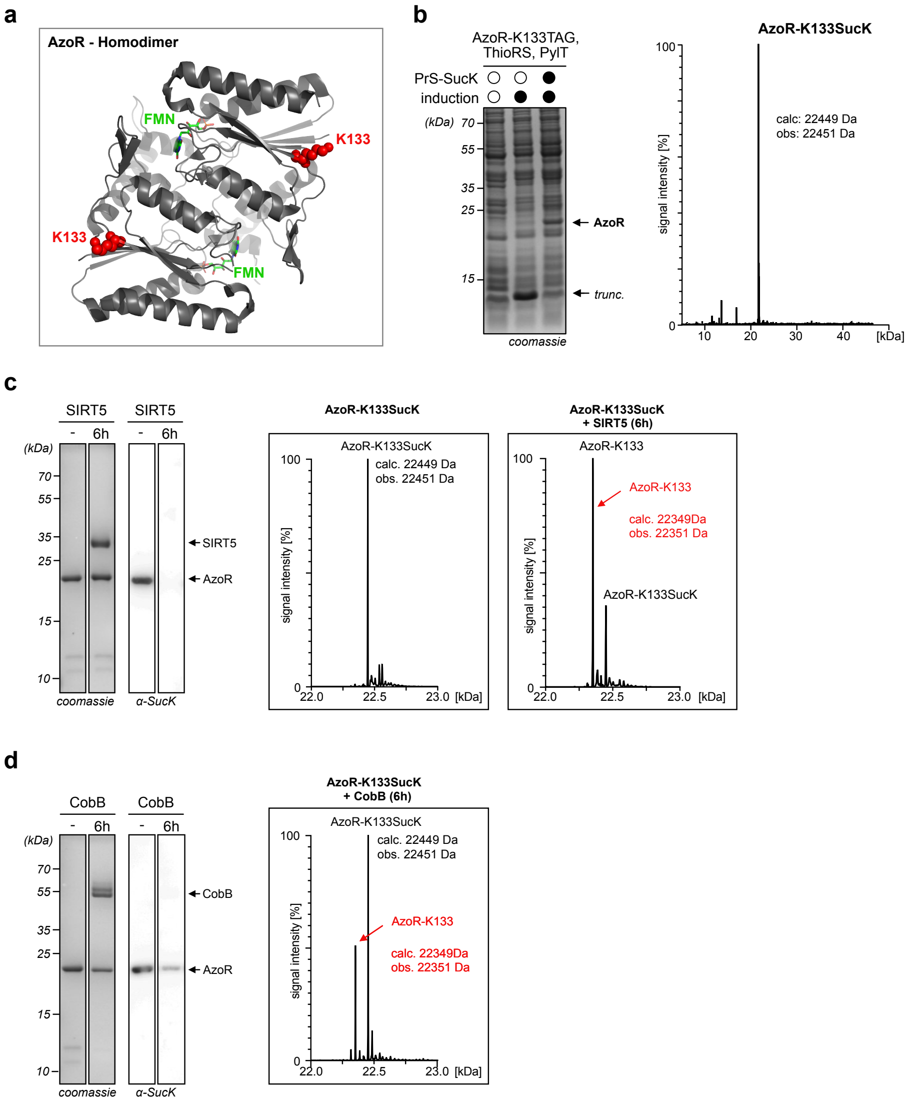

Supplementary Figure S5: Generation of succinylated AzoR and deacylation assays. a) Structure of the AzoR homodimer (PDB: 2z9d)[4]. The succinylated lysine residues are highlighted in red. The cofactor FMN is shown in green. b) Left: SDS-PAGE analysis of the expression of AzoR-K133PrS-SucK. Right: LC-MS analysis of purified AzoR-K133SucK. c) SDS-PAGE, Western blot, and LC-MS analysis of SIRT5-mediated deacylation of AzoR-K133SucK. d) SDS-PAGE, Western blot, and LC-MS analysis of CobB-mediated deacylation of AzoR-K133SucK. Full gels can be found in Supplementary Figure S13. Consistent results were obtained over three biologically independent replicate experiments.

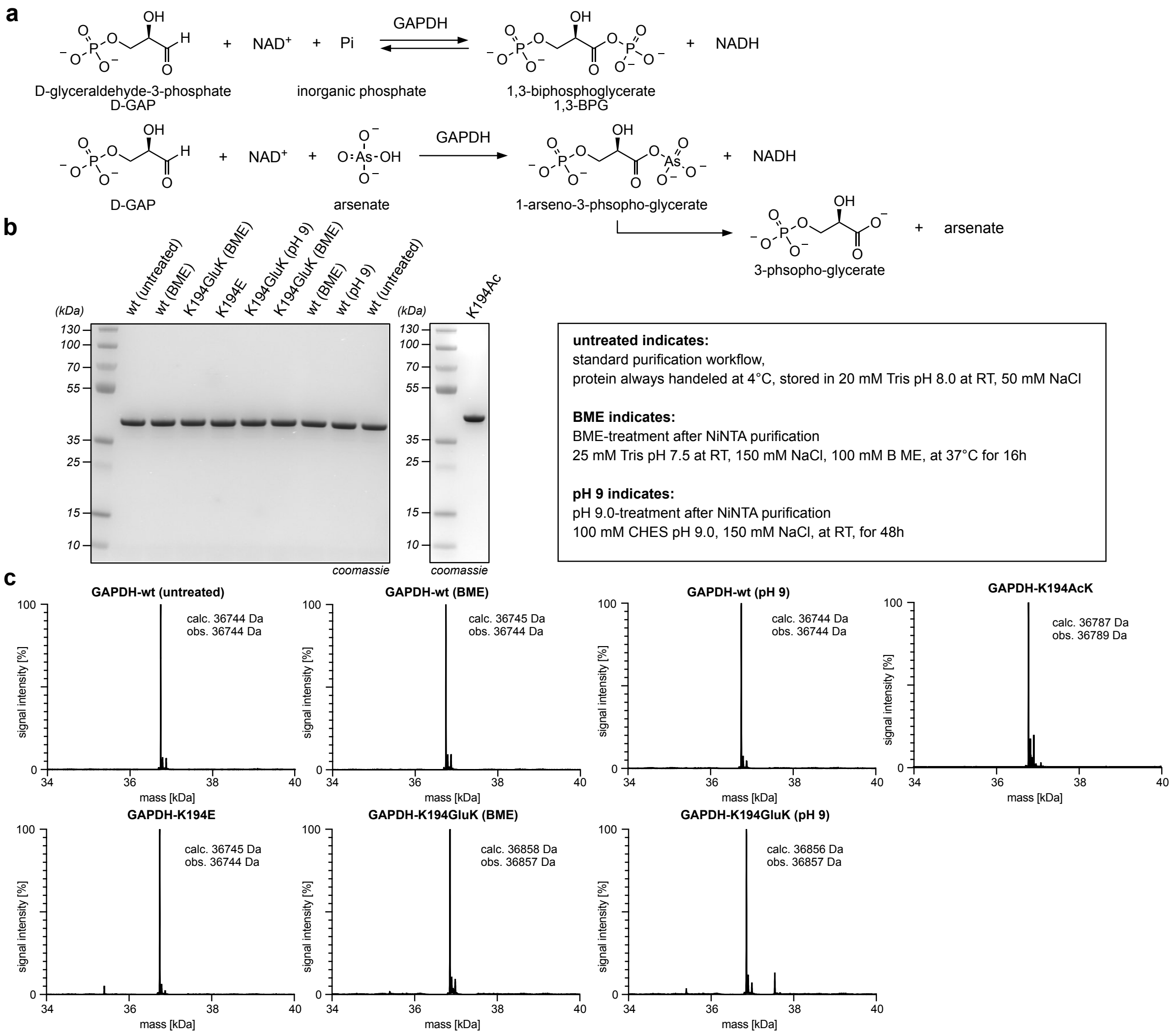

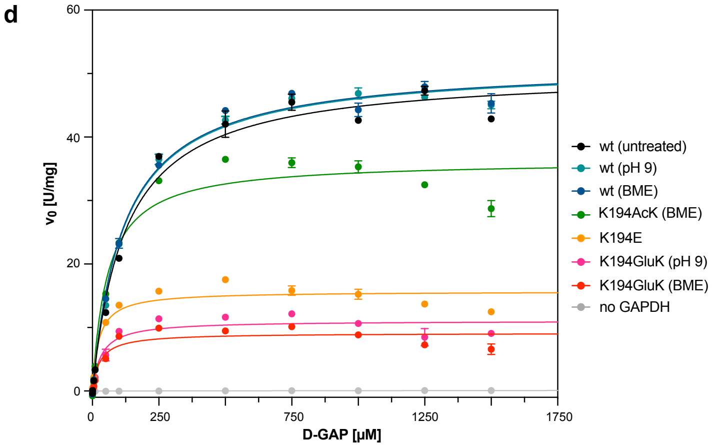

GAPDH	Km [µM]	vmax [U/mg]
wt (untreated)	123 ± 10	50.4 ± 0.9
wt (pH 9)	118 ± 6	51.5 ± 0.6
wt (BME)	117 ± 7	51.6 ± 0.6
K194AcK (BME)	56 ± 8	36.3 ± 0.9
K194E	24 ± 4	15.7 ± 0.4
K194GluK (pH 9)	33 ± 7	11.1 ± 0.4
K194GluK (BME)	28 ± 7	9.1 ± 0.4

Supplementary Figure S6: Glutarylation of GAPDH at K194 regulates its enzymatic activity. a) Schematic representation of the GAPDH-catalyzed conversion of D-GAP to 1,3-BPG (top). Arsenate was used as a phosphate mimic in the GAPDH-catalyzed irreversible conversion of D-GAP to 1-arseno-3-phospho-glycerate, which decomposes to arsenate and 3-phospho-glycerate (bottom). b) SDS-PAGE analysis of purified wt GAPDH and GAPDH variants after treatment with either BME or incubation under alkaline conditions. Consistent results were obtained over three distinct replicate experiments. Full gels are shown in Supplementary Figure S14. c) LC-MS analysis confirmed the integrity of purified wt GAPDH and GAPDH variants. Non-deconvoluted m/z spectra are shown in Supplementary Figure S16. d) Analysis of the enzymatic activity of GAPDH wt and GAPDH variants including controls for the on-protein thioester hydrolysis conditions (100 mM BME or pH 9.0). The initial reaction velocity (v_0) was plotted against the D-GAP concentration and fitted with a Michaelis-Menten model to determine K_m and v_{max} values. Average values and errors (SEM) were calculated from three biologically independent experiments (n=3). All data processing was performed using GraphPad Prism 10 (GraphPad software).

GAPDH-wt (untreated)

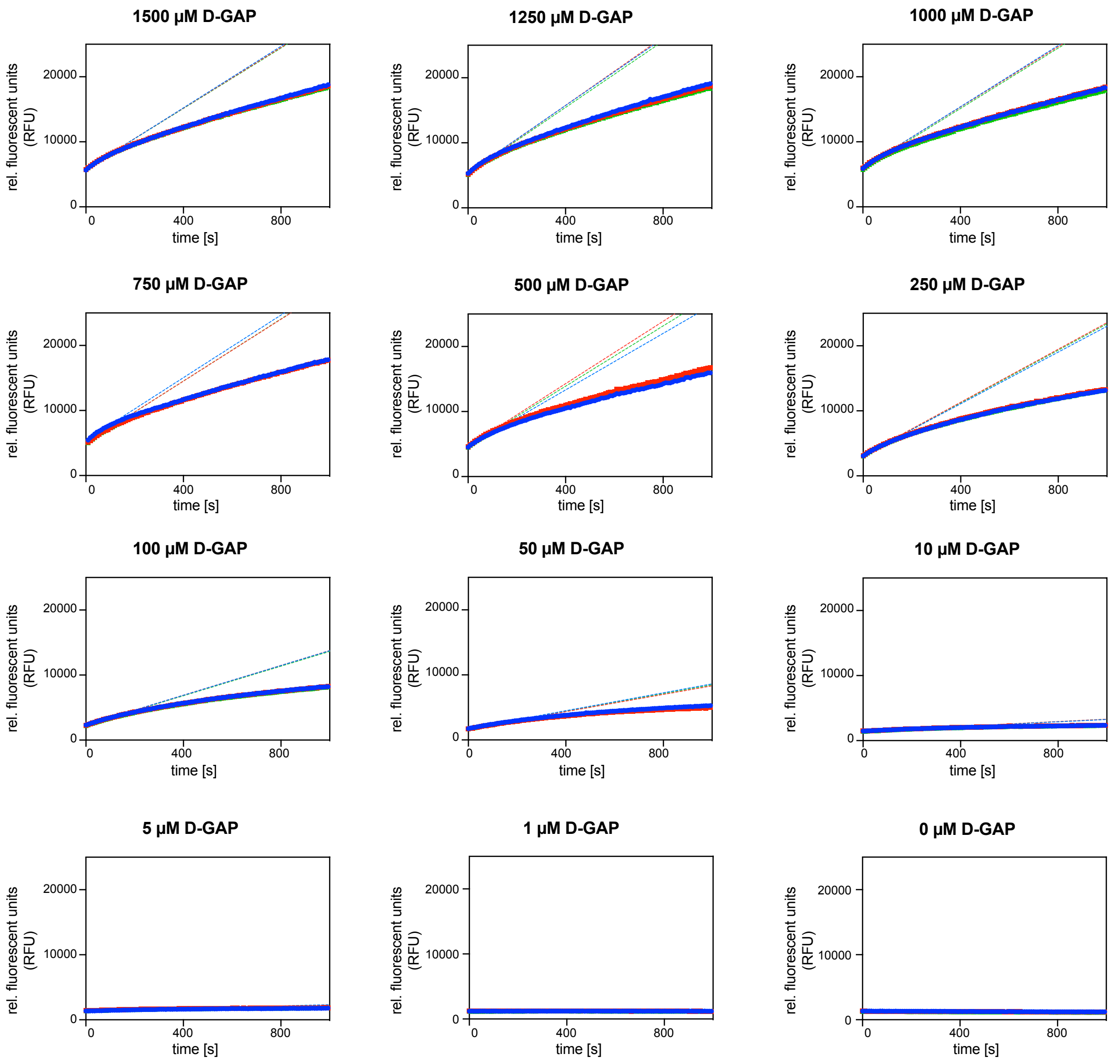

Supplementary Figure S7: Graphs displaying the linear regression of the initial reaction velocity of GAPDH wt at different substrate concentrations. NADH fluorescence was measured at 450 nm (excitation: 340 nm) for 15 minutes for different D-GAP concentrations and a simple linear regression model was used to determine the initial velocity of the first 100 sec of every measurement (dotted lines). Each measurement and regression was performed three times from biologically independent samples (depicted in blue, red, and green). All data processing was performed using GraphPad Prism 10 (GraphPad software).

GAPDH-K164GluK (BME)

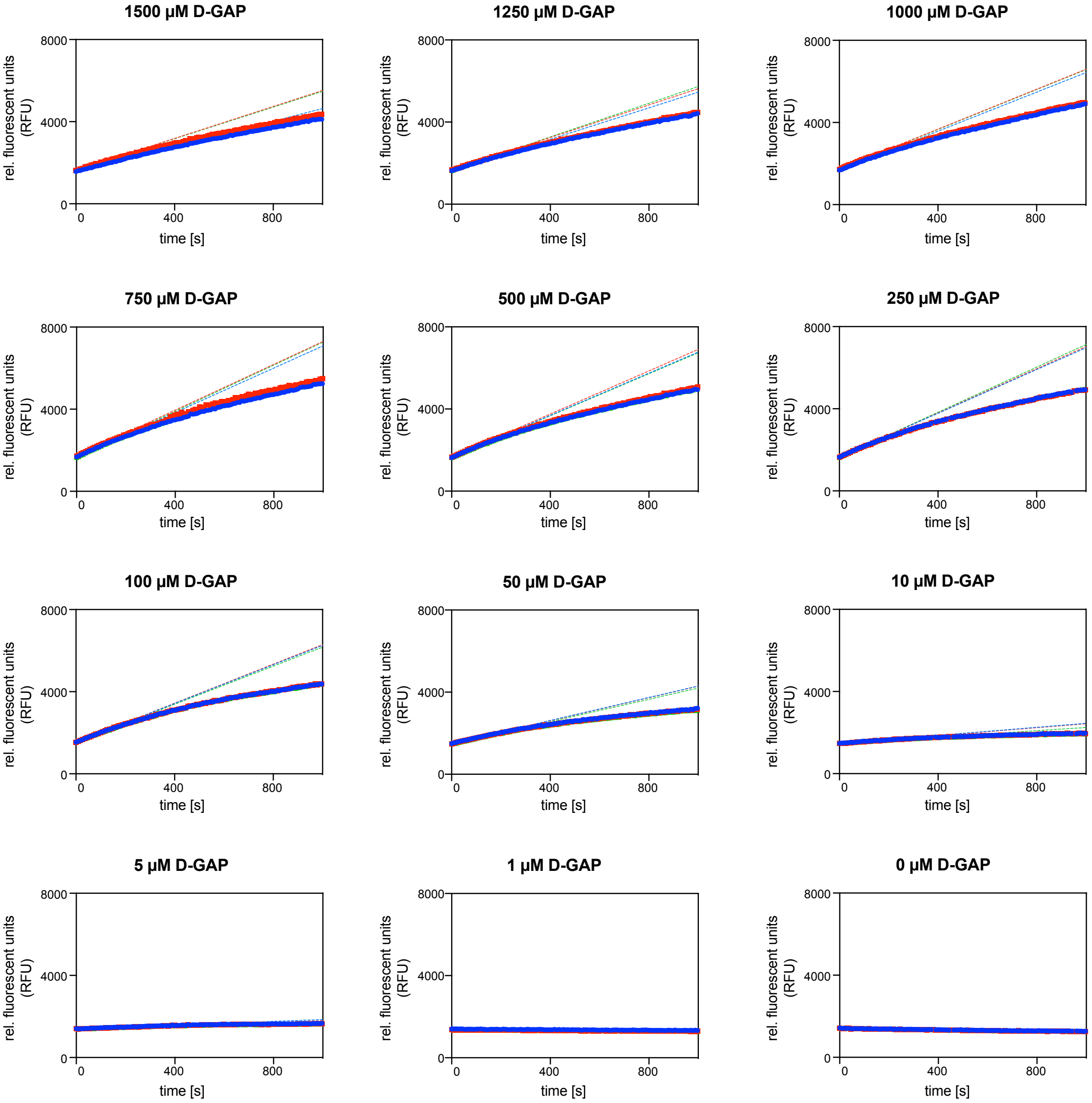

Supplementary Figure S8: Graphs displaying the linear regression of the initial reaction velocity of GAPDH-K194GluK (BME-treated) at different substrate concentrations. NADH fluorescence was measured at 450 nm (excitation: 340 nm) for 15 minutes for different D-GAP concentrations and a simple linear regression model was used to determine the initial velocity of the first 100 sec of every measurement (dotted lines). Each measurement and regression was performed three times from biologically independent samples (depicted in blue, red, and green). All data processing was performed using GraphPad Prism 10 (GraphPad software).

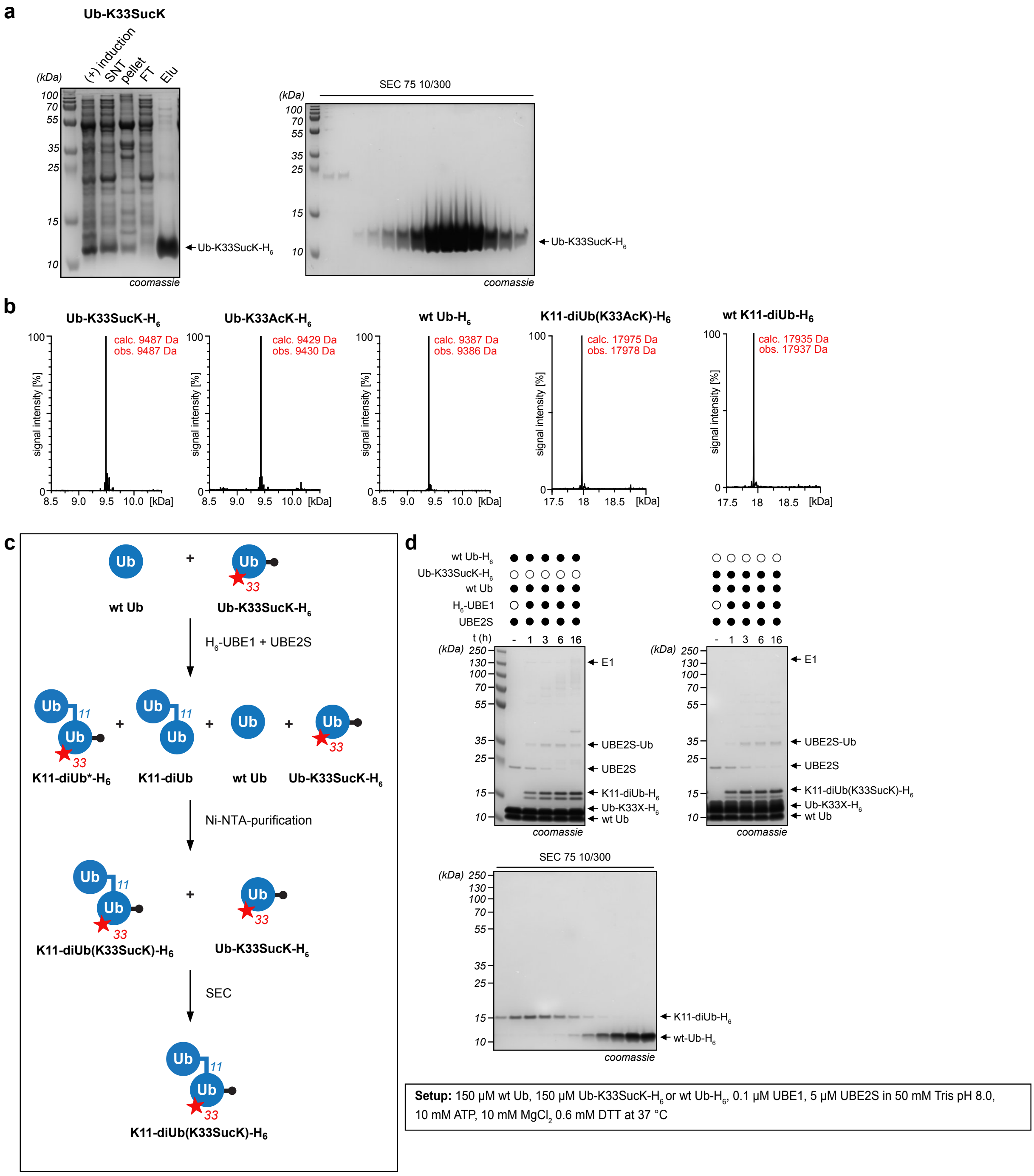

Supplementary Figure S9: Generation of succinylated K11-diUb via enzymatic assembly. a) SDS-PAGE analysis of Ni-NTA purification (left) and size exclusion column chromatography (Superdex 75 10/300, right) of Ub-K33SucK. b) LC-MS analysis confirmed the identity of Ub-K33SucK, Ub-K33AcK and wt Ub as well as K11-diUb(K33AcK) and wt K11-diUb. Non-deconvoluted spectra are shown in Supplementary Figure S17. c) Schematic representation of the enzymatic assembly and purification strategy of the K11-diUb(K33SucK)-H_6. d) SDS-PAGE analysis of the assembly (top) and purification of the K11-diUb(K33SucK)-H_6 (bottom). Consistent results were obtained over three biologically independent replicate experiments.

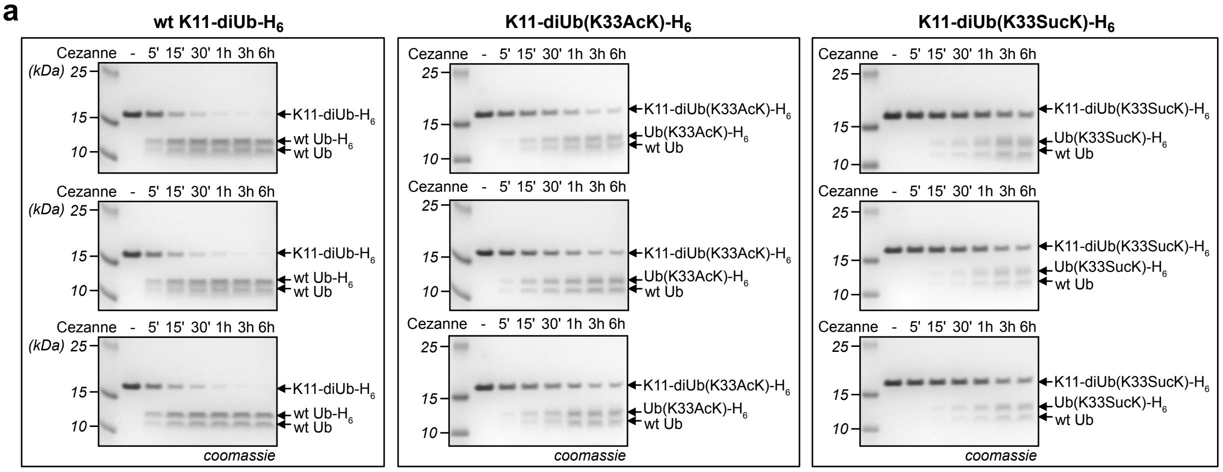

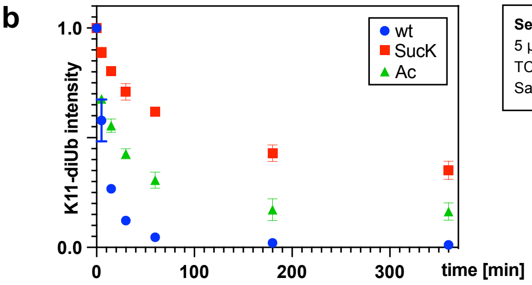

Setup: Cezanne DUB assay:
5 μM diUb, 7.5 nM Cezanne, 4 °C. buffer: 50 mM HEPES pH 7.4, 150 mM NaCl, 2 mM DTT, 2 mM TCEP. samples: 3.5 μL quenched by addition of 4xSDS loading buffer and boiling at 95 °C for 10 min. Samples were run on SDS-PAGE and visualized by Coomassie staining.

Supplementary Figure S10: diUb hydrolysis assays. a) SDS-PAGE analysis of DUB assays. K11-diUb(K33SucK)-H6, wt K11-diUb-H6 and K11-diUb(K33AcK)-H6 (all 5 μM) were individually incubated for the denoted time points with Cezanne (7.5 nM). Cezanne-mediated diUb cleavage is most severely impaired by succinylation of K33 in the proximal Ub. Consistent results were obtained over three distinct replicate experiments. b) Quantification of the diUb hydrolysis given as the relative band intensity of the K11-diUb band. Gel analysis was performed using ImageJ[5]. Data are presented as mean values ± SEM. Consistent results were obtained over three (n = 3) biologically independent replicate experiments. Full gels can be found in Supplementary Figure S15.

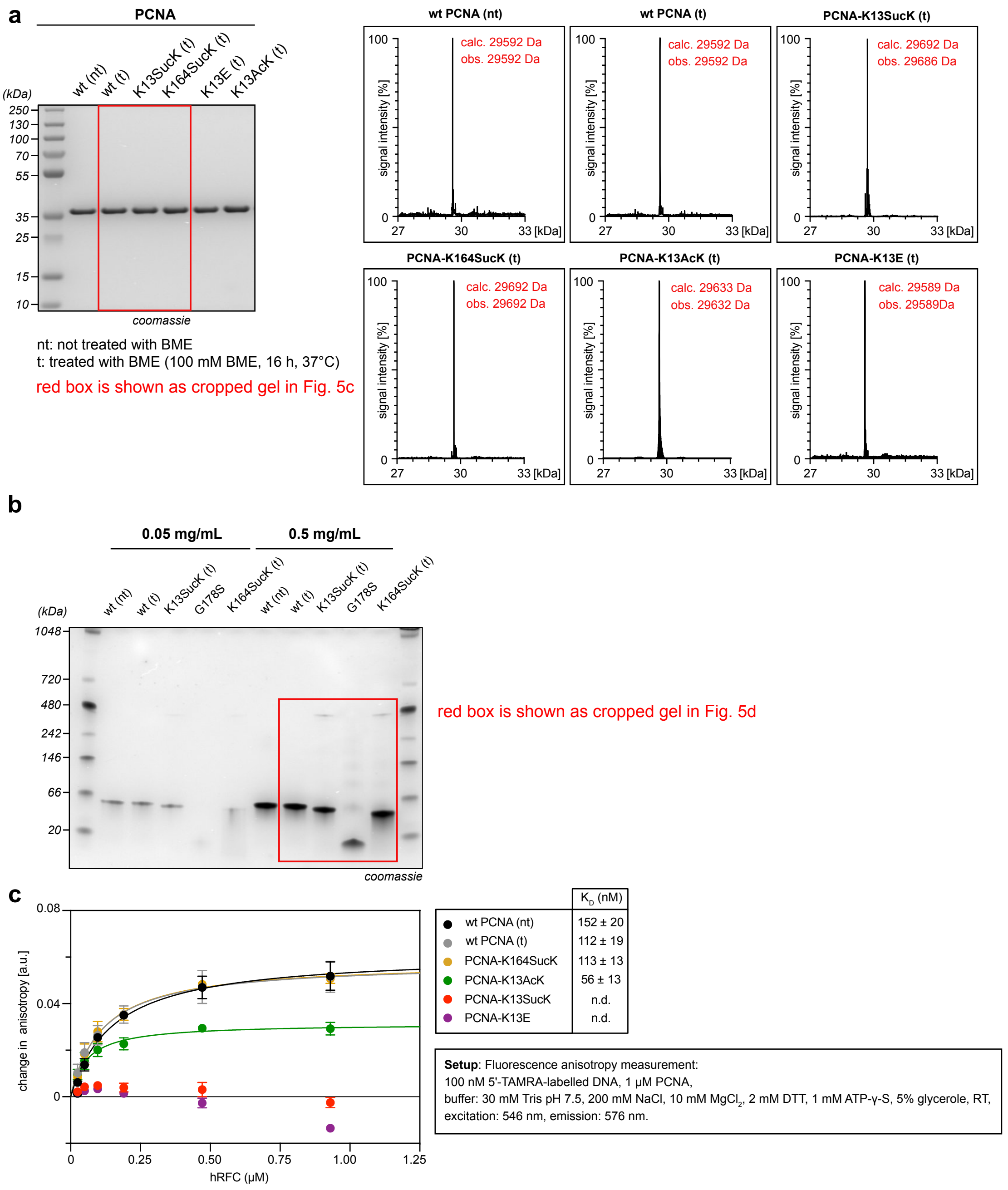

Supplementary Figure S11: Succinylation is involved in hRFC-mediated clamp loading onto DNA. a) SDS-PAGE analysis (left) and LC-MS analysis (right) of purified PCNA variants. Non-deconvoluted m/z spectra are shown in Supplementary Figure S18. b) Native PAGE analysis of wt PCNA and PCNA variants. Samples were incubated in appropriate buffers at 0.05 mg/mL and 0.5 mg/mL prior to native PAGE analysis. c) Fluorescence anisotropy measurements were conducted to determine the K_D of the interaction of PCNA and PCNA variants with DNA after being charged by hRFC. The change in anisotropy was plotted against the hRFC concentration and fitted with a single-site binding model to determine K_D values. Average values and errors (SEM) were calculated from three biologically independent experiments (n=3). All data processing was performed using GraphPad Prism 10 (GraphPad software).

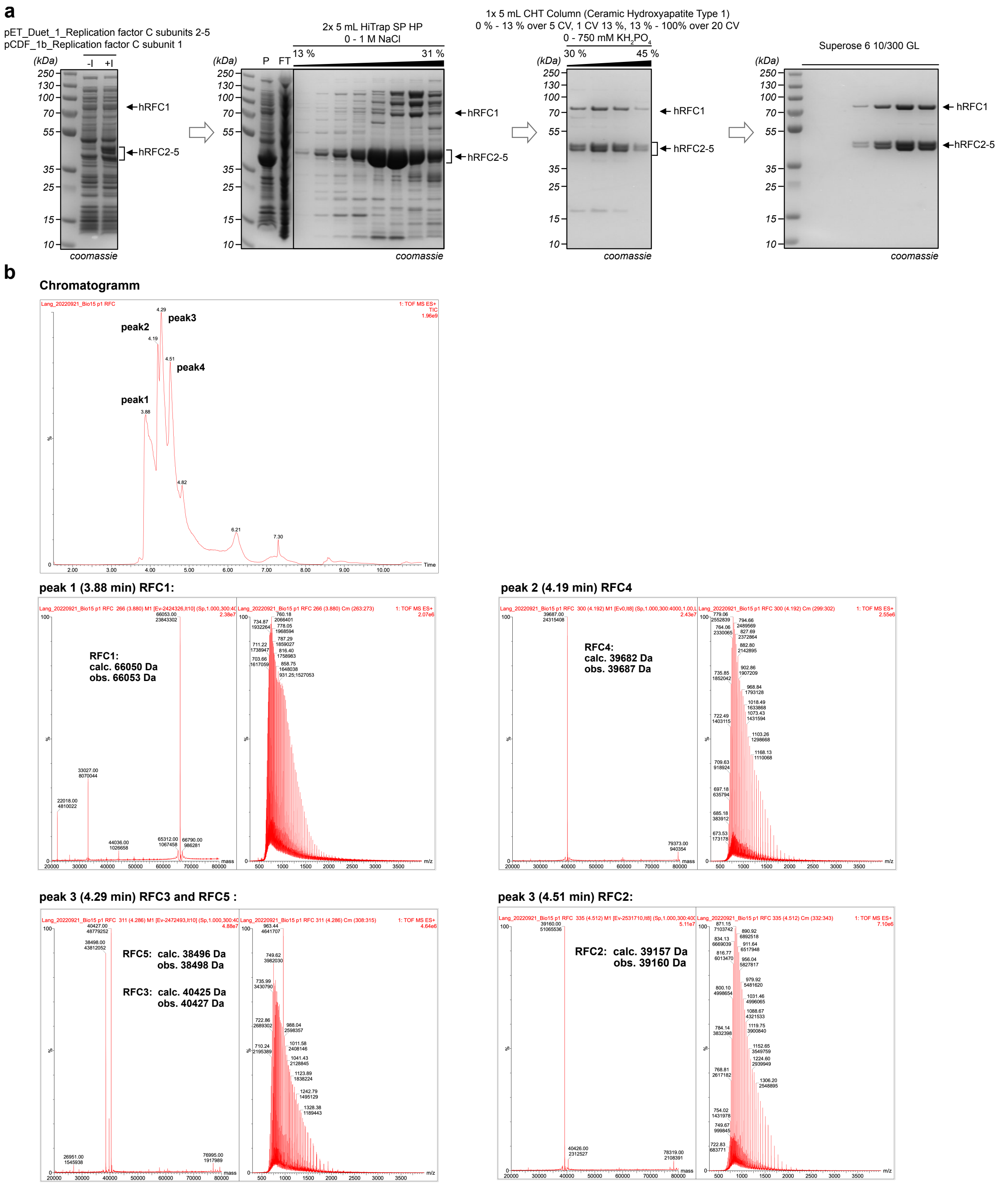

Supplementary Figure S12: Purification and characterization of hRFC. a) SDS-PAGE analysis of the purification of hRFC via HiTrap SP HP and CHT column chromatography followed by Superose 6 10/300 GL column chromatography. b) LC chromatogram and LC-MS analysis confirmed the integrity of all five hRFC subunits. Consistent results were obtained over three biologically independent replicate experiments.

full gel: Figure 1

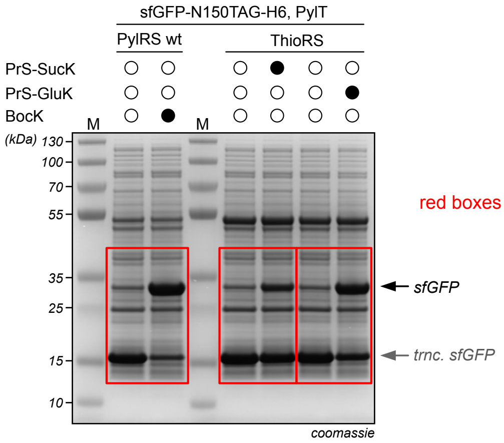

red boxes are shown as cropped gels in Fig. 1c

full gels and WBs: Figure 2

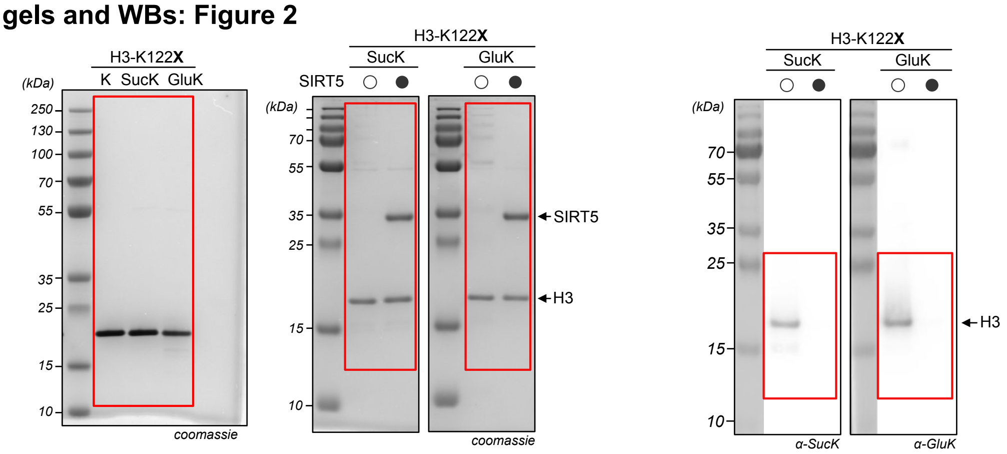

red boxes are shown as cropped gels in Fig. 2b and Fig. 2d

red boxes are shown as cropped WBs in Fig. 2d

full gels and WBs: Supplementary Figure S5

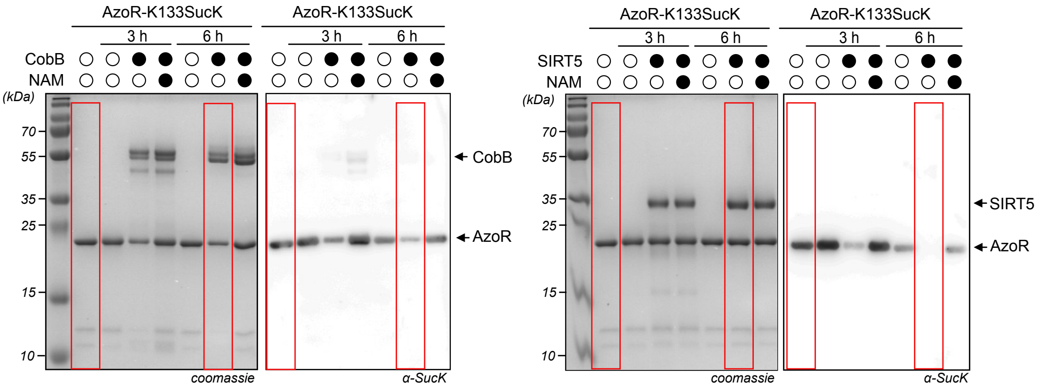

red boxes are shown as cropped gels in Supplementary Fig. S5c and S5d

Supplementary Figure S13: Full gels and Western blots. The red boxes indicate the areas shown in the denoted figures.

full gel: Figure 3b and Supplementary Figure S6

red boxes are shown as cropped gels in Fig. 3b

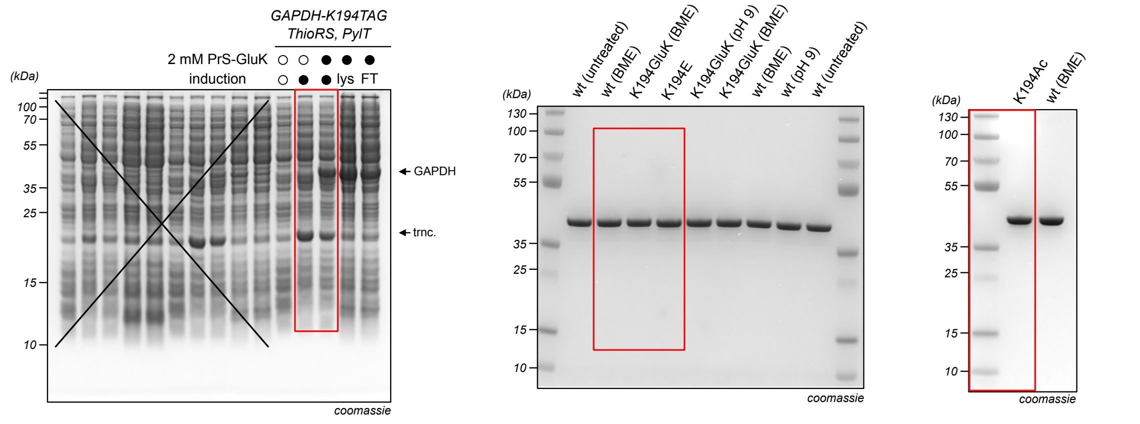

red boxes are shown as cropped gels in Fig. S6

full gel: Supplementary Figure S2a and S2b

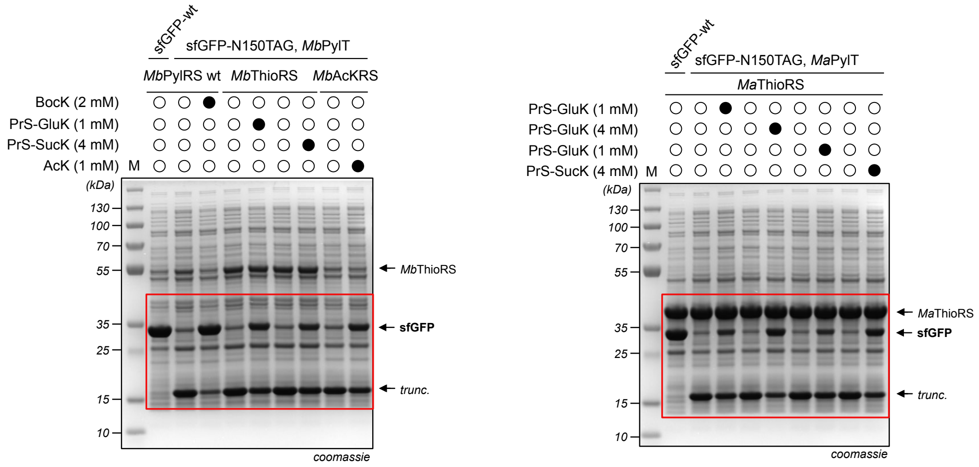

red boxes are shown as cropped gels in Supplementary Fig. S2a (left) and S2b (right)

Supplementary Figure S14: Full gels. The red boxes indicate the areas shown in the denoted figures.

full gel: Figure 4d and S10 a

red boxes are shown as cropped gels in Fig. 4d .

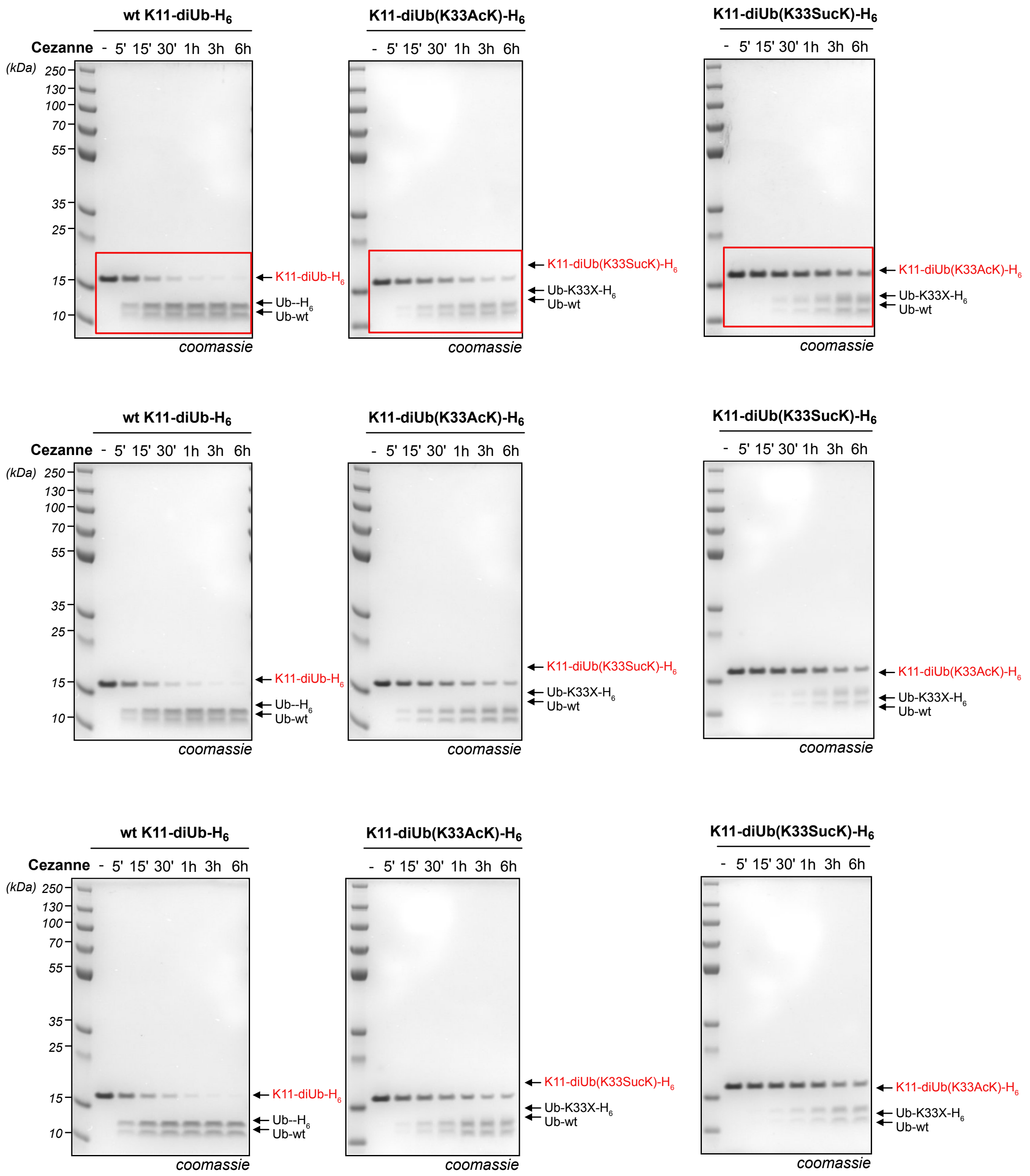

Supplementary Figure S15: Full gels. The red boxes indicate the areas shown in the denoted figures.

LC-MS: sfGFP variants (Figure 1d)

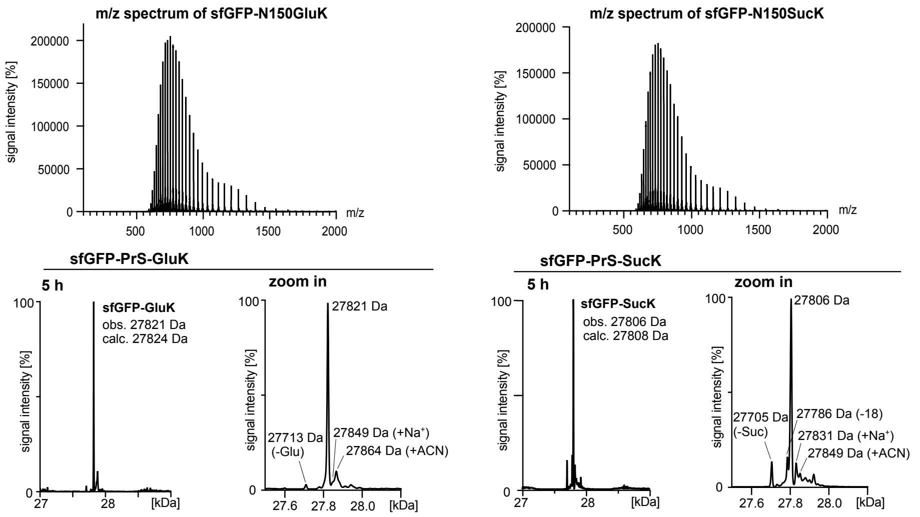

LC-MS: Histones (Supplementary Figure 4c)

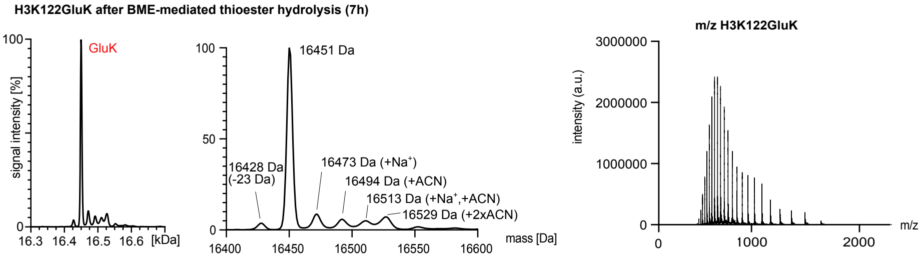

LC-MS: GAPDH and GAPDH variants (Supplementary Figure S6)

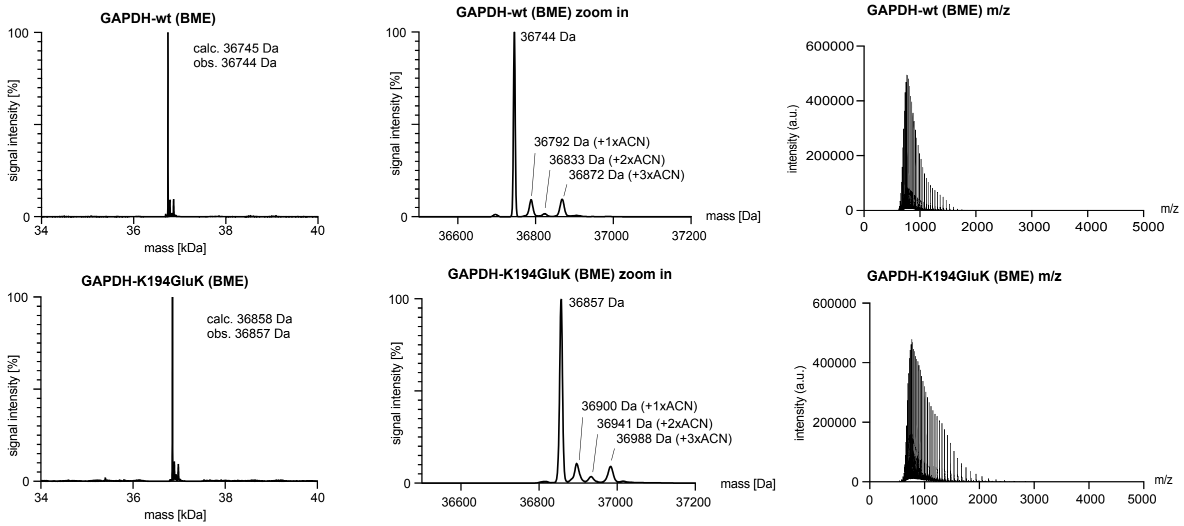

Supplementary Figure S16: Comprehensive LC-MS analysis of sfGFP, Histones, and GAPDH. For every POI, representative deconvoluted spectra and m/z spectra are shown for the wt protein and the SucK/GluK bearing POI. Consistent results were obtained over three biologically independent replicate experiments.

LC-MS: monoUbs (Supplementary Figure S9)

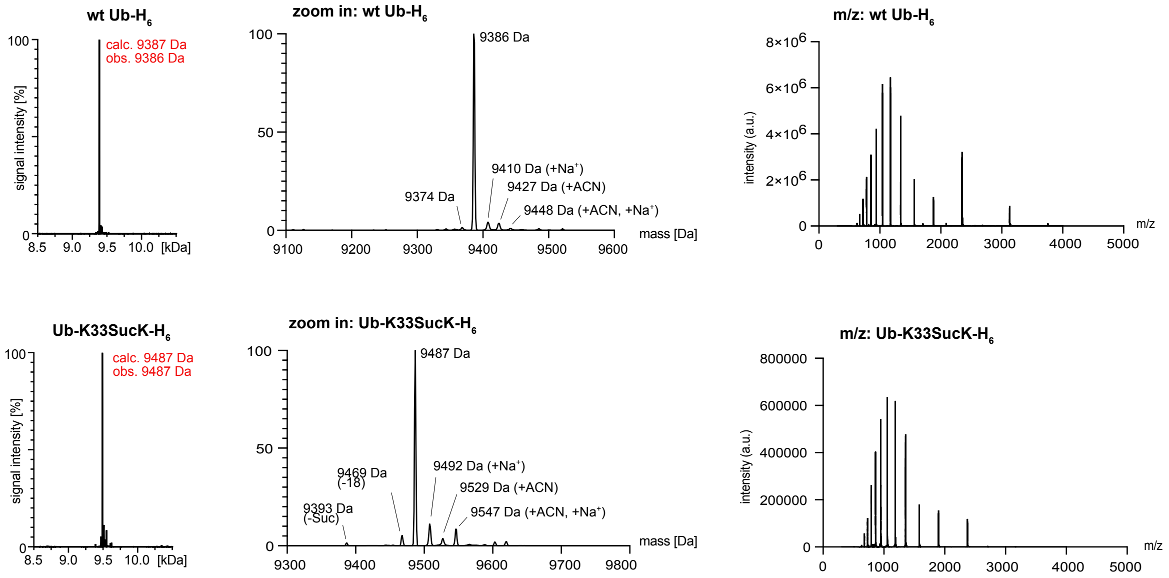

LC-MS: diUbs (Figure 4 and Supplementary Figure S9)

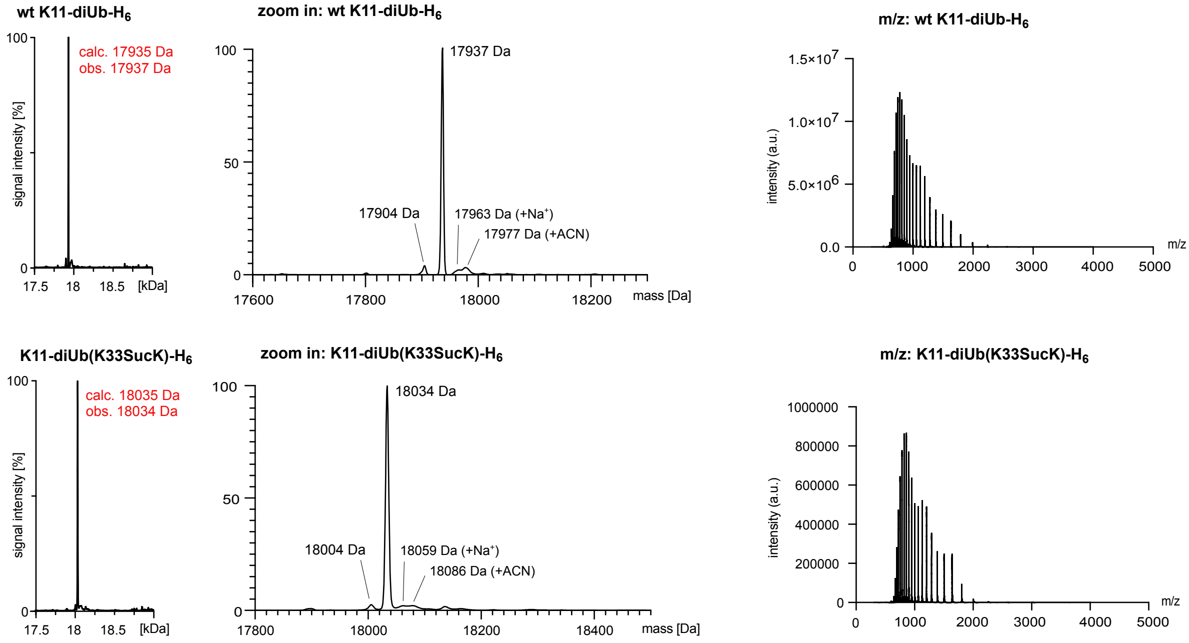

Supplementary Figure S17: Comprehensive LC-MS analysis of monoUbs and diUbs. Representative deconvoluted spectra and m/z spectra are shown for the wt proteins and the SucK bearing POIs. Consistent results were obtained over three biologically independent replicate experiments.

LC-MS: PCNA (Supplementary Figure S11)

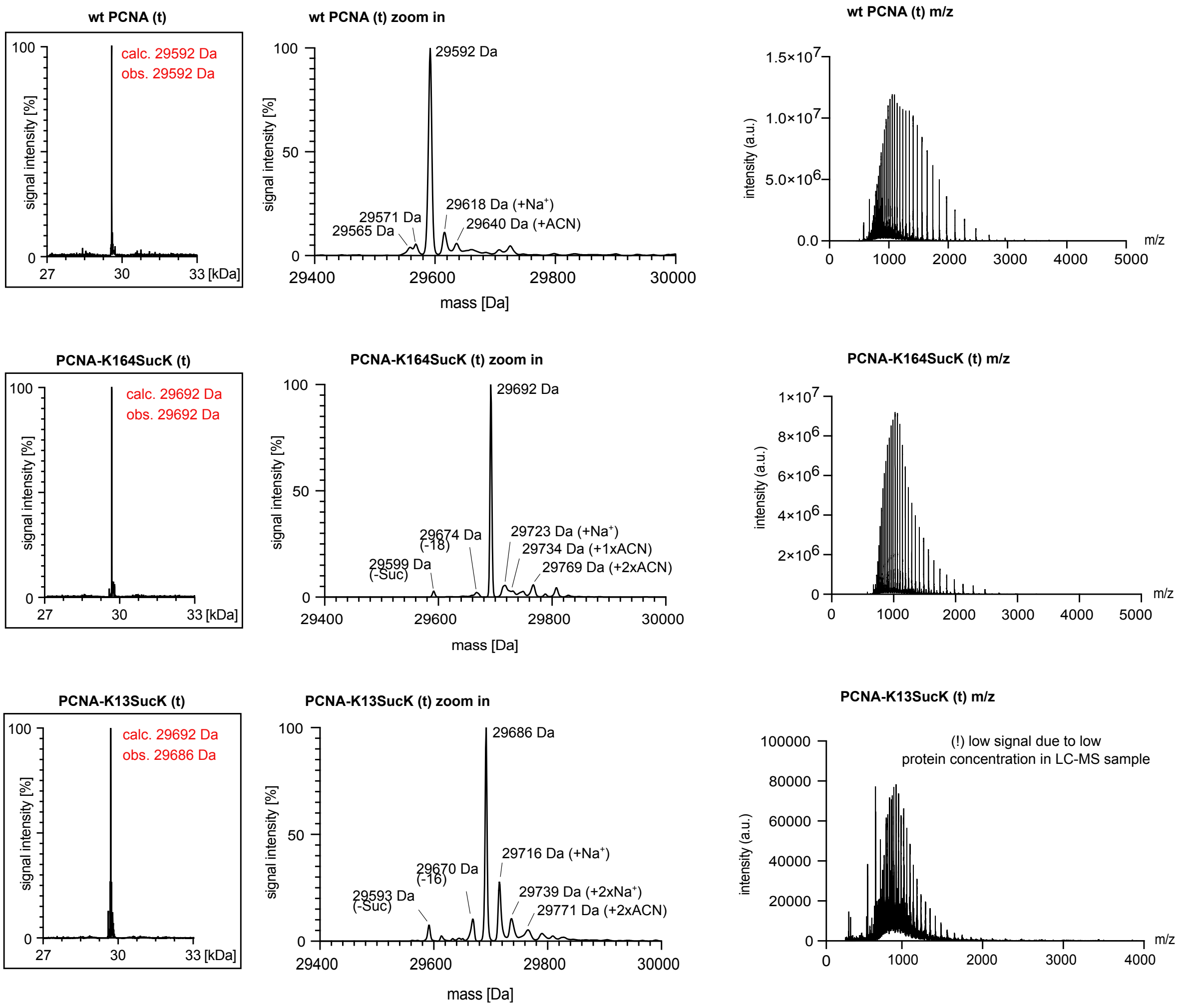

Supplementary Figure S18: Comprehensive LC-MS analysis of PCNA. Representative deconvoluted spectra and m/z spectra are shown for wt PCNA and SucK bearing PCNA. Consistent results were obtained over three biologically independent replicate experiments.

Experimental procedures

1. General methods: Plasmids and reagents

Codon optimized genes encoding Histone H3, AzoR, PCNA (proliferating cell nuclear antigen), and Sirtuin 5 (SIRT5) were purchased as DNA Strings (Twist Bioscience and GeneArt) and cloned into the pBAD, pPylT, or pET17 vectors via restriction cloning (see Supplementary Table S3). Point mutations, insertions, and deletions were introduced using Site-directed, Ligase-Independent Mutagenesis[6] (SLIM, see Supplementary Table S1). GAPDH (glyceraldehyde 3-phosphate dehydrogenase) as well as hRFC (human replication factor C) vectors were purchased (Addgene plasmids #83910, #175043, and #175049). pOPINK-UBE2S was a gift from Anja Bremm, Institute of Biochemistry II, Goethe University Frankfurt. pGEX-4T1 GST-Thr-TEV-CobB 40-274 was a gift from Michael Lammers, University of Greifswald. Oligonucleotide primers were designed with NEBuilder and purchased from Sigma Aldrich or Microsynth (see Supplementary Tables S1). Amino acid sequences of all proteins are listed in Supplementary Note 1.4.

All solvents and chemical reagents were purchased from Sigma Aldrich, Senn, Carbolution, Acros Organics, or Fisher Scientific and were used without further purification unless stated otherwise. Bolt 4-12 % Bis-Tris gradient gels (Invitrogen) or self-casted 15 % SDS-PAGE gels were run (at 160 V for 60 min) using a Bolt™ Mini Gel Tank system (Invitrogen). Gels were stained with Quick Coomassie Stain (Generon). PageRuler Prestained Plus Protein Ladder 10-250 kDa (ThermoFisher) was used as the protein marker. Western blots were carried out on iBlot 2 Dry Blotting System (Life Technologies) using Method P0 (20 V for 1 min, 23 V for 4 min, 25 V for 2 min). After blotting, the nitrocellulose/PVDF membrane was blocked with 5 % skim milk powder solution in 1× TBS-T buffer (1 h, RT) and stained with the desired antibody according to the manufacturer's instructions. Proteins were visualized with Immobilon® Forte Western HRP (horseradish peroxidase) Substrate (Millipore) using Amersham ImageQuant 800 (Cytiva). Protein and DNA concentrations were measured on a NanoPhotometer® NP60 (Implen). H6-UBE1 was purchased from Boston Biochem (Cat. No. E-304-050). Ni-NTA agarose was purchased from Jena Bioscience (Cat. No. AC-501).

1.1 Primers

Supplementary Table S1: Primers for introduction of TAG mutants.

Construct	Primer	Sequence (5' → 3')
pPylT_Ub-K33TAG-H6	short fw	CATCCCTCCTGACCAGC
	short rv	GATCTTTGCCTTGACATTCTC
	tail fw	CAAGACTAGGAAGGCATCCCTCCTGAC
	tail rv	CCTTCCTAGTCTTGGATCTTTGCCTTG
pBAD_Histone H3-K122TAG-H6	short fw	CTGGCCCGCAGAATCC
	short rv	GGGCATGATGGTGACCC

	tail fw	TAGGACATCCAGCTGGCCC
	tail rv	CTGGATGTCCTAGGGCATGATGGTGACCC
pBAD_PCNA-K13TAG-H6	short fw	GTTGGAGGCACTCAAGGACC
	short rv	GGAGCCCTGGACCAGGC
	tail fw	ATCCTCTAGAAGGTGTTGGAGGCACTC
	tail rv	ACCTTCTAGAGGATGGAGCCCTGGACC
pBAD_PCNA-K164TAG-H6	short fw	GAGTGAAATTTTCTGCAAG
	short rv	GAAATTACAACAGCATCTCC
	tail fw	CTGTGCATAGGACGGAGTGAAATTTTC
	tail rv	CGTCCTATGCACAGGAAATTACAAC
pPylT_GAPDH-K194TAG-H6	short fw	GATGGCCGCGGG
	short rv	CCCGGAGGGGCC
	tail fw	TAGCTGTGGCGTGATGGCC
	tail rv	ACGCCACAGCTACCCGGAGGGGCC
pPylT_AzoR-K133TAG-H6	short fw	GTTATTACCAGCCGCG
	short rv	ACCCGTTACCAGACCTTC
	tail fw	TAGAAAGCCATCGTTATTACCAGC
	tail rv	GATGGCTTTCTAACCCGTTACCAGACC
pPylT_GAPDH-K194E-H6	short fw	GATGGCCGCGGG
	short rv	CCCGGAGGGGCC
	tail fw	GAACTGTGGCGTGATGGCC
	tail rv	ACGCCACAGTTCCCCGGAGGGGCC
pBAD_PCNA-G178S-H6	short fw	TTGTCACAGACAAGTAATGTCG
	short rv	ATTTCCAAGTTCTCCACTTGC
	tail fw	AGTAACATTAAATTGTCACAGACAAGTAATGTCG
	tail rv	TTTAATGTTACTATTTCCAAGTTCTCCACTTGC

1.2 Oligonucleotides for fluorescence anisotropy

Supplementary Table S2: Oligonucleotides for fluorescence anisotropy.

Oligo	Modification	Sequence (5' → 3')
1	5`-TAMRA	GAGCATTCAAGGACTTAGGTCTGTATTTATCTACCCACAA
2	none	TTGTGGGTAGATAAATACAGACCTAAGTCC

1.3 Plasmids

Supplementary Table S3: Plasmids.

Plasmid	Description
pBAD_sfGFP-wt-H6	sfGFP (superfolder green fluorescent protein) wt (wild-type)-H6 under an arabinose promotor with a C-terminal H6-tag (ampicillin resistance).
pBAD_sfGFP-N150TAG-H6	sfGFP-N150TAG-H6 under an arabinose promotor with a C-terminal H6-tag (ampicillin resistance).
pBK_wtRS	*M. barkeri* wt pyrrolysyl-tRNA-synthetase (PylRS) under a constitutive GlnS promoter (ampicillin resistance).
pBK_ThioRS	*M. barkeri* "ThioRS" PylRS under a constitutive GlnS promoter (ampicillin resistance).
pBK_MaThioRS	*M. alvus* "*Ma*ThioRS" PylRS under a constitutive GlnS promoter (ampicillin resistance).
pEVOL_wtRS_PylT	*M. barkeri* wt PylRS under a constitutive GlnS and an arabinose inducible promoter with a pyrrolysyl-tRNA (PylT) copy under a constitutive promotor. (chloramphenicol resistance).
pEVOL_ThioRS_PylT	*M. barkeri* "ThioRS" PylRS under a constitutive GlnS and an arabinose inducible promoter with a PylT copy under a constitutive promotor (chloramphenicol resistance).
pEVOL_MaThioRS_PylT	*M. alvus* "*Ma*ThioRS" PylRS under a constitutive GlnS and an arabinose inducible promoter with a *M. alvus* PylT copy under a constitutive promotor (chloramphenicol resistance).
pBAD_Histone H3 wt-H6	Histone H3 wt-H6 under an arabinose promotor with a C-terminal H6-tag (ampicillin resistance).
pBAD_Histone H3-K122TAG-H6	Histone H3-K122TAG-H6 under an arabinose promotor with a C-terminal H6-tag (ampicillin resistance).

pPylT_AzoR wt-H6	AzoR (FMN-dependent NADH:quinone oxidoreductase) wt-H6 under an arabinose promotor with a C-terminal H6-tag and a PylT copy under a constitutive promotor (tetracycline resistance).
pPylT_AzoR-K133TAG-H6	AzoR-K133TAG-H6 under an arabinose promotor with a C-terminal H6-tag and a PylT copy under a constitutive promotor (tetracycline resistance).
pET17_SIRT5	H6-TEV-SIRT5 (Sirtuin 5) without a mitochondrial localization signal (MLS) under a T7 promotor (ampicillin resistance).
pGEX_CobB	GST-thrombin-TEV-CobB under an T7 promotor (gift from Michael Lammers, University of Greifswald) (ampicillin resistance).
pPylT_GAPDH wt-H6	GAPDH wt-H6 under an arabinose promotor with a C-terminal H6-tag and a PylT copy under a constitutive promotor (tetracycline resistance).
pPylT_GAPDH-K194TAG-H6	GAPDH-K194TAG-H6 under an arabinose promotor with a C-terminal H6-tag and a PylT copy under a constitutive promotor (tetracycline resistance).
pPylT_GAPDH-K194E-H6	GAPDH-K194E-H6 under an arabinose promotor with a C-terminal H6-tag and a PylT copy under a constitutive promotor (tetracycline resistance).
pET17b_Ub wt	Ub (Ubiquitin) wt under an IPTG inducible T7 Promotor (ampicillin resistance).
pPylT_Ub wt-H6	Ub wt-H6 under an arabinose promotor with a C-terminal H6-tag and a PylT copy under a constitutive promotor (tetracycline resistance).
pPylT_Ub-K33TAG-H6	Ub-K33TAG-H6 under an arabinose promotor with a C-terminal H6-tag and a PylT copy under a constitutive promotor (tetracycline resistance).
pOPINK_UBE2S	H6-GST-3C-UBE2S(1-196) under an T7 promotor (kanamycin resistance).
pBAD_PCNA wt-H6	PCNA wt-H6 under an arabinose promotor with a C-terminal H6-tag (ampicillin resistance).
pBAD_PCNA-K13TAG-H6	PCNA-K13TAG-H6 under an arabinose promotor with a C-terminal H6-tag (ampicillin resistance).
pBAD_PCNA-K164TAG-H6	PCNA-K164TAG-H6 under an arabinose promotor with a C-terminal H6-tag (ampicillin resistance).

pBAD_PCNA-G178S-H6	PCNA-G178S-H6 under an arabinose promotor with a C-terminal H6-tag (ampicillin resistance).

1.4 Amino acid sequences of proteins

sfGFP wt-H6
MPSKGEELFTGVVPILVELDGDVNGHKFSVRGEGEGDATNGKLTLKFICTTGKLPVPWPTLV
TTLTYGVQCFSRYPDHMKRHDFFKSAMPEGYVQERTISFKDDGTYKTRAEVKFEGDTLVNR
IELKGIDFKEDGNILGHKLEYNFNSHNVYITADKQKNGIKANFKIRHNVEDGSVQLADHYQQN
TPIGDGPVLLPDNHYLSTQSVLSKDPNEKRDHMVLLEFVTAAGITHGMDELYKGSHHHHHH*

sfGFP-N150TAG-H6
MPSKGEELFTGVVPILVELDGDVNGHKFSVRGEGEGDATNGKLTLKFICTTGKLPVPWPTLV
TTLTYGVQCFSRYPDHMKRHDFFKSAMPEGYVQERTISFKDDGTYKTRAEVKFEGDTLVNR
IELKGIDFKEDGNILGHKLEYNFNSH*VYITADKQKNGIKANFKIRHNVEDGSVQLADHYQQN
TPIGDGPVLLPDNHYLSTQSVLSKDPNEKRDHMVLLEFVTAAGITHGMDELYKGSHHHHHH*

M. barkeri ThioRS (*Mb*ThioRS)
MDKKPLDVLISATGLWMSRTGTLHKIKHHEISRSKIYIEMACGDHLVVNNSRSCRPARAFRY
HKYRKTCKRCRVSDEDINNFLTRSTESKNSVKVRVVSEPKVKKAMPKSVSRAPKPLENSVS
AKASTNTSRSVPSPAKSTPNSSVPASAPAPSLTRSQLDRVEALLSPEDKISLNMAKPFRELE
PELVTRRKNDFQRLYTNDREDYLGKLERDITKFFVDRGFLEIKSPILIPAEYVERMGINNDTEL
SKQIFRVDKNLCLRPMLAPTLANYLRKLDRILPGPIKIFEVGPCYRKESDGKEHLEEFTMVNF
VQMGSGCTRENLEALIKEFLDYLEIDFEIVGDSCMVYGDTLDIMHGDLELSSAVVGPVSLDR
EWGIDKPWIGAGFGLERLLKVMHGFKNIKRASRSESYYNGISTNL*

M. alvus ThioRS (*Ma*ThioRS)
MTVKYTDAQIQRLREYGNGTYEQKVFEDLASRDAAFSKEMSVASTDNEKKIKGMIANPSRH
GLTQLMNDIADALVAEGFIEVRTPIFISKDALARMTITEDKPLFKQVFWIDEKRALRPMLAPNL
ASVMRDLRDHTDGPVKIFEMGSCFRKESHSGMHLEEFTMLNLVDMGPRGDATEVLKNYISV
VMKAAGLPDYDLVQEESDVYKETIDVEINGQEVCSAAVGPIPLDAAHDVHEPWSGAGFGLE
RLLTIREKYSTVKKGGASISYLNGAKIN*

Histone H3 wt-H6
MARTKQTARKSTGGKAPRKQLATKAARKSAPATGGVKKPHRYRPGTVALREIRRYQKSTEL
LIRKLPFQRLVREIAQDFKTDLRFQSSAVMALQEASEAYLVALFEDTNLCAIHAKRVTIMPKDI
QLARRIRGERARSHHHHHH*

Histone H3-K122TAG-H6
MARTKQTARKSTGGKAPRKQLATKAARKSAPATGGVKKPHRYRPGTVALREIRRYQKSTEL
LIRKLPFQRLVREIAQDFKTDLRFQSSAVMALQEASEAYLVALFEDTNLCAIHAKRVTIMP*DI
QLARRIRGERARSHHHHHH*

AzoR-K133TAG-H6
MSKVLVLKSSILAGYSQSNQLSDYFVEQWREKHSADEITVRDLAANPIPVLDGELVGALRPS
DAPLTPRQQEALALSDELIAELKAHDVIVIAAPMYNFNISTQLKNYFDLVARAGVTFRYTENG

PEGLVTG*KAIVITSRGGIHKDGPTDLVTPYLSTFLGFIGITDVKFVFAEGIAYGPEMAAKAQS
DAKAAIDSIVSAHHHHHH*

CobB
MSPILGYWKIKGLVQPTRLLLEYLEEKYEEHLYERDEGDKWRNKKFELGLEFPNLPYYIDGD
VKLTQSMAIIRYIADKHNMLGGCPKERAEISMLEGAVLDIRYGVSRIAYSKDFETLKVDFLSKL
PEMLKMFEDRLCHKTYLNGDHVTHPDFMLYDALDVVLYMDPMCLDAFPKLVCFKKRIEAIP
QIDKYLKSSKYIAWPLQGWQATFGGGDHPPKSDLVPRGSENLYFQGGSGKPRVLVLTGAGI
SAESGIRTFRAADGLWEEHRVEDVATPEGFDRDPELVQAFYNARRRQLQQPEIQPNAAHLA
LAKLQDALGDRFLLVTQNIDNLHERAGNTNVIHMHGELLKVRCSQSGQVLDWTGDVTPEDK
CHCCQFPAPLRPHVVWFGEMPLGMDEIYMALSMADIFIAIGTSGHVYPAAGFVHEAKLHGA
HTVELNLEPSQVGNEFAEKYYGPASQVVPEFVEKLLKGLK*

SIRT5
MHHHHHHGGENLYFQGSSSMADFRKFFAKAKHIVIISGAGVSAESGVPTFRGAGGYWRKW
QAQDLATPLAFAHNPSRVWEFYHYRREVMGSKEPNAGHRAIAECETRLGKQGRRVVVITQ
NIDELHRKAGTKNLLEIHGSLFKTRCTSCGVVAENYKSPICPALSGKGAPEPGTQDASIPVEK
LPRCEEAGCGGLLRPHVVWFGENLDPAILEEVDRELAHCDLCLVVGTSSVVYPAAMFAPQV
AARGVPVAEFNTETTPATNRFRFHFQGPCGTTLPEALACHENETVS*

GAPDH wt-H6 (addgene #83910)
MGKVKVGVNGFGRIGRLVTRAAFNSGKVDIVAINDPFIDLNYMVYMFQYDSTHGKFHGTVK
AENGKLVINGNPITIFQERDPSKIKWGDAGAEYVVESTGVFTTMEKAGAHLQGGAKRVIISAP
SADAPMFVMGVNHEKYDNSLKIISNASCTTNCLAPLAKVIHDNFGIVEGLMTTVHAITATQKT
VDGPSGKLWRDGRGALQNIIPASTGAAKAVGKVIPELNGKLTGMAFRVPTANVSVVDLTCR
LEKPAKYDDIKKVVKQASEGPLKGILGYTEHQVVSSDFNSDTHSSTFDAGAGIALNDHFVKLI
SWYDNEFGYSNRVVDLMAHMASKEHHHHHH*

GAPDH-K194TAG-H6
MGKVKVGVNGFGRIGRLVTRAAFNSGKVDIVAINDPFIDLNYMVYMFQYDSTHGKFHGTVK
AENGKLVINGNPITIFQERDPSKIKWGDAGAEYVVESTGVFTTMEKAGAHLQGGAKRVIISAP
SADAPMFVMGVNHEKYDNSLKIISNASCTTNCLAPLAKVIHDNFGIVEGLMTTVHAITATQKT
VDGPSG*LWRDGRGALQNIIPASTGAAKAVGKVIPELNGKLTGMAFRVPTANVSVVDLTCRL
EKPAKYDDIKKVVKQASEGPLKGILGYTEHQVVSSDFNSDTHSSTFDAGAGIALNDHFVKLIS
WYDNEFGYSNRVVDLMAHMASKEHHHHHH*

GAPDH-K194E-H6
MGKVKVGVNGFGRIGRLVTRAAFNSGKVDIVAINDPFIDLNYMVYMFQYDSTHGKFHGTVK
AENGKLVINGNPITIFQERDPSKIKWGDAGAEYVVESTGVFTTMEKAGAHLQGGAKRVIISAP
SADAPMFVMGVNHEKYDNSLKIISNASCTTNCLAPLAKVIHDNFGIVEGLMTTVHAITATQKT
VDGPSGELWRDGRGALQNIIPASTGAAKAVGKVIPELNGKLTGMAFRVPTANVSVVDLTCR
LEKPAKYDDIKKVVKQASEGPLKGILGYTEHQVVSSDFNSDTHSSTFDAGAGIALNDHFVKLI
SWYDNEFGYSNRVVDLMAHMASKEHHHHHH*

Ub wt
MQIFVKTLTGKTITLEVEPSDTIENVKAKIQDKEGIPPDQQRLIFAGKQLEDGRTLSDYNIQKE
STLHLVL RLRGG*

Ub wt-H6
MQIFVKTLTGKTITLEVEPSDTIENVKAKIQDKEGIPPDQQRLIFAGKQLEDGRTLSDYNIQKE
STLHLVLRLRGGHHHHHH*

Ub-K33TAG-H6
MQIFVKTLTGKTITLEVEPSDTIENVKAKIQD*EGIPPDQQRLIFAGKQLEDGRTLSDYNIQKES
TLHLVLRLRGGHHHHHH*

H6-GST-3C-UBE2S
HHHHHHMSPILGYWKIKGLVQPTRLLLEYLEEKYEEHLYERDEGDKWRNKKFELGLEFPNL
PYYIDGDVKLTQSMAIIRYIADKHNMLGGCPKERAEISMLEGAVLDIRYGVSRIAYSKDFETLK
VDFLSKLPEMLKMFEDRLCHKTYLNGDHVTHPDFMLYDALDVVLYMDPMCLDAFPKLVCFK
KRIEAIPQIDKYLKSSKYIAWPLQGWQATFGGGDHPPKSDLSSGLEVLFQGPMNSNVENLPP
HIIRLVYKEVTTLTADPPDGIKVFPNEEDLTDLQVTIEGPEGTPYAGGLFRMKLLLGKDFPASP
PKGYFLTKIFHPNVGANGEICVNVLKRDWTAELGIRHVLLTIKCLLIHPNPESALNEEAGRLLL
ENYEEYAARARLLTEIHGGAGGPSGRAEAGRALASGTEASSTDPGAPGGPGGAEGPMA*

PCNA wt-H6
MFEARLVQGSILKKVLEALKDLINEACWDISSSGVNLQSMDSSHVSLVQLTLRSEGFDTYRC
DRNLAMGVNLTSMSKILKCAGNEDIITLRAEDNADTLALVFEAPNQEKVSDYEMKLMDLDVE
QLGIPEQEYSCVVKMPSGEFARICRDLSHIGDAVVISCAKDGVKFSASGELGNGNIKLSQTS
NVDKEEEAVTIEMNEPVQLTFALRYLNFFTKATPLSSTVTLSMSADVPLVVEYKIADMGHLKY
YLAPKIEDEEGSHHHHHH*

PCNA-K13TAG-H6
MFEARLVQGSIL*KVLEALKDLINEACWDISSSGVNLQSMDSSHVSLVQLTLRSEGFDTYRC
DRNLAMGVNLTSMSKILKCAGNEDIITLRAEDNADTLALVFEAPNQEKVSDYEMKLMDLDVE
QLGIPEQEYSCVVKMPSGEFARICRDLSHIGDAVVISCAKDGVKFSASGELGNGNIKLSQTS
NVDKEEEAVTIEMNEPVQLTFALRYLNFFTKATPLSSTVTLSMSADVPLVVEYKIADMGHLKY
YLAPKIEDEEGSHHHHHH*

PCNA-K164TAG-H6
MFEARLVQGSILKKVLEALKDLINEACWDISSSGVNLQSMDSSHVSLVQLTLRSEGFDTYRC
DRNLAMGVNLTSMSKILKCAGNEDIITLRAEDNADTLALVFEAPNQEKVSDYEMKLMDLDVE
QLGIPEQEYSCVVKMPSGEFARICRDLSHIGDAVVISCA*DGVKFSASGELGNGNIKLSQTSN
VDKEEEAVTIEMNEPVQLTFALRYLNFFTKATPLSSTVTLSMSADVPLVVEYKIADMGHLKYY
LAPKIEDEEGSHHHHHH*

PCNA-G178S-H6
MFEARLVQGSILKKVLEALKDLINEACWDISSSGVNLQSMDSSHVSLVQLTLRSEGFDTYRC
DRNLAMGVNLTSMSKILKCAGNEDIITLRAEDNADTLALVFEAPNQEKVSDYEMKLMDLDVE
QLGIPEQEYSCVVKMPSGEFARICRDLSHIGDAVVISCAKDGVKFSASGELGNSNIKLSQTS
NVDKEEEAVTIEMNEPVQLTFALRYLNFFTKATPLSSTVTLSMSADVPLVVEYKIADMGHLKY
YLAPKIEDEEGSHHHHHH*

hRFC
see addgene #175049 and #175043

2. Chemical synthesis

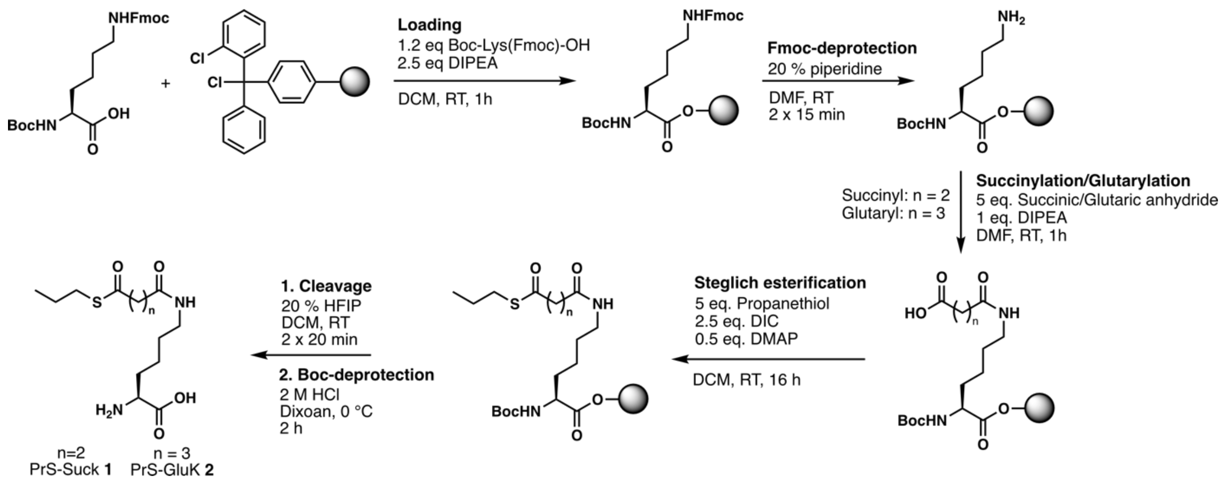

Scheme S1: Synthetic routes to amino acids PrS-SucK 1 and PrS-GluK 2. (Boc: tert-butyloxycarbonyl protecting group; Fmoc: fluorenylmethoxycarbonyl protecting group).

2-Chlorotrityl chloride (CTC)-resin (1 g, 1.20 mmol/g maximal loading capacity) was weighted into a syringe with a frit. Subsequently, Boc-Lys(Fmoc)-OH (1.2 eq., 1.44 mmol, 675 mg) and *N,N*-diisopropylethylamine (DIPEA) (2.5 eq., 3.60 mmol, 612 µL) in dichloromethane (DCM) (10 mL/g resin) were added to the syringe followed by shaking at room temperature (RT) for 1 h. Afterwards, the resin was washed five times with DCM (10 mL/g resin) and five times with dimethylformamide (DMF) (10 mL/g resin). For Fmoc-deprotection 20 % piperidine in DMF was added to the syringe followed by shaking at RT for 15 min. This procedure was repeated once for 15 min. Afterwards the syringe was washed five times with DMF (10 mL/g resin) and a solution of succinic anhydride (5 eq., 6.00 mmol, 600 mg) or glutaric anhydride (5 eq., 6.00 mmol, 684 mg) in DMF (10 mL/g resin) was added. This mixture was allowed to shake at RT for 1 h followed by five times washing with DMF (10 mL/g resin) and five times washing with DCM (10 mL/g resin). For Steglich esterification the syringe was charged with propanethiol (5 eq., 6.00 mmol, 544 µL), *N,N'*-diisopropylcarbodiimide (DIC) (1.5 eq., 1.80 mmol, 282 µL) and 4-Dimethylaminopyridine (DMAP) (0.5 eq., 0.60 mmol, 73.3 mg) in DCM and incubated at RT for 16 h. For cleavage the syringe was washed five times with DCM (10 mL/g resin) and charged with 20 % hexafluoroisopropanol (HFIP) in DCM (10 mL/g resin) followed by shaking for 10 min at RT. This procedure was repeated, and the filtrates were combined in a round bottom flask. The solvent was evaporated under reduced pressure, the product was dissolved in 2 M HCl in dioxane in an ice-water bath and stirred for 1 h at 0 °C. Afterwards the solvent was evaporated under reduced pressure and the product was dissolved in a small volume of MeOH followed by precipitation in ice-cold diethyl ether (Et_2O) in 50 mL centrifugal tubes. After centrifugation and washing of the precipitate with ice-cold Et_2O the product was dissolved in H_2O and lyophilized, which delivered N6-(4-oxo-4-(propylthio)butanoyl)-L-lysine (PrS-SucK **1**)/ N6-(5-oxo-5-(propylthio)pentanoyl)-L-lysine (PrS-GluK **2**) as a white solid. PrS-SucK/PrS-GluK was stored at -20 °C. Stock solutions were prepared by dissolving PrS-SucK/PrS-GluK in H_2O followed by neutralization with NaOH.

PrS-SucK:

^{1}H-NMR (400 MHz, DMSO-d_6): *δ* 8.45 (s, 2H), 7.98 (s, 1H), 3.82 (s, 1H), 3.01 (q, *J* = 6.3 Hz, 2H), 2.79 (dt, *J* = 11.1, 7.2 Hz, 4H), 2.37 (t, *J* = 7.1 Hz, 2H), 1.82-1.75 (m, 2H), 1.56-1.47 (m, 2H), 1.46-1.24 (m, 4H), 0.92-0.84 (m, 3H). **^{13}C NMR (125 MHz, DMSO-d_6):** δ 197.99, 170.98, 170.05, 51.80, 38.92, 38.12, 30.17, 29.97, 29.54, 28.46, 22.57, 21.68, 13.09.

HR-MS (ESI+): m/z
observed: 305.1523 $[M+H]^+$
calculated ($C_{13}H_{24}N_2O_4S$): 305.1530 $[M+H]^+$

PrS-GluK:

^{1}H-NMR (400 MHz, DMSO-d_6): δ 8.47 (s, 2H), 7.93 (t, J = 5.6 Hz, 1H), 3.81 (p, J = 5.9 Hz, 1H), 3.00 (q, J = 6.2 Hz, 2H), 2.80 (t, J = 7.1 Hz, 2H), 2.59-2.52 (m, 2H), 2.08 (t, J = 7.4 Hz, 2H), 1.86-1.66 (m, 4H), 1.51 (h, J = 7.3 Hz, 2H), 1.45-1.22 (m, 4H), 0.89 (t, J = 7.3 Hz, 3H).**^{13}C NMR (125 MHz, DMSO-d_6):** δ 198.55, 171.23, 170.94, 51.83, 42.93, 38.03, 34.08, 30.01, 29.56, 28.52, 22.62, 21.74, 21.32, 13.13.

HR-MS (ESI+): m/z
observed: 319.1681 $[M+H]^+$
calculated ($C_{14}H_{26}N_2O_4S$): 319.1686 $[M+H]^+$

3. 96-well based PylRS screen

A screen of approx. 200 PylRS variants available in the lab was performed using a 96-well setup with a fluorescence readout. Therefore, chemically competent *E. coli* K12 were co-transformed with pPylT_sfGFP-N150TAG-H6 (encoding either *Mb* or *Ma* $tRNA_{CUA}$ and the C-terminally H6-tagged sfGFP-N150TAG) and pBK_PylRS (encoding either the *Mb* or *Ma* PylRS) plasmids. After recovery with 0.2 mL of SOC medium (Super Optimal Broth medium containing 20 mM glucose) for 1 h at 37 °C, the cells were cultured overnight in 1.8 mL of non-inducing medium supplemented with tetracycline (17.5 µg/mL) and ampicillin (100 µg/mL) or kanamycin (25 µg/mL) at 37 °C, 200 rpm in a 96 deep well plate. After overnight incubation the cultures were diluted 1:100 into autoinduction medium supplemented with 2 mM PrS-SucK or 2 mM PrS-GluK and the respective antibiotics in a 96-well deep well plate. After incubation at 37 °C, 200 rpm for 16 h the cultures were diluted 1:10 into PBS (phosphate-buffered saline) and fluorescence (excitation: 485 nm, emission: 510 nm) was measured using a Varioskan LUX multimode microplate reader (Thermo Fischer Scientific).

4. Protein expression and purification

4.1 Expression and purification of tagless Ub

Chemically competent *E. coli* Rosetta2 (DE3) were transformed with pET17b-Ub plasmid (see 1.3, Supplementary Table S3). After recovery with 1 mL of SOC medium for 1 h at 37 °C, the cells were cultured overnight in 50 mL of 2× YT medium containing ampicillin (100 µg/mL) and chloramphenicol (50 µg/mL) at 37 °C, 200 rpm. The overnight culture was diluted to an OD_{600} (optical density at 600 nm) of 0.05 in 3 L of fresh 2× YT medium supplemented with ampicillin (50 µg/mL) and chloramphenicol (25 µg/mL) and cultured at 37 °C with shaking (200 rpm) until OD_{600} reached 0.8 - 1.0. IPTG (isopropyl β-D-1-thiogalactopyranoside) was added to a final concentration of 1 mM and protein expression was induced for 4 h at 37 °C. The cells were harvested by centrifugation (4000 × *g*, 20 min, 4 °C) and resuspended in lysis buffer (50 mM Tris pH 7.6, 10 mM $MgCl_2$, 1 mM EDTA (ethylenediaminetetraacetic acid), 0.1% NP-40, 0.1 mg/mL DNAse I, one cOmplete™ protease inhibitor tablet and 0.175 mg/mL PMSF (phenylmethylsulfonyl fluoride)). Cells were lysed by sonication with cooling in an ice-water bath and centrifuged (15,000 × *g*, 40 min, 4 °C). The cleared lysate was transferred into a glass beaker in an ice-water bath placed on a magnetic stirrer. Precipitation was performed with 35 % perchloric acid until pH 4.0 - 4.5 was reached. After 5 min incubation at 4 °C while stirring, the milky solution was centrifuged (15,000 × *g*, 40 min, 4 °C) and the supernatant was transferred into a dialysis tubing with a MWCO (molecular weight cut-off) of 2 kDa (Roth). Dialysis was performed overnight at 4 °C with 50 mM ammonium acetate buffer pH 4.5. The dialyzed solution was centrifuged (15,000 × *g*, 40 min, 4 °C), filtered and purified via a HiTrap SP FF 5 mL cation exchange chromatography (GE, gradient 0 - 1 M NaCl). Fractions that showed > 95% purity, as judged by sodium dodecyl sulfate-polyacrylamide gel electrophoresis (SDS-PAGE), were pooled, concentrated, and further purified via size-exclusion chromatography (SEC) using a Superdex Increase 75 10/300 (GE Healthcare) with a buffer containing 25 mM Tris pH 7.5 at 4 °C, 150 mM NaCl. Fractions containing pure Ub were pooled together and concentrated using Amicon® centrifugal filter units with a 3 kDa MWCO (Millipore). Protein concentration was calculated from the measured A280 absorption (extinction coefficients were calculated with ProtParam (https://web.expasy.org/protparam/)). In case of Ub the determination of protein concentration using the absorption at 280 nm is inaccurate (due to the low extinction coefficient (ε)), and therefore bicinchoninic acid assay

(BCA) (Thermo Scientific™) and Bradford (Sigma-Aldrich) assays were used for accurate protein concentration determination or the concentration of purified Ub was adjusted densiometrically. Purified Ub was flash frozen using liquid nitrogen and stored at -80 °C until further use.

4.2 Expression and purification of H6-tagged wt Ub/sfGFP/PCNA/AzoR/GAPDH and respective mutants

Chemically competent *E. coli* K12 were transformed with H6-tagged protein of interest (POI) in a respective plasmid (see 1.3, Supplementary Table S3). After recovery with 1 mL of SOC medium for 1 h at 37 °C, the cells were cultured overnight in 50 mL 2× YT medium containing the corresponding antibiotics (using the following concentrations: tetracycline 17.5 µg/mL, ampicillin 100 µg/mL, chloramphenicol 50 µg/mL) at 37 °C, 200 rpm. The overnight culture was diluted to an OD_{600} of 0.05 in 200 mL of fresh 2× YT medium supplemented with the corresponding antibiotics (using the following concentrations: tetracycline 8.75 µg/mL, ampicillin 50 µg/mL, chloramphenicol 25 µg/mL) and cultured at 37 °C, 200 rpm, until OD_{600} reached 0.8 - 1.0. arabinose was added to a final concentration of 0.02 % (w/v) and protein expression was induced for 16 h at 37 °C. The cells were harvested by centrifugation (4000 × *g*, 20 min, 4 °C) and resuspended in 20 mL of lysis buffer (50 mM Tris pH 7.5, 150 mM NaCl, 30 mM imidazole, 0.175 mg/mL PMSF, 0.1 mg/mL DNase I, and one cOmplete™ protease inhibitor tablet (Roche) unless stated otherwise in Methods section). The cell suspension was incubated on ice for 30 min and sonicated with cooling in an ice-water bath. The lysed cells were centrifuged (15,000 × *g*, 40 min, 4 °C), the cleared lysate added to Ni-NTA (Ni^{2+} nitrilotriacetic acid) slurry (Jena Bioscience) (1 mL of slurry per 1 L of culture) and the mixture was incubated with agitation for 1 h at 4 °C. After incubation, the mixture was transferred to an empty plastic column and washed with 10 CV (column volumes) of wash buffer (50 mM Tris pH 7.5, 150 mM NaCl, 30 mM imidazole unless stated otherwise in Methods section). The protein was eluted in 1 mL fractions with wash buffer supplemented with 300 mM imidazole. The fractions containing the protein were pooled, concentrated and further purified via SEC using a Superdex Increase 75 10/300 (GE Healthcare) with a buffer containing 25 mM Tris pH 7.5 at 4 °C, 150 mM NaCl. Fractions containing the pure proteins were pooled together and concentrated using Amicon® centrifugal filter units with a 3 kDa/10 kDa/30 kDa MWCO (Millipore). Protein concentration was calculated from the measured A280 absorption (extinction coefficients were calculated with ProtParam (https://web.expasy.org/protparam/)). In case of Ub the determination of protein concentration using the absorption at 280 nm is inaccurate (due to the low ε), and therefore BCA (Thermo Scientific™) and Bradford (Sigma-Aldrich) assays were used for accurate protein concentration determination or the concentration of purified Ub was adjusted densiometrically. Purified proteins were analyzed by 15 % SDS-PAGE and mass spectrometry and flash frozen using liquid nitrogen and stored at -80 °C until further use. Also, wt proteins and mutants were treated with the thioester-hydrolysis conditions and used in control experiments for downstream assays.

4.3 Expression and purification of wt Histone H3

The protocol for expression and purification of Histone H3 as well as Histone H3 variants bearing ncAAs was adapted from the literature[7]. Chemically competent *E. coli* DH10β were transformed with H6-tagged Histone H3 in a pBAD plasmid (see 1.3, Supplementary Table

S3). After recovery with 1 mL of SOC medium for 1 h at 37 °C, the cells were cultured overnight in 50 mL 2× YT medium containing ampicillin (100 µg/mL) at 37 °C, 200 rpm. The overnight culture was diluted to an OD_{600} of 0.05 in 200 mL of fresh 2× YT medium supplemented with ampicillin (50 µg/mL) and 20 mM nicotinamide (NAM) and cultured at 37 °C, 200 rpm, until OD_{600} reached 0.8 - 1.0. Arabinose was added to a final concentration of 0.2 % (w/v) and protein expression was induced for 16 h at 37 °C. The cells were harvested by centrifugation (4000 × *g*, 20 min, 4 °C) and resuspended in 20 mL of lysis buffer (50 mM HEPES pH 7.0, 500 mM NaCl, 30 mM imidazole, 20 mM NAM, 0.175 mg/mL PMSF, 0.1 mg/mL DNase I, and one cOmplete^{TM} protease inhibitor tablet (Roche)). The cell suspension was incubated on ice for 30 min and sonicated with cooling in an ice-water bath. The lysed cells were centrifuged (15,000 × *g*, 40 min, 4 °C), the supernatant was removed, and the pellet was resuspended in 20 mL 2 % triton and 20 mM NAM in water. The suspension was incubated on ice for 10 min and centrifuged (12000 × *g*, 20 min, 4 °C). The process of resuspending in 20 mL 2 % triton and 20 mM NAM in water, centrifugation, and incubation on ice was repeated and followed by an additional washing step with 50 mM HEPES pH 7.0, 500 mM NaCl, 20 mM NAM. After the washing steps, the pellet was resuspended in 50 mM HEPES pH 7.0, 500 mM NaCl, 20 mM NAM supplemented with 6 M Urea using ultrasonication. The suspension was centrifuged and the supernatant was added to Ni-NTA slurry (Jena Bioscience) (1 mL of slurry per 1 L of culture) and the mixture was incubated with agitation for 1 h at 4 °C. After incubation, the mixture was transferred to an empty plastic column and washed with 10 CV of wash buffer (50 mM HEPES pH 7.0, 500 mM NaCl, 30 mM imidazole, 20 mM NAM, 6 M Urea). The protein was eluted in 1 mL fractions with the wash buffer supplemented with 300 mM imidazole pH 7.0. The fractions containing the protein were pooled, concentrated and subjected to cation exchange chromatography using a Resource S column (Cytiva) with a gradient from 5 - 40 % B (Buffer A: 50 mM HEPES pH 7.0, 100 mM NaCl, 6 M Urea, 1 mM DTT (dithiothreitol), Buffer B: 50 mM HEPES pH 7.0, 1 M NaCl, 6 M Urea, 1 mM DTT). Fractions containing Histone H3 (identified via SDS-PAGE) were pooled and refolded by stepwise dialysis (6 M Urea to 0 M Urea in six steps) using Pur-A-Lyzer^{TM} (Sigma Aldrich) dialysis chambers with a 3.5 kDa cutoff at 4 °C into Histone storage buffer (50 mM Tris pH 7.5, 150 mM NaCl, 0.5 mM TCEP (tris(2-carboxyethyl)phosphine)). Finally, Histone H3 was concentrated using Amicon® centrifugal filter units (Millipore) with a 3 kDa MWCO. Protein concentration was calculated from the measured A280 absorption (extinction coefficients were calculated with ProtParam (https://web.expasy.org/protparam/)). Histone H3 was flash frozen in liquid nitrogen and stored at -80 °C until further use.

4.4 Expression and purification of hRFC

hRFC was expressed and purified as previously described in the literature with slight adaptions[8]. Chemically competent *E. coli* Rosetta II (DE3) were co-transformed with pCDF1b Replication factor C subunit 1 (Addgene #175043) and pET Duet1 Replication factor C subunits 2-5 (Addgene #175049). After recovery with 1 mL of SOC medium for 1 h at 37 °C, the cells were cultured overnight in 50 mL 2× YT medium containing kanamycin (50 µg/mL), ampicillin (100 µg/mL), and chloramphenicol (100 µg/mL) at 37 °C, 200 rpm. The overnight culture was diluted to an OD_{600} of 0.05 in 3 L of fresh TB medium supplemented with kanamycin (25 µg/mL), ampicillin (50 µg/mL) and chloramphenicol (50 µg/mL) and cultured at 37 °C with shaking (200 rpm) until OD_{600} reached 0.6. IPTG was added to a final concentration of 0.3 mM and protein expression was induced for 18 h at 20 °C. The cells were harvested by centrifugation (4000 × g, 30 min, 4 °C) and resuspended in lysis buffer (20 mM HEPES pH 7.4,

180 mM NaCl, 2 mM EDTA, 5 % Glycerol, 0.01 % IGEPAL, 2 mM DTT and a cOmplete™ protease inhibitor tablet (Roche)). The cell suspension was incubated on ice for 30 min and sonicated with cooling in an ice-water bath. The lysed cells were centrifuged (15,000 × g, 40 min, 4 °C) and the supernatant subjected to cation exchange chromatography using a HiTrap SP HP (Cytiva) with a gradient 0 % - 100 % B (Buffer A: 20 mM HEPES pH 7.4, 200 mM NaCl, 2 mM EDTA, 5 % Glycerol, 0.01 % IGEPAL, 2 mM DTT, Buffer B: Buffer A: 20 mM HEPES pH 7.4, 1000 mM NaCl, 2 mM EDTA, 5 % Glycerol, 0.01 % IGEPAL, 2 mM DTT). Fractions containing all hRFC subunits (identified via SDS-PAGE) were pooled and concentrated using Amicon® centrifugal filter units (Millipore). Afterwards, crude hRFC1-5 was further purified using a CHT column with Buffer A: 50 mM KPO_4 buffer pH 7.4, 100 mM NaCl, 5 % Glycerol, 0.01 % IGEPAL and Buffer B: 750 mM KPO_4 buffer pH 7.4. Therefore, crude hRFC1-5 was diluted into CHT buffer A and loaded onto a CHT ceramic hydroxyapatite column (EconoFit CHT Type II, 40 µm Column, BioRad). After a 10 CV wash with CHT buffer A, the column was washed with a gradient from 0 % - 13 % (8 CV) CHT buffer. Afterwards the protein was eluted using a gradient from 13 % - 100 %. Fractions containing all hRFC subunits (identified via SDS-PAGE) were pooled and rebuffered into SEC buffer (25mM HEPES pH 7.4, 300 mM NaCl, 15 % Glycerol, 0.01 % NP-40 and 2 mM DTT) using Amicon® centrifugal filter units (Millipore) and further purified via SEC using a Superose 6 Increase 10/300 GL (Cytiva) with SEC buffer. Fractions containing all hRFC subunits (of a monodisperse peak, identified via SDS-PAGE) were combined, concentrated to 8 mg/mL flash frozen in liquid nitrogen and stored at -80 °C until further use. Protein concentration was calculated from the measured A280 absorption (extinction coefficients were calculated with ProtParam (https://web.expasy.org/protparam/)).

4.5 Expression and purification of UBE2S

Chemically competent *E. coli* BL21 (DE3) were transformed with pOPINK-UBE2S (encoding for GST-UBE2S). After recovery with 1 mL of SOC medium for 1 h at 37 °C, the cells were cultured overnight in 50 mL 2× YT medium containing kanamycin (50 µg/mL) at 37 °C, 200 rpm. The overnight culture was diluted to an OD_{600} of 0.05 in 1 L of fresh 2× YT medium supplemented with kanamycin (25 µg/mL) and cultured at 37 °C with shaking (200 rpm) until OD_{600} reached 0.8. IPTG was added to a final concentration of 0.25 mM and protein expression was induced for 18 h at 20 °C. The cells were harvested by centrifugation (4000 × g, 20 min, 4 °C) and resuspended in lysis buffer (50 mM Tris pH 8.0, 300 mM sucrose, 50 mM NaF, 2 mM DTT, 0.1 mg/mL DNase I, and one cOmplete™ protease inhibitor tablet (Roche)). The cell suspension was incubated on ice for 30 min followed by lysis using a cell disruptor (Constant Systems CF2). The lysed cells were centrifuged (15,000 × g, 40 min, 4 °C), the cleared lysate added to Glutathione Sepharose 4B (GE Healthcare) (0.1 mL of slurry per 100 mL of culture) and the mixture was incubated with agitation for 1 h at 4 °C. After incubation, the mixture was transferred to an empty plastic column and washed with 10 CV of wash buffer (25 mM Tris pH 8.5, 400 mM NaCl, 5 mM DTT). On beads GST-cleavage was performed by addition of PreScission Protease (Sigma-Aldrich, Cat. No. GE27-0843-01) in wash buffer, followed by incubation at 4 °C for 3 h. The flow through was collected and applied to SEC using a Superdex S75 16/600 (Cytiva) with SEC buffer (50 mM Tris pH 7.5, 150 mM NaCl, 1 mM DTT). Fractions containing the untagged UBE2S were pooled and concentrated with Amicon® centrifugal filter units with an appropriate MWCO (Millipore). Protein concentration was calculated from the measured A280 absorption (extinction coefficients were calculated with

ProtParam (https://web.expasy.org/protparam/)). UBE2S was flash frozen using liquid nitrogen and stored at -80 °C until further use.

4.6 Expression and purification of Cezanne

Chemically competent *E. coli* Rosetta II (DE3) were transformed with pOPINK-Cezanne Isoform1 (OTU, aa 53-446) (encoding for GST-Cezanne). After recovery with 1 mL of SOC medium for 1 h at 37 °C, the cells were cultured overnight in 50 mL of 2× YT medium containing kanamycin (50 µg/mL) and chloramphenicol (100 µg/mL) at 37 °C, 200 rpm. The overnight culture was diluted to an OD_{600} of 0.05 in 1 L of fresh 2× YT medium supplemented with kanamycin (25 µg/mL) and chloramphenicol (50 µg/mL) and cultured at 37 °C with shaking (200 rpm) until OD_{600} reached 0.8. IPTG was added to a final concentration of 0.25 mM and protein expression was induced for 18 h at 16 °C. The cells were harvested by centrifugation (4000 × g, 20 min, 4 °C) and resuspended in lysis buffer (25 mM Tris pH 7.5, 200 mM NaCl, 1 mM BME and one cOmplete^{TM} protease inhibitor tablet (Roche)). The cell suspension was incubated on ice for 30 min and sonicated with cooling in an ice-water bath. The lysed cells were centrifuged (15,000 × g, 40 min, 4 °C), the cleared lysate added to Glutathione Sepharose 4B (GE Healthcare) (0.1 mL of slurry per 100 mL of culture) and the mixture was incubated with agitation for 1 h at 4 °C.

After incubation, the mixture was transferred to an empty plastic column and washed with 40 CV of high salt wash buffer (25 mM Tris pH 7.5, 500 mM NaCl, 1 mM BME) followed by washing with low salt buffer (25 mM Tris pH 7.5, 100 mM NaCl, 5 mM DTT). On beads GST-cleavage was performed by addition of PreScission Protease (Sigma-Aldrich, Cat. No. GE27-0843-01) in low salt wash buffer, followed by incubation at 4 °C for 16 h. The flow through was collected and applied to SEC using a Superdex S75 10/300 (Cytiva) with SEC buffer (25 mM Tris pH 7.5, 200 mM NaCl, 1 mM DTT). Fractions containing the untagged Cezanne Isoform1 were pooled and concentrated with Amicon® centrifugal filter units with an appropriate MWCO (Millipore). Protein concentration was calculated from the measured A280 absorption (extinction coefficients were calculated with ProtParam (https://web.expasy.org/protparam/)). Cezanne Isoform1 was flash frozen using liquid nitrogen and stored at -80 °C until further use.

4.7 Expression and purification of CobB

Chemically competent *E. coli* BL21 (DE3) were transformed with pGEX-CobB (encoding for GST-thrombin-TEV-CobB). After recovery with 1 mL of SOC medium for 1 h at 37 °C, the cells were cultured overnight in 50 mL of 2× YT medium containing ampicillin (100 µg/mL) at 37 °C, 200 rpm. The overnight culture was diluted to an OD_{600} of 0.05 in 1.5 L of fresh 2× YT medium supplemented with ampicillin (50 µg/mL) and cultured at 37 °C with shaking (200 rpm) until OD_{600} reached 0.8 - 1.0. IPTG was added to a final concentration of 0.2 mM and protein expression was induced for 18 h at 16 °C. The cells were harvested by centrifugation (4000 × *g*, 20 min, 4 °C) and resuspended in 20 mL of lysis buffer (50 mM Tris pH 7.4 at 4 °C, 100 mM NaCl, 5 mM $MgCl_2$, 0.5 mM DTT, 0.1 mM PMSF). The cell suspension was incubated on ice for 30 min and sonicated with cooling in an ice-water bath. The lysed cells were centrifuged (15,000 × g, 40 min, 4 °C), the cleared lysate added to Glutathione Sepharose 4B (GE Healthcare) (0.1 mL of slurry per 100 mL of culture) and the mixture was incubated with agitation for 1 h at 4 °C. After incubation, the mixture was transferred to an empty plastic column and washed with 10 CV of wash buffer (50 mM Tris pH 7.4 at 4 °C, 100 mM NaCl,

5 mM $MgCl_2$, 0.5 mM DTT). CobB was eluted in 1 mL fractions with a buffer containing 50 mM Tris pH 7.4 at 4 °C, 100 mM NaCl, 5 mM $MgCl_2$, 0.5 mM DTT, 10 mM GSH (glutathione). The fractions containing the protein were pooled, rebuffered to a buffer containing 50 mM Tris pH 7.4 at 4 °C, 100 mM NaCl, 5 mM $MgCl_2$, 0.5 mM DTT, and concentrated using Amicon® centrifugal filter units with a 30 kDa MWCO (Millipore). Protein concentration was calculated from the measured A280 absorption (extinction coefficients were calculated with ProtParam (https://web.expasy.org/protparam/)). Purified proteins were analyzed by 15% SDS-PAGE and mass spectrometry and flash frozen using liquid nitrogen and stored at −80 °C until further use.

4.8 Expression and purification of SIRT5

Chemically competent *E. coli* BL21 (DE3) were transformed with pET17-SIRT5 (encoding for H6-TEV-SIRT5). After recovery with 1 mL of SOC medium for 1 h at 37 °C, the cells were cultured overnight in 50 mL of 2× YT medium containing ampicillin (100 µg/mL) at 37 °C, 200 rpm. The overnight culture was diluted to an OD_{600} of 0.05 in 1.5 L of fresh 2× YT medium supplemented with ampicillin (50 µg/mL) and cultured at 37 °C with shaking (200 rpm) until OD_{600} reached 0.8 - 1.0. IPTG was added to a final concentration of 1 mM and protein expression was induced for 16 h at 20 °C. The cells were harvested by centrifugation (4000 × *g*, 20 min, 4 °C) and resuspended in 20 mL of lysis buffer (50 mM Na_2HPO_4/NaH_2PO_4 pH 8.0, 300 mM NaCl, 20 mM imidazole). The cell suspension was incubated on ice for 30 min and sonicated with cooling in an ice-water bath. The lysed cells were centrifuged (15,000 × *g*, 40 min, 4 °C), the cleared lysate added to Ni-NTA slurry (Jena Bioscience) (1 mL of slurry per 1 L of culture) and the mixture was incubated with agitation for 1 h at 4 °C. After incubation, the mixture was transferred to an empty plastic column and washed with 10 CV of wash buffer (50 mM Na_2HPO_4/NaH_2PO_4 pH 8.0, 300 mM NaCl, 20 mM imidazole). The protein was eluted in 1 mL fractions with a buffer containing 50 mM Na_2HPO_4/NaH_2PO_4 pH 8.0, 300 mM NaCl, 250 mM imidazole, and 5 % (v/v) glycerol. The fractions containing the protein were pooled, rebuffered to a buffer containing 50 mM Na_2HPO_4/NaH_2PO_4 pH 8.0, 300 mM NaCl, and 5 % (v/v) glycerol, and concentrated using Amicon® centrifugal filter units with a 30 kDa MWCO (Millipore). Protein concentration was calculated from the measured A280 absorption (extinction coefficients were calculated with ProtParam (https://web.expasy.org/protparam/)). Purified proteins were analyzed by 15 % SDS-PAGE and mass spectrometry and flash frozen using liquid nitrogen and stored at -80 °C until further use.

4.9 Protein purification yields

Supplementary Table S4: Protein purification yields (per liter of culture for purified proteins).

**Protein**	**Yield**
sfGFP-wt	132-146 mg/L
sfGFP-N150BocK	92-110 mg/L
sfGFP-N150SucK	24-28 mg/L
sfGFP-N150GluK	34-40 mg/L

sfGFP-N150SucK (*Ma*)	60-68 mg/L
sfGFP-N150GluK (*Ma*)	43-59 mg/L
H3-wt	12-15 mg/L
H3-K122GluK	6-7 mg/L
H3-K122SucK	2-3 mg/L
GAPDH-wt	45-58 mg/L
GAPDH-K194GluK	7-13 mg/L
GAPDH-K194AcK	35-37 mg/L
GAPDH-K194E	40-42 mg/L
Ub-wt	20-25 mg/L
Ub-K33AcK	10-13 mg/L
Ub-K33GluK	10-15 mg/L
Ub-K33SucK	9-12 mg/L
PCNA-wt	32-48 mg/L
PCNA-K164SucK	2-5 mg/L
PCNA-K164GluK	5-7 mg/L
PCNA-K13SucK	1-2 mg/L
PCNA-K13GluK	1-3 mg/L
PCNA-K13AcK	1-3 mg/L
PCNA-K13E	20-21 mg/L

5. LC-MS and NMR

LC-MS analysis of small molecules was performed on an Agilent 1260 Infinity Series LC system with an Agilent 6210 ESI Single Quadrupole mass spectrometer using a Luna® C18(2)-HST (2 mm, 100 mm, 100 Å, 2.5 µm) capillary column (Phenomenex, Torrence, USA). The analysis was performed at RT with a flow rate of 360 µL/min and a gradient of 15-95 % solvent B in 3.5 min (solvent A: 0.1 % formic acid (FA) in water, solvent B 0.1 % FA in acetonitrile (ACN)).

LC-MS analysis of full-length proteins was performed on an Agilent 1260 Infinity Series LC system with an Agilent 6210 ESI Single Quadrupole mass spectrometer using a Jupiter C4 column (2 mm, 150 mm, 300 Å, 5 µm) capillary column (Phenomenex, Torrence, USA). The analysis was performed at RT with a flow rate of 900 µL/min and a gradient of 10 %-55 % solvent B in 1.65 min, followed by a gradient of 55 %-90 % solvent B in 0.85 min (solvent A: 0.1 % FA in water, solvent B 0.1 % FA in ACN).

Alternatively, LC-MS analysis of full-length proteins was performed on a Waters Xevo G2-XS QTof using a Acquity UPLC Protein BEH C4 (2.1 mm x 50 mm, 300 Å, 1.7 µm) column (Waters). The analysis was performed at RT with a flow rate of 500 µl/min and a gradient of 5-95 % solvent B (solvent A: 0.1 % FA in water, solvent B 0.1 % FA in ACN).

Data generated on the Agilent LC-MS was analyzed with OpenLab ChemStation (Agilent), data generated on the Waters instrument were analyzed with MassLynx (Waters).

HR-MS

For HR-MS analysis samples were submitted to the Molecular and Biomolecular Analysis Service MoBiAS (ETH Zurich) and analyzed on a Bruker Daltonics maXis ESI-QTOF.

NMR

^{1}H spectra were measured on Bruker Avance III HD 300 MHz (300 MHz for ^{1}H-NMR), Bruker Avance III 400 UltraShield (400 MHz for ^{1}H-NMR), or Bruker Avance-I AV500 UltraShield (500 MHz for ^{1}H-NMR). The spectra were analyzed using MestReNova, Version 14.3.1-31739 (Mestrelab Research S.L.). The spectra were referenced to the respective solvent to determine the chemical shift (δ) in ppm (DMSOd$_6$: 2.54, CD_3OD: 3.34, $CDCl_3$: 7.26547. The coupling constants are given in Hertz (Hz) and signal multiplicity is characterized as follows: s (singlet), d (doublet), t (triplet), q (quartet), qint (quintet), m (multiplet), br (broad), and combinations thereof.

Author Contributions

K.L. and M.F conceived the research plan and experimental strategy. M.L.J. synthesised ncAAs and performed PylRS screens to identify ThioRS, incorporated PrS-SucK/GluK into sfGFP, AzoR and GAPDH, optimized on-protein thioester hydrolysis, expressed and purified SIRT5 and CobB, performed deacylation assays as well as initial GAPDH assays. M.W. synthesised PrS-SucK/GluK, expressed and purified histones, GAPDH, ubiquitin and PCNA bearing site-specific SucK/GluK and optimized on-protein thioester hydrolysis. M.W. performed GAPDH assays, generated diUbs and performed DUB assays and conducted anisotropy measurement for DNA clamp loading. T.A.N. synthesised initial ncAAs and optimized hydrolysis conditions. M.F. expressed and purified Ube2S, Cezanne and hRFC and assisted in daily lab supervision. All authors analyzed data, and K.L. wrote the paper with input from the other authors.

Supplementary References

1 Jing, Y., Liu, Z. & Li, X. D. Protocol for the preparation of site-specific succinylated histone mimics to investigate the impact on nucleosome dynamics. *STAR Protoc* **2**, 100604 (2021).

2 Wang, Z. A. *et al.* A Versatile Approach for Site-Specific Lysine Acylation in Proteins. *Angew Chem Int Ed Engl* **56**, 1643-1647 (2017).

3 Davey, C. A., Sargent, D. F., Luger, K., Maeder, A. W. & Richmond, T. J. Solvent mediated interactions in the structure of the nucleosome core particle at 1.9 a resolution. *J Mol Biol* **319**, 1097-1113 (2002).

4 Ito, K. *et al.* Expansion of substrate specificity and catalytic mechanism of azoreductase by X-ray crystallography and site-directed mutagenesis. *J Biol Chem* **283**, 13889-13896 (2008).

5 Schneider, C. A., Rasband, W. S. & Eliceiri, K. W. NIH Image to ImageJ: 25 years of image analysis. *Nat Methods* **9**, 671-675 (2012).

6 Chiu, J., Tillett, D., Dawes, I. W. & March, P. E. Site-directed, Ligase-Independent Mutagenesis (SLIM) for highly efficient mutagenesis of plasmids greater than 8kb. *J Microbiol Methods* **73**, 195-198 (2008).

7 Tian, H. *et al.* Genetically Encoded Benzoyllysines Serve as Versatile Probes for Interrogating Histone Benzoylation and Interactions in Living Cells. *ACS Chem Biol* **16**, 2560-2569 (2021).

8 Gaubitz, C. *et al.* Structure of the human clamp loader reveals an autoinhibited conformation of a substrate-bound AAA+ switch. *Proc Natl Acad Sci U S A* **117**, 23571-23580 (2020).

Fully uncropped and unprocessed gels

red boxes are shown as cropped gels in Fig. S2a

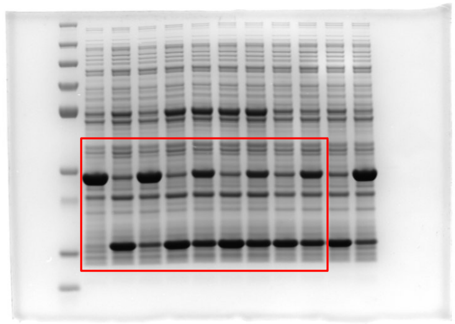

coomassie

red boxes are shown as cropped gels in Fig. S2b

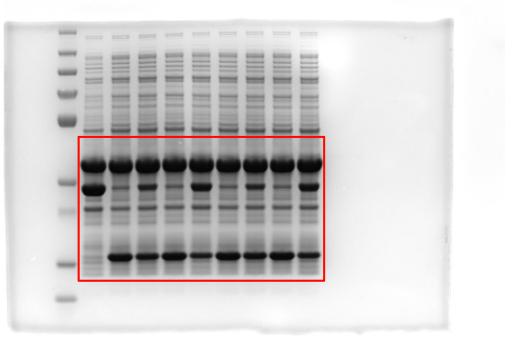

coomassie

red boxes are shown as cropped gels in Fig. S4b

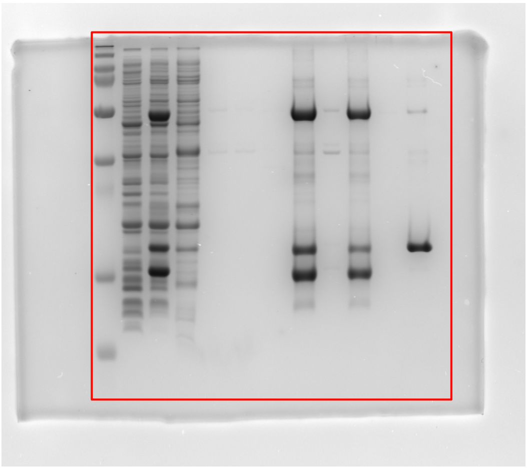

coomassie

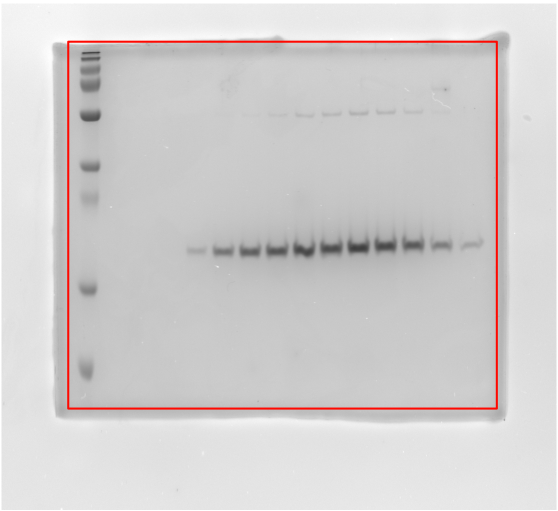

coomassie

red boxes are shown as cropped gels in Fig. S5b

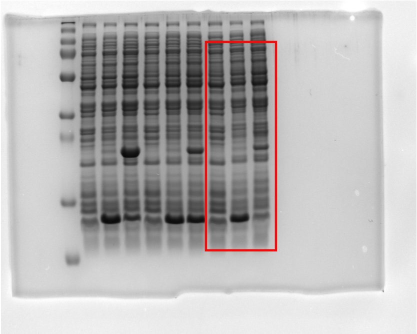

coomassie

red boxes are shown as cropped gels in Fig. S5c

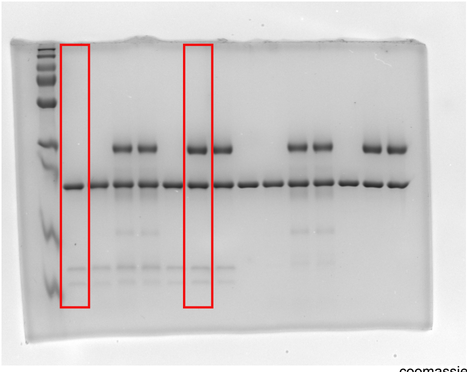

coomassie

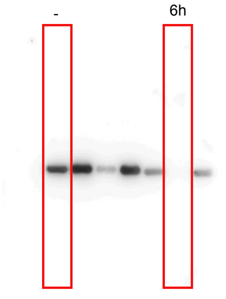

chemiluminescence

red boxes are shown as cropped gels in Fig. S5d

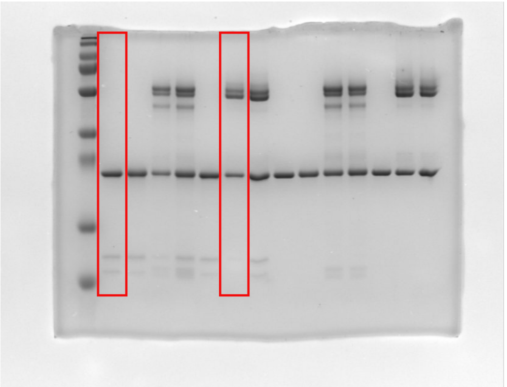

coomassie

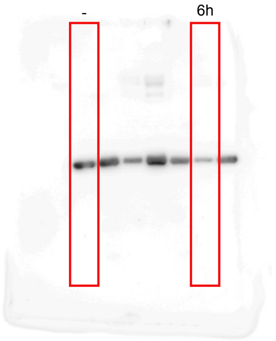

chemiluminescence

red boxes are shown as cropped gels in Fig. S6b

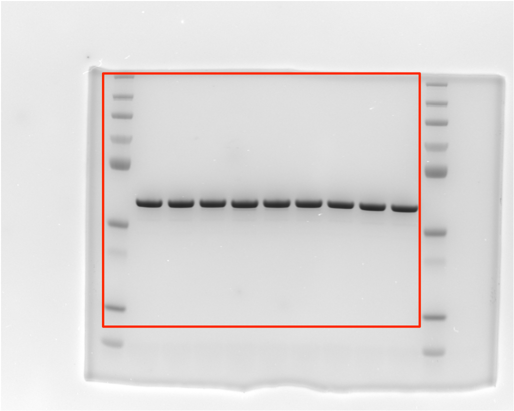

coomassie

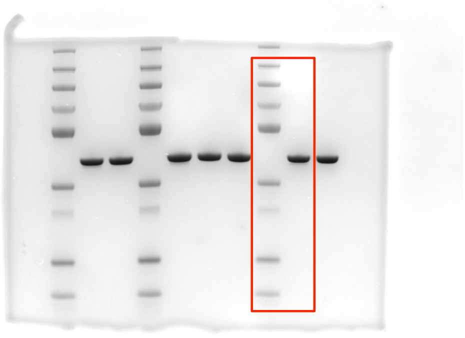

coomassie

red boxes are shown as cropped gels in Fig. S9a

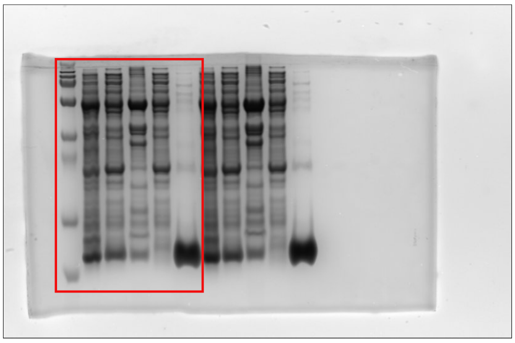

coomassie

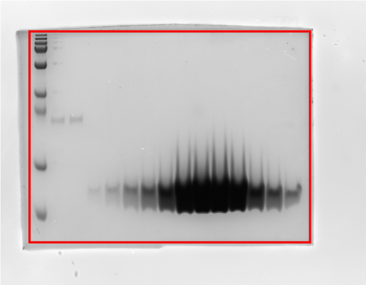

coomassie

red boxes are shown as cropped gels in Fig. S9d

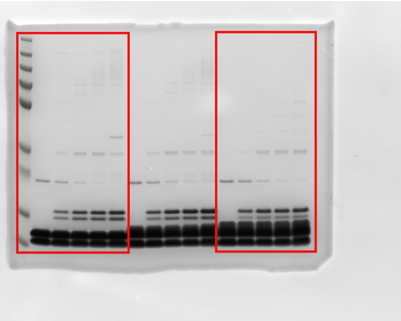

coomassie

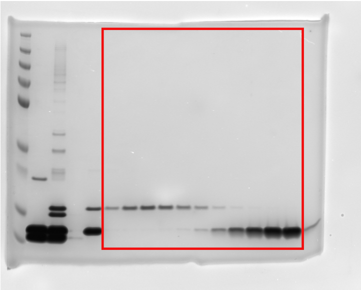

coomassie

red boxes are shown as cropped gels in Fig. S10a

wt K11-diUb-H_6

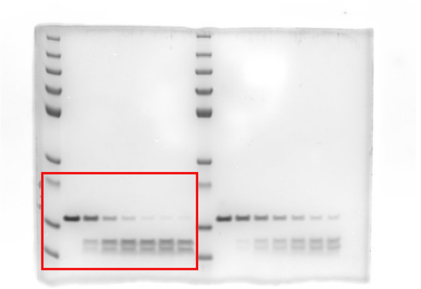

coomassie

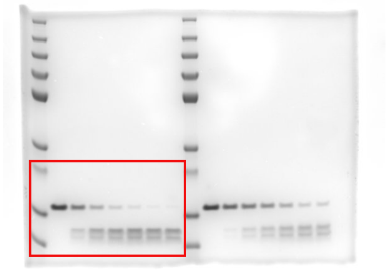

coomassie

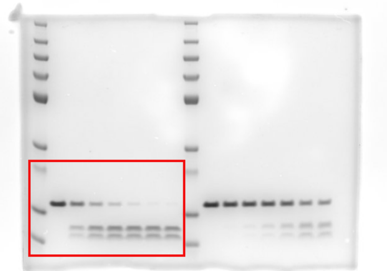

coomassie

K11-diUb(K33AcK)-H_6

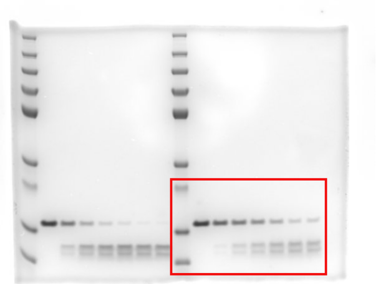

coomassie

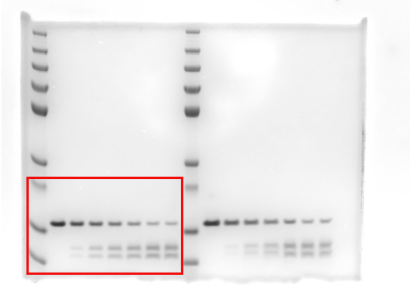

coomassie

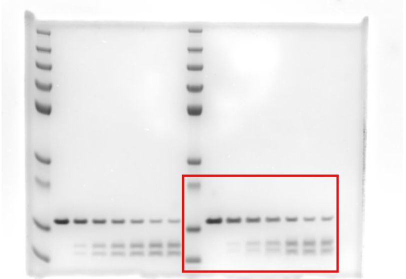

coomassie

K11-diUb(K33SucK)-H_6

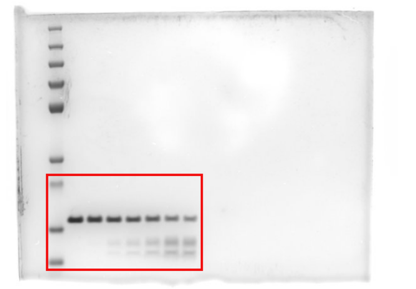

coomassie

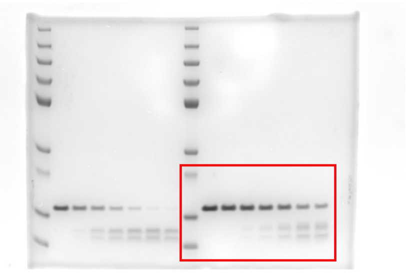

coomassie

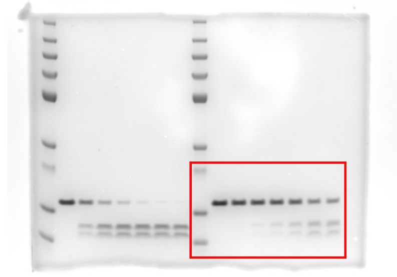

coomassie

red box is shown as cropped gel in Fig. S11a (adjusted for layout in the figure)

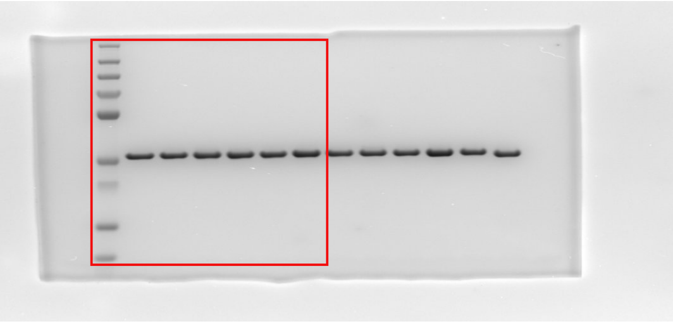

coomassie

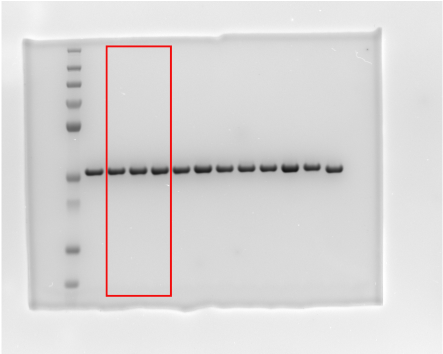

coomassie

red boxes are shown as cropped gels in Fig. S11b

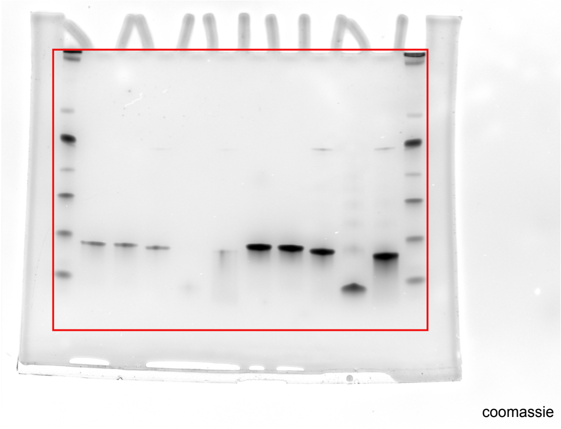

coomassie
