# Supplementary material for: Deciphering functional roles of protein succinylation and glutarylation using genetic code expansion
Source: Nat Chem. 2024 Mar 26;16(6):913–21. doi: 10.1038/s41557-024-01500-5 (PMC11164685; doi:10.1038/s41557-024-01500-5)
Supplement: Supplementary file 2 — Reporting Summary [file 41557_2024_1500_MOESM2_ESM.pdf]

nature portfolio

Corresponding author(s): Prof. Dr. Kathrin Lang
Dr. Maximilian Fottner

Last updated by author(s): Jan 29, 2024

Reporting Summary

Nature Portfolio wishes to improve the reproducibility of the work that we publish. This form provides structure for consistency and transparency in reporting. For further information on Nature Portfolio policies, see our Editorial Policies and the Editorial Policy Checklist.

Statistics

For all statistical analyses, confirm that the following items are present in the figure legend, table legend, main text, or Methods section.

n/a	Confirmed	
☐	☒	The exact sample size (n) for each experimental group/condition, given as a discrete number and unit of measurement
☐	☒	A statement on whether measurements were taken from distinct samples or whether the same sample was measured repeatedly
☒	☐	The statistical test(s) used AND whether they are one- or two-sided<br>*Only common tests should be described solely by name; describe more complex techniques in the Methods section.*
☒	☐	A description of all covariates tested
☒	☐	A description of any assumptions or corrections, such as tests of normality and adjustment for multiple comparisons
☐	☒	A full description of the statistical parameters including central tendency (e.g. means) or other basic estimates (e.g. regression coefficient) AND variation (e.g. standard deviation) or associated estimates of uncertainty (e.g. confidence intervals)
☒	☐	For null hypothesis testing, the test statistic (e.g. F, t, r) with confidence intervals, effect sizes, degrees of freedom and P value noted<br>*Give P values as exact values whenever suitable.*
☒	☐	For Bayesian analysis, information on the choice of priors and Markov chain Monte Carlo settings
☒	☐	For hierarchical and complex designs, identification of the appropriate level for tests and full reporting of outcomes
☒	☐	Estimates of effect sizes (e.g. Cohen's d, Pearson's r), indicating how they were calculated

Our web collection on statistics for biologists contains articles on many of the points above.

Software and code

Policy information about availability of computer code

Data collection	LC-MS: OpenLab ChemStation (Agilent, LTS01.11 (251)), MassLynx (Waters, 4.2 SCN1018)<br>Gels and Western Blots: iBright Imager TM Smart Digital Imaging (Thermo Fischer Scientific, 1.8.1.), Amersham ImageQuant 800 Control Software (Cytiva, 2.0.0)<br>Platereader: SPARKCONTROL Dashboard and Method Editor (Tecan, 3.2)<br>Fluorescence Anisotropy: Spectra Manager (Jasco, 2.15.05)<br>NMR: BrukerTopspin (Bruker, 4.0.5)
Data analysis	LC-MS: OpenLab ChemStation (Agilent, LTS01.11 (251)), MassLynx (Waters, 4.2 SCN1012)<br>Platerader and Fluorescence Anisotropy: GraphPad Prism (GraphPad Software, 10.1.1 (270) for Mac OS)<br>NMR: MestReNova (Mestrelab Research, 15.0.0-34764)<br>Gels and Western Blots: ImageJ (1.53k)

For manuscripts utilizing custom algorithms or software that are central to the research but not yet described in published literature, software must be made available to editors and reviewers. We strongly encourage code deposition in a community repository (e.g. GitHub). See the Nature Portfolio guidelines for submitting code & software for further information.

Data

Policy information about availability of data

All manuscripts must include a data availability statement. This statement should provide the following information, where applicable:

- Accession codes, unique identifiers, or web links for publicly available datasets
- A description of any restrictions on data availability
- For clinical datasets or third party data, please ensure that the statement adheres to our policy

The data generated or analyzed during this study are included in this article or its supplementary information files . Protein structures and models used for the figures are available under the accession codes: 1kx5, 1znq, 5lrv, 1sxj and 6gis. Source data are provided with this paper.

Human research participants

Policy information about studies involving human research participants and Sex and Gender in Research.

Reporting on sex and gender	not applicable
Population characteristics	not applicable
Recruitment	not applicable
Ethics oversight	not applicable

Note that full information on the approval of the study protocol must also be provided in the manuscript.

Field-specific reporting

Please select the one below that is the best fit for your research. If you are not sure, read the appropriate sections before making your selection.

☒ Life sciences ☐ Behavioural & social sciences ☐ Ecological, evolutionary & environmental sciences

For a reference copy of the document with all sections, see nature.com/documents/nr-reporting-summary-flat.pdf

Life sciences study design

All studies must disclose on these points even when the disclosure is negative.

Sample size	No statistical methods were used to pre-determine sample size. For determination of binding constants all samples were tested in three replicates. Sample size calculation was not necessary since the variation in the assays used is small.
Data exclusions	no data was excluded
Replication	For determination of binding constants all samples were tested in three biological replicates. All attempts at replication were successful.
Randomization	Randomization was not performed since the samples are from defined groups.
Blinding	Blinding was not performed since it is not relevant for the study. The groups were defined and studied by the investigators using stanadrad protocols.

Reporting for specific materials, systems and methods

We require information from authors about some types of materials, experimental systems and methods used in many studies. Here, indicate whether each material, system or method listed is relevant to your study. If you are not sure if a list item applies to your research, read the appropriate section before selecting a response.

Materials & experimental systems

n/a	Involved in the study	
☐	☒	Antibodies
☒	☐	Eukaryotic cell lines
☒	☐	Palaeontology and archaeology
☒	☐	Animals and other organisms
☒	☐	Clinical data
☒	☐	Dual use research of concern

Methods

n/a	Involved in the study	
☒	☐	ChIP-seq
☒	☐	Flow cytometry
☒	☐	MRI-based neuroimaging

Antibodies

Antibodies used

anti-SucK (mouse IgG, PTM Biolabs, PTM-419), dilution 1:1000, monoclonal antibody from mouse clone 3D3, cross reactivity validated by the manufacturer via dot plots using succinylated, acetylated, propionylated, butyrylated and crotonylated proteins.
anti-GluK (mouse IgG, PTM Biolabs, PTM-1152), dilution 1:1000, monoclonal antibody from mouse, cross reactivity validated by the manufacturer via dot plots using glutarylated, succinylated and malonylated proteins.
anti-mouse IgG peroxidase-coupled (Sigma-Aldrich, A4416), dilution 1:10000

Validation

All antibodies were sourced commercially and have been used in multiple published research articles. Validation of anti-SucK and antki-GluK antibodies is described above.
